# Mapping quantitative trait loci regions associated with Marek’s disease on chicken autosomes by means of selective DNA pooling

**DOI:** 10.1038/s41598-024-83356-w

**Published:** 2024-12-30

**Authors:** Ehud Lipkin, Jacqueline Smith, Morris Soller, David W. Burt, Janet E. Fulton

**Affiliations:** 1 Department of Genetics, The Alexander Silberman Institute of Life Sciences, The Hebrew University of Jerusalem, Edmond J. Safra Campus, Givat Ram, Jerusalem, 91904 Israel; 2 The Roslin Institute and Royal (Dick) School of Veterinary Studies R(D)SVS, University of Edinburgh, Easter Bush, Midlothian, EH25 9RG UK; 3 Hy-Line International, 2583 240th St, PO Box 310, Dallas Center, 50063 IA USA

**Keywords:** Marek’s Disease, Disease resistance, QTLR, Linkage disequilibrium, Allele substitution effect, Chicken, Agricultural genetics, Animal breeding, Genetic linkage study, Genetic markers, Genomics, Genotype, Haplotypes, Quantitative trait

## Abstract

Marek’s Disease (MD), which can result in neurological damage and tumour formation, has large effects on the economy and animal welfare of the poultry industry worldwide. Previously, we mapped autosomal MD QTL regions (QTLRs) by individual genotyping of an F_6_ population from a full-sib advanced intercross line. We further mapped MD QTLRs on the chicken Z chromosome (GGZ) using the same F_6_ population, and by selective DNA pooling (SDP) of 8 elite egg production lines. Here we used SDP of the same pools used on GGZ to map autosomal MD QTLRs. Thirty-seven QTLRs were found. Seven of the QTLRs were tested by all sires from the same 8 lines, individually genotyped for QTLR markers. Five of the tested QTLRs were confirmed. Linkage disequilibrium (LD) was calculated for all QTLR markers on the same chromosome, and complex LD blocks were found. Distribution of *P* and LD values were used to assess the QTLR causative elements. Allele substitution effects were calculated based on both pooled SNP microarray genotypes, and individual genotypes of QTLRs markers. Substantial allele effect and contribution to the phenotypic and genotypic variation were obtained. The results explain part of the MD response, and provide targets for mitigating MD.

**Supplementary Information:**

The online version contains supplementary material available at 10.1038/s41598-024-83356-w.

## Introduction

Marek’s Disease (MD) results in an annual loss of 2 billion USD to the global poultry industry through substantial mortality, decreased production and costs of vaccination^1^. MD is caused by the Marek’s Disease Virus (MDV), an immunosuppressive cell-associated oncogenic alpha herpesvirus^2^. Symptoms include depression, paralysis due to the involvement of the peripheral nervous system, appetite loss, weight loss, anemia, diarrhea and dehydration. Highly immunosuppressive, MDV leaves surviving birds susceptible to secondary infections^3^. Infection results in an early cytolytic phase in lymphoid cells, followed by a latent period when virus infects T-cells, which can become transformative with the emergence of T-cell lymphomas^4^.

The first line of defense against pathogens is the innate immune system, which also plays a fundamental role in the activation, regulation, and orientation of the adaptive immune response^5^. Currently, the disease is controlled by the use of vaccines. These vaccines prevent the formation of tumors but do not prevent MDV infection or shedding of the pathogenic virus^6^. Pathogenic MDVs found in vaccinated flocks can result in the emergence of increasingly more virulent strains^7^. Vaccine treatments then in turn become less and less effective^8^, and an escalating feedback loop of increasing MD pathogenicity results.

The emergence of these new virus strains, as well as reduced vaccine effectiveness calls for other means of control, including genetic improvement. Over the years, successful genomic selection on specific traits has become widely used in both plants and animals^9^. Genetic selection for increased host viral resistance towards MDV has already been applied and has improved survival in commercial chicken populations^10^. However, in the long term, the truncation selection involved in genomic selection could reduce the full potential of genetic value by up to 40%, due to loss of favorable alleles of quantitative trait loci (QTL)^11^. Identification of the causative genomic elements and variants associated with a trait can keep the variation and prevent that loss. This can be achieved by QTL mapping.

Previous studies to map genomic elements responsible for MD resistance have had limited success. It is clear that MD resistance involves many elements, most with relatively small effect, thus making it difficult to identify the causal elements^12^. The exception is the chicken Major Histocompatibility Complex (MHC) on chromosome 16, which has been shown to have a major role in disease resistance to multiple bacterial and viral pathogens, including MDV^13^. In light of this role, mapping other MD QTLs must account for the MHC background, to avoid its confounding effect.

In the past we used an F_6_ population from a full-sib advanced intercross line (FSAIL) to map MD QTL regions (QTLRs) on the chicken autosomes by individual genotyping^14^. We further mapped MD QTLRs on the chicken Z sex chromosome (GGZ) utilizing the same F_6_ population by individual genotyping, and sires from 8 elite commercial egg production lines by selective DNA pooling (SDP)^15^.

Herein we complete the study of autosomal QTLRs involved with resistance to MD, utilizing the same DNA pools from the 8 elite commercial line males used to map chromosome Z. Selective genotyping reduces the costs of QTL mapping by genotyping only individuals with extreme phenotypes^16^. SDP takes this one step further by pooling DNA from the selected individuals, and basing the linkage test on densitometric estimates of marker allele frequencies from the pools only. This can reduce genotyping costs of marker-QTL linkage determination by up to two orders of magnitudes^16–18^. As the populations used for SDP were different from the F_6_ population, one would expect partially different results (see below for more on these populations).

Assuming that individual genotyping should also reveal the true QTLR effects found by the pools, 7 of the QTLRs were also tested for confirmation of the QTLRs based on individual genotyping of all sires from the 8 lines.

## Results

### MD QTLR mapping by selective DNA pooling (SDP) in 8 pure lines

Selected DNA pools of males from 8 lines (Table S1) were used to map MD QTLRs on all autosomes. A total of 39 QTLRs were found within lines (presented in Table S2 by line, Table S3 by location). Two pairs of QTLRs within 1 Mb of one another were consolidated, both on GGA 1 (Table S3). Thus, a total of 37 QTLRs were found in 7 of the 8 lines (Table 1), with 2 of the QTLRs found in 2 lines, namely QTLR 6 in Lines WL2 and WL3, and QTLR 8 in Lines WL2 and WL4 (Table 1). The remaining 35 QTLRs were unique to a single line only. No QTLR was identified in Line RIR1, while up to 10 QTLRs were found within the other 7 lines (Table S4). The QTLRs found were distributed over 15 autosomes, with 1 to 9 QTLRs per chromosome (Table S5).

**Table 1:** Consolidated autosomal QTLRs found by pools of 8 elite chicken lines, by location. QTLR is the serial number across lines; Line(s) is the line or lines where the QTLR was identified; L. QTLR is the QTLR serial number within line (Table S2); T. mAvg is the top value in the QTLR of a moving average of -Log_10_P of a 59-markers window (this window identifies the QTLR, and from here the Drop 1 was started to both sided to set the QTLR boundaries (see detailed example in Lipkin et al. (2016)^38^); GGA is the chromosome; Start, End are the locations in bp on the GRCg6a reference of the first and last markers in the QTLR; Length is the size of the QTLR in bp; Distance are bp between the start of the QTLR and the end of the previous QTLR on the same chromosome.

QTLR	Line(s)	L. QTLR	T. mAvg	GGA	Start	End	Length	Distance
1	WL1	1	2.135	1	4,101,076	4,888,441	787,366	
2	WL2	1	2.485	1	36,636,709	37,715,459	1,078,751	31,748,268
3	WL4	1	2.467	1	50,057,031	50,343,848	286,818	12,341,572
4	WPR2	1	2.084	1	67,399,722	67,727,909	328,188	17,055,874
5	WL2	2	2.099	1	75,444,583	75,915,908	471,326	7,716,674
6	WL2, WL3	3, 1	2.485, 4.136	1	77,582,363	78,660,210	1,077,848	1,666,455
7	WL3	2	2.624	1	79,968,892	80,789,292	820,401	1,308,682
8	WL2, WL4	4, 2	2.449, 2.483	1	138,838,105	139,496,897	658,793	58,048,813
9	WL1	2	2.256	1	147,624,519	148,502,603	878,085	8,127,622
10	WL5	1	2.579	2	72,991,652	74,294,545	1,302,894	
11	WPR1	1	2.465	2	139,229,747	140,356,041	1,126,295	64,935,202
12	WPR2	2	2.049	2	142,856,002	143,682,824	826,823	2,499,961
13	WL5	2	2.170	3	32,434,477	32,826,841	392,365	
14	WPR1	2	2.021	3	36,447,453	36,704,640	257,188	3,620,612
15	WL3	3	2.475	3	101,485,607	101,806,281	320,675	64,780,967
16	WL3	4	2.225	4	10,620,348	10,874,666	254,319	
17	WL5	3	2.058	4	39,340,442	39,645,526	305,085	28,465,776
18	WL3	5	2.235	4	83,087,924	83,716,659	628,736	43,442,398
19	WL1	3	2.016	5	32,350,686	32,769,920	419,235	
20	WL2	5	2.137	7	5,471,011	5,685,200	214,190	
21	WL3	6	2.220	7	6,918,127	8,284,022	1,365,896	1,232,927
22	WL1	4	2.297	7	10,441,765	10,862,802	421,038	2,157,743
23	WL3	7	2.054	7	12,818,835	13,052,233	233,399	1,956,033
24	WL2	6	2.118	8	14,660,931	14,871,100	210,170	
25	WL3	8	2.429	8	23,451,547	23,800,610	349,064	8,580,447
26	WL1	5	2.049	9	5,947,704	6,191,375	243,672	
27	WL2	7	2.185	9	11,343,770	11,568,316	224,547	5,152,395
28	WL2	8	4.646	9	14,077,151	14,196,605	119,455	2,508,835
29	WL1	6	2.349	10	19,685,030	19,849,103	164,074	
30	WPR2	3	2.599	12	14,037,596	14,611,072	573,477	
31	WL2	9	3.058	13	4,923,733	5,369,032	445,300	
32	WL1	7	2.447	14	4,965,157	5,237,518	272,362	
33	WL5	4	2.928	14	6,948,282	7,084,042	135,761	1,710,764
34	WL1	8	2.165	14	13,961,820	14,465,586	503,767	6,877,778
35	WL1	9	2.423	17	5,151,108	5,390,165	239,058	
36	WL2	10	2.239	20	12,400,315	12,569,402	169,088	
37	WPR1	3	2.377	22	4,357,977	4,418,077	60,101	

The number of QTLRs found by SDP was nearly the same as the 38 found in the FSAIL F_6_ population^14^ (Table S6). However, the QTLRs found by the pools were much smaller than those identified with the F_6_, averaging 0.5 Mb (0.1–1.4), just above one-third of the average of 1.4 Mb (0.2–4.2) found in the F_6_ on GRCg6a.

Seven of the QTLRs found by the pools overlapped or were within 1 Mb of 8 of the QTLRs found in the F_6_^14^. Pool QTLRs 5 and 6 overlapped F_6 _QTLR 5 (Table S6). With these overlaps included, a total of 12 of the identified QTLRs (32.4%), confirmed previous reports^14,19–21^ (Table S7).

## QTLR allele substitution effect obtained by the pools

An average of 17.5% of the tested sires were selected for the phenotypic extreme tails (15.2 – 19.8%) and were used to construct the pools (Table S1). Average difference between high and low pools within a line was 50.3% daughter mortality (41.2 − 65.6%). Allele substitution effect was calculated for each marker within each line, based on these phenotypic means and selection intensity. The top 3 markers with the largest allele effect in each QTLR were used to estimate the QTLR allele effect, and the proportional contributions of the QTLR to the population and genotypic and phenotypic variation, *cG* and *cP* respectively (Table 2). Across all 7 lines with QTLRs, means of individual QTLR allele effects averaged 2.2% progeny MD mortality (1.1 − 3.7%). Individual QTLR contribution to the population phenotypic and genotypic variation (*cP* and *cG*) averaged 0.011 (0.006–0.028) and 0.087 (0.045–0.215), respectively (Table 2).

**Table 2:** QTLR absolute allele effect (|*δ*_*T*_|) found by the pools, the sum contribution of all QTLRs within line to the population phenotypic variation (*cP*), and genotypic variation (*cG*). QTLR is the QTLR serial number within a line (Table S2); GGA is the chromosome.

Line	QTLR	GGA	|d_T_|	cP	cG	Line	QTLR	GGA	|d_T_|	cP	cG
WL1	1	1	2.058	0.007	0.056	WL3	3	3	1.746	0.012	0.091
WL1	2	1	2.959	0.021	0.161	WL3	4	4	2.046	0.017	0.128
WL1	3	5	1.971	0.009	0.067	WL3	5	4	1.835	0.010	0.080
WL1	4	7	2.342	0.013	0.100	WL3	6	7	1.600	0.010	0.076
WL1	5	9	1.674	0.007	0.051	WL3	7	7	1.728	0.011	0.083
WL1	6	10	2.057	0.009	0.071	WL3	8	8	1.988	0.016	0.121
WL1	7	14	2.206	0.008	0.061	WPR1	1	2	3.658	0.028	0.215
WL1	8	14	2.272	0.012	0.092	WPR1	2	3	2.466	0.012	0.093
WL1	9	17	2.345	0.012	0.094	WPR1	3	22	3.127	0.012	0.090
WL2	1	1	2.408	0.010	0.075	WPR2	1	1	2.144	0.008	0.063
WL2	2	1	2.440	0.009	0.072	WPR2	2	2	2.026	0.007	0.055
WL2	3	1	2.998	0.015	0.115	WPR2	3	12	3.194	0.018	0.138
WL2	4	1	3.003	0.015	0.112	WL4	1	1	1.927	0.006	0.046
WL2	5	7	2.922	0.014	0.111	WL4	2	1	1.922	0.006	0.045
WL2	6	8	2.383	0.009	0.070	WL5	1	2	1.312	0.007	0.054
WL2	7	9	2.464	0.007	0.051	WL5	2	3	1.317	0.008	0.065
WL2	8	9	3.505	0.020	0.151	WL5	3	4	1.145	0.006	0.046
WL2	9	13	2.716	0.011	0.088	WL5	4	14	1.387	0.009	0.065
WL2	10	20	2.298	0.009	0.068	Average			2.194	0.011	0.087
WL3	1	1	2.491	0.025	0.190	Minimum			1.145	0.006	0.045
WL3	2	1	2.228	0.019	0.150	Maximum			3.658	0.028	0.215

Summing within line, *cP* averaged 0.066 MD mortality, from 0.012 to 0.120 (Table 3). The total contribution of the QTLRs to the genotypic variation was appreciable, averaging more than half of *cG*: 0.509, up to 0.920 in line WL3. Adding GGZ^15^, the genomic *cP* of QTLRs found by the pools averaged 0.063 (0.007–0.128). The average genomic *cG* approached half of genotypic variation: 0.481, from 0.050 in Line RIR1, to almost all variation in Line WL2 (0.984).

**Table 3:** QTLR allele effect parameters estimated by the pools. Within line total number of QTLRs and proportions of phenotypic and genetic variation (*cP* and *cG*) explained by the autosomal QTLRs, and GGZ QTLRs^15^. QTLRs is number of QTLRs in a line (Table S2for the autosomes^15^; for Z); Aut is autosomes; Z is GGZ; Avg is the average across lines; Min is minimum; Max is maximum; “-“: no QTLR was found in the line.

Line	QTLRs			cP			cG		
	Aut	Z	Sum	Aut	Z	Sum	Aut	Z	Sum
WL1	9	0	9	0.098	-	0.098	0.755	-	0.755
WL2	10	2	12	0.119	0.009	0.128	0.912	0.072	0.984
WL3	8	0	8	0.120	-	0.120	0.920	-	0.920
WPR1	3	0	3	0.052	-	0.052	0.398	-	0.398
WPR2	3	2	5	0.033	0.016	0.049	0.255	0.119	0.375
WL4	2	1	3	0.012	0.006	0.018	0.091	0.045	0.136
WL5	4	0	4	0.030	-	0.030	0.231	-	0.231
RIR1	0	1	1	-	0.007	0.007	-	0.050	0.050
Avg	4.9	0.8	5.6	0.066	0.009	0.063	0.509	0.072	0.481
Min	0	0	1	0.012	0.006	0.007	0.091	0.045	0.050
Max	10	2	12	0.120	0.016	0.128	0.920	0.119	0.984

## Association tests of QTLRs by individual genotyping

Genomic elements, namely 28 protein coding genes and one microRNA located within and around 7 of the QTLRs were identified (Table S8), and markers in them (Tables S9) were tested for association with daughter MD mortality by individual genotyping of all sires from the lines used in the SDP. This analysis was based on the assumption that – just by their QTLR location – at least some of these elements and markers are likely to have high LD with the causative element(s) in the QTLR (as QTLR mapping found marker effects by pools and not individual genotypes, it is not possible to test LD between the markers used in the SDP mapping and the markers genotyped individually).

A total of 64 informative markers were tested (Table S10, Figure S1). Numerous significant (0.05 ≥ *P* > 0.01) to highly significant (*P* ≤ 0.01) results were obtained. Runs of identical to very similar *P*-values, whether significant or not, were found within QTLRs in all lines, as we reported previously^14,15^.

Three to 20 markers in 7 of the QTLRs were tested by JMP Genomics SNP-Trait association Trend test, 18–113 tests depending on the informativity of the markers in the lines, to a total of 398 tests (Table 4). Taking one significant test as a validation^14,15^, individual genotyping confirmed the association of 5 of the 7 QTLRs tested (Table 4).

**Table 4:** Testing QTLRs by individual genotyping. QTLR serial number (Table 1). GGA is the chromosome of the QTLR; Markers is the number of markers tested in the QTLR; Tests is the total number of combined within and Across Lines tests together (Table S10); Confirmed: “+”, at least one significant test at *P* ≤ 0.05; “-“, no significant tests.

QTLR	GGA	Markers	Tests	Confirmed
2	1	4	18	+
4	1	8	53	-
17	4	7	52	+
28	9	20	113	+
31	13	3	18	-
34	14	5	36	+
35	17	17	108	+
Average		9.1	56.9	
Minimum		3	18	
Maximum		20	113	
Total		64	398	

## Allele effect of QTLR markers estimated by individual genotyping

Marker allele substitution effect was obtained by the Trend test for all markers within lines. The line contributions to the phenotypic (*cP*) and genotypic (*cG*) variations (Table S11), were based only on the significant markers. As no tested marker was significant in Lines WL3, WPR2 and WL4 (Table S10), allele effects were obtained only from the remaining 5 lines. The total contribution of the marker to the phenotypic variation averaged 0.046 in a line (0.006–0.125). A considerable contribution to the genotypic variation was found, with an average of 0.356 (0.045–0.962).

## Linkage disequilibrium among all markers on the same chromosome

LD *r*^2^ values among all possible marker pairs on the same chromosome were calculated within all lines for all informative markers. Blocks were defined as a group of markers sharing high LD (*r*^2^ ≥ 0.70) or moderate LD (0.15 ≤ *r*^2^ < 0.70). Though differing between lines, complex (e.g., Table 5) and simple (e.g., Table 6) LD blocks were found in all QTLRs examined (Table S12).

**Table 5:**
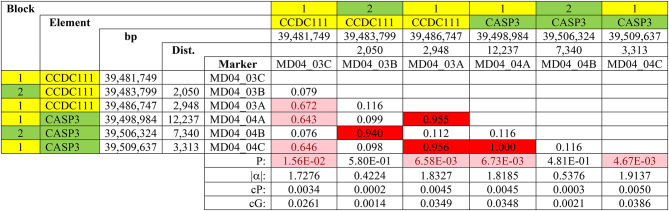
Complex LD blocks in Line WL2, QTLR 17 on GGA4. Block is a moderate to high LD block (the 2 blocks have different colors); Element is a QTLR genomic element (Table S8): different elements have different colours; bp is the location in bp on the GRCg6a genome assembly; Dist. is the distance in bp between the marker and the previous marker; Marker is the marker tested (Tables S9 and S10); LD values: red, r^2^ ≥ 0.7; pink, 0.15 ≥ r^2^ < 0.7; white, r^2^ < 0.15; P is the P-value of the Trend association test (Table S10): pink, P ≤ 0.05; white, P > 0.05; |α| is the absolute marker allele substitution effect; cP is the marker contribution to the population phenotypic variation; cG is the marker contribution to the population genotypic variation.

**Table 6:**
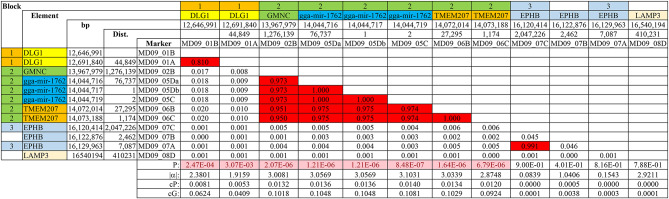
Simple and complex LD blocks in Line WL2, QTLR 28 on GGA9. Block is a high LD block (the 3 blocks have different colors); Element is the QTLR genomic element (Table S8): different elements have different colors; bp is the location in bp on the GRCg6a genome assembly; Dist. is the distance in bp between the marker and the previous marker; Marker is the marker tested (Tables S9 and S10); LD values: red, r^2^ ≥ 0.7; pink, 0.15 ≥ r^2^ < 0.7; white, r^2^ < 0.15; P is the P-value of the Trend association test (Table S10): pink, P ≤ 0.05; white, P > 0.05; |α| is the absolute marker allele substitution effect; cP is the marker contribution to the population phenotypic variation; cG is the marker contribution to the population genotypic variation.

A mix of very high and practically no LD was often found (e.g., Table 5). This mix was found even over very short distances: in Table 5, for example, Marker MD04_3B had practically no LD with Markers MD04_03C and MD04_03A located within less than 3 Kb on both sides (*r*^2^ = 0.099 and 0.098), while having a very high LD (*r*^2^ = 0.940) with MD04_04B, more than 22 Kb downstream.

Looking beyond the low LD markers, the mixed LD values formed fragmented and interdigitated high and moderate LD blocks. As seen by the colours of the markers in Table 5, the green high LD block comprised of Markers MD04_03B and MD04_04B, fragmented and surrounded by a (yellow) larger moderate to high LD block made of the remaining 4 markers tested in this QTLR. As we showed previously^15^, if markers of one block were not included in the analysis (e.g., because they were not in the marker list, were filtered out by the quality control, or were not polymorphic in this line), then one clear unambiguous block would have been identified in this region. LD blocks fitted well with *P*-values and allele effects parameters (Tables 5, 6, S12).

## Discussion

### QTLR mapping by SDP

Selective DNA pooling was applied to reduce the costs of QTL mapping by up to two orders of magnitude. DNA pools of sires selected from 8 elite Hy-Line lines with daughter tests for MD mortality were used to map MD QTLRs on the chicken autosomes. After consolidating QTLRs within 1 Mb of one another, both within and across lines, 37 QTLRs were found in 7 lines. Two of the QTLRs were found in 2 lines, while the other 35 QTLRs were unique to a single line.

No QTLR was identified in Line RIR1. This result is somewhat intriguing, as RIR1 happened to be the line with the strongest selection to the tails, i.e. the smallest proportion in the tails and consequently the highest selection intensity (*i*_*p*/2_ = 1.5 in Table S1). However, despite the strongest selection, the difference between the high and low pools and the phenotypic variation were not exceptional among the lines (Table S1). A possible explanation for the lack of QTLRs in this line could be a founder effect, but we think this is unlikely. Such an explanation would raise the question of why this effect was found in this line only, as Line RIR1 was not developed any differently than any of the other lines, 50–70 years ago. Also, note that 5 markers in QTLRs 28 and 34 were significant in this line by individual genotyping (Table S10), as were some markers in QTLRs found in the F_6 _population^14^. As this line has not been involved in other MD related QTL studies, we have no other results with which to compare, except noting that one QTLR was found in this line on chromosome Z^15^. Lastly, over-correction for the MHC could eliminate QTLRs, but this was done in this line exactly as for all other 7 lines, again raising the question of what makes this line different (see below more on the MHC correction). All in all, the best explanation for the lack of QTLR in Line RIR1 would be sampling variation and a case of false negatives.

The identified QTLRs distributed over 15 autosomes. Finding QTLRs on only some of the chromosomes aligns well with previous studies, including our own in the FSAIL F_6 _population^14^. It also accords well with the expectation that a limited number of genomic elements have a detectable effect on any trait, and those causative elements are not evenly distributed over the genome^12,22^. Finally, as always, QTLRs may be missed due to sampling variation (a false negative error).

No QTLR was found on GGA 16, the location of the chicken MHC. The assembly quality and overall coverage of chromosome 16 is not good in the GRCg6a reference genome^23^, and this could be the reason for the lack of identified QTLR. The Chicken QTLdb reports a single MD QTL on this chromosome, which came from our own report on the FSAIL F_6 _population^14^. There too, we accounted for the MHC genotype. The influence of the MHC on disease resistance is very well known in the chicken^13^. The analysis was done specifically to remove the MHC impact as this could have overwhelmed the identification of other non-MHC regions. Keeping in mind that sampling variation could cause a false positive error in the F_6_ study or a false negative here in the SDP, it nevertheless seems that the MHC correction worked well.

Seven QTLRs were tested by individual genotyping. Taking one significant test as a validation, individual genotyping confirmed the association of 5 of the 7 QTLRs tested (Table 4). Note that validation of these QTLRs is based on all sires from the lines from which samples of sires were selected to construct the selected DNA pools. Thus, this is not a completely independent replication test. Hence, the 71% confirmation cannot be taken as high. On the other hand, the number of QTLRs tested by individual genotyping is too small for a firm conclusion.

The number of QTLRs was nearly the same as the 38 found by the other arm of the same project, namely QTL mapping in an FSAIL F_6 _population^14^, suggesting the same power of the two methods. Nevertheless, the size of the QTLRs in the two studies was very different − 0.49 Mb by the pools (0.06–1.37), compared to 1.37 Mb by F_6_ on GRCg6a (0.24–4.29). Thus, the average size of QTLRs found by the pools was 36% of that identified by the F_6_, suggesting a much higher resolution of the pure lines and SDP design compared to the F_6_ of the full-sib advanced intercross. It is not trivial to compare the two studies. Too many aspects are different, with too many confounding factors. One F_6_ population on one side vs. 8 lines on the other. The F_6 _was a cross utilizing two lines, each with a very different susceptibility to MD (one line related to Line WL4 used here). It was designed for fine QTL mapping with high genetic variance in the population and map expansion over generations^21^. The eight lines are closed populations of elite stock, each intensively selected for multiple production traits, with some selection for resistance to MD^10^. All F_6_ birds were genotyped individually, while the 8 lines were tested by SDP. Thus, all we can say is that apparently the finer QTL mapping of F_6_ on one side, and the number of populations and selective DNA pooling on the other, compensated each other.

Only two within-line QTLRs overlapped with a QTLR in another line, while 35 QTLRs were line-specific (Table S3). Finding a QTLR in only one-line accords well with Smith et al. (2020)^14^, where 10 of 19 autosomes with MD QTLRs, showed QTLRs in only one family. As reported previously, many genomic elements affect the response to MD, most with relatively small effect^12^. Thus, identification of QTLRs in a single line may confirm those previous observations that indeed most QTLR effects are too small for identification with the current experimental design.

The limited overlaps of QTLRs found here with previous studies is not a surprise, given the very different designs, including different populations (e.g., the same F_6 _in Heifetz et al. (2007, 2009)^20,21 ^and Smith et al. (2020)^14 ^vs. 8 elite lines here), different genotyping methods (individual genotyping by Yonash et al. (1999)^19^, 600 K SNP array genotyping by Smith et al. (2020)^14^, SDP by Heifetz et al. (2007)^20 ^and in the present study), different marker types (microsatellites by Heifetz et al. (2007, 2009)^20,21 ^and Yonash et al. (1999)^19^, SNPs by Smith et al. (2020)^14 ^and here), and different phenotypes (e.g., age of death or survival by Heifetz et al. (2007)^20 ^and Smith et al. (2020)^14^, various MD traits and MD indexes by Yonash et al. (1999)^19^, and daughter mortality in this study).

## QTLR allele substitution effect obtained by the pools

The top 3 marker allele substitution effects were used to estimate QTLR allele effects and their contributions to the phenotypic and genotypic variation (*cP* and *cG*). These were summed within each line to estimate the variations explained by the QTLRs in that line. QTLR allele effects averaged 2.2% MD-mortality in the progeny (Table 2). Thus, the absolute effect of these individual QTLRs is appreciable - the difference between alternative homozygotes averaged 4.4% daughter mortality. Autosomal QTLRs explained an average of 6.6% and 50.9% of the phenotypic and genotypic variations within lines, which was up to 98.4% of the genetic variation (Table 3). These very high proportions could be a result of sampling variation. Alternatively, they could arise from overestimating the allele effect in the present study (for example, using only the top three markers could bias upward the estimate of the QTLR effect; possibly more markers are needed to represent the QTLR). Last, the MHC could be under-corrected due to sample variation and other technical factors. On the other hand, the results of the pools accord well with individual genotyping (below). Thus, these results are impressive, indicating the potential benefit of integrating these QTLRs in breeding programmes for MD resistance.

Vallejo et al. (1997)^24 ^and Yonash et al. (1999)^19^, both using the same resource F_2_ population and microsatellites, mapped QTLs of MD traits and MD indexes. Based on their results, individual QTL explained an average of 3.8–4.2% of the trait phenotypic variation, almost 4 times the average of 1.1% found here (Table 2). The summed phenotypic variation explained by the QTLs averaged 9.0% and 17.9%, which is 1.5 to almost 3 times the sum found here (Table 3). However, the relatively large estimates of those two studies are likely to be upwardly biased, due to their very limited power based on very sparse microsatellites compared to the high-density SNP array used here. As has been shown previously, when power is low, allele substitution effects are biased upwards^25^.

### Allele effect of QTLR markers estimated by individual genotyping

Individual genotypes of QTLR markers were used to obtain allele substitution effects. Significant markers were used to obtain contributions to the phenotypic (*cP*) and genotypic (*cG*) variations within lines. According well with the estimated allele effects based on pool genotypes, appreciable contributions were found, with means of 0.046 and 0.356 for *cP* and *cG* respectively (Table S11). These values are comparable to the *cP* and *cG* means of 0.066 and 0.509 estimated by the pools (Table 3). The pools and the individuals were each tested by very different platforms – selected DNA pools genotyped by SNP array vs. individual genotyping of all sires. The calculation methods were also very different – allele intensities of SNP microarray genotypes in the pools vs. individual genotypes. Finally, only a few of the QTLRs were tested by individual genotyping of just a few elements each. Thus, the similarity of the allele effects is impressive, mutually validating these results.

### Linkage disequilibrium among QTLR markers

LD was calculated for all possible pairs of markers on the same chromosome, and LD blocks were identified. Moderate to high LD blocks were found in all lines. Somewhat different LD patterns were found among the 8 lines on the same chromosome. The sources of these differences could partly be due to different marker informativity in the various lines (and hence different markers listed in the analyses), or result from true differences in population structure, as a consequence of different histories of each line.

A mix of very high and practically no LD values was often found between adjacent markers, even over very short distances. Skipping non-significant markers, again we found interdigitated and fragmented LD blocks (e.g., Table 5), as we reported previously^15,26^. Such a mix could result from assembly errors^27^. However, assuming few assembly errors, they would be expected to be expressed mostly as high LD among the majority of adjacent markers, and islands of singleton markers with low LD with the surrounding markers. The groups, rather than single markers, consistently showed repeating LD values, suggesting that the complex blocks are not an artefact, but a true biological phenomenon. Furthermore, the repeating of such complex blocks in 8 independent populations here, and in previous reports^15,22,26,28–30^, strengthens the claim that this is a true phenomenon. It could have genuine biological meaning through processes such as gene conversion, population bottlenecks, non-random mating and more.

In accord with our previous report, the blocks fitted well with the distribution of the *P*, *|α|*, *cP* and *cG* values, justifying their use to infer location and identify causative elements. For example, in Table 5 the mutation causing the significant test of Marker MD04_04C, is not likely near the immediate upstream 3.3 Kb non-significant Marker MD04_04B with no LD between them (*r*^2^ = 0.116), but more likely 10.7 Kb upstream, near Marker MD04_04A with similar *p*-value and complete LD between them (*r*^2^ = 1.000).

### Testing QTLRs by individual genotyping

Seven of the QTLRs were tested by individual genotyping of sires at markers within and around the QTLRs, residing in QTLR genomic elements or candidate genes to affect MD response by their location (QTLR) and reported function. Association tests, in silico investigation and LD analysis were used to assess the potential candidate elements and to narrow possible location of causative mutation. To exemplify this, detailed discussion of QTLRs 17 and 28 is presented in the following sections. The remainder of the tested QTLRs are presented in the supplementary materials.

### QTLR 17 on GGA4

QTLR 17 was found by the pools in Line WL5 (Table 1), but was significant by individual genotyping only in Line WL2 and Across Lines (Table S10). Line WL5, however, was tested individually by only 4 of the 9 markers tested in this QTLR, with one marker approaching significance. Thus, the lack of significance by individual genotyping in this line could be a matter of sampling variation or marker informativity.

In the significant WL2 Line, the above mentioned fragmented interdigitated high and moderate LD blocks were found (Table 5). The 4 significant markers created a moderate to high LD block, of which the 3 most significant were in a high LD block, surrounding a high LD block of 2 non-significant markers.

Though the 4 significant markers in Line WL2 comprised an LD block, they included 2 different genes: 2 markers from the *CCDC111* gene (PRIPOL; Primase and DNA Directed Polymerase), and 2 from *CASP3* (Caspase 3) (Tables S8 and S9). In humans, *CCDC111 *is involved in DNA damage response and adaptive response to cisplatin, a chemotherapeutic that causes reversal of replication forks in cancer cells^31^. *CASP3*was associated in human with monocytic leukaemia and colonic adenocarcinoma^32^.

Given the very high LD among these 3 markers, the similar *P*-values, and their function, both genes are candidates as a causative element. However, among the highly significant markers, only MD04_03A is an amino acid substitution, between the similar isoleucine and leucine at position 199 in the *CCDC111* gene (Table S9). The similarity between the two alternative amino acids makes this polymorphism unlikely to change the protein function. Marker MD04_04A is intronic in the *CASP3* gene, and MD04_04C is downstream of *CASP3*. Again, these locations make both unlikely to be the causative mutation.

The actual causative element(s) could be part of the *CCDC111* or *CASP3* genes, for example a regulatory mutation in the promoter, or an independent element(s) in high linkage with them. More study is needed to confirm this.

### QTLR 28 on GGA9

**Fig. 1:**
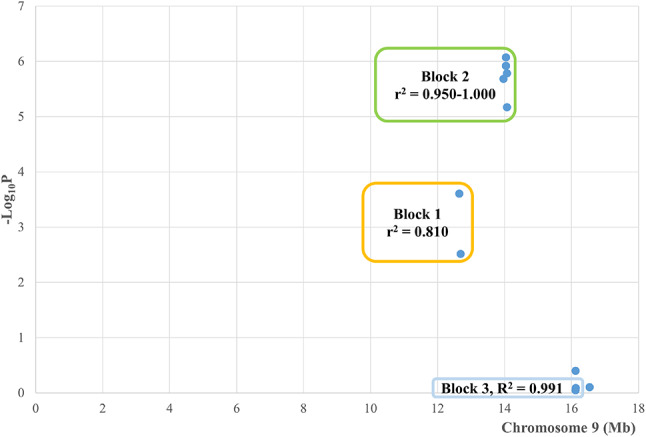
QTLR 28 and surroundings on chromosome 9 in Line WL2 (Tables 1 and 6). The x-axis is the location of the markers in Mb; the y-axis is the -Log_10_P of the marker-trait association Trend test; each blue dot represents a marker test; orange, green and blue rectangles are the high LD blocks with the same numbers and colors as the markers in Table 6.

QTLR 28 was found by the pools in Line WL2 (Table 1), and was also significant by individual genotyping, along with Lines WPR1, WL5, RIR1, and also Across Lines (Table S10). QTLR 28 was tested by 20 markers (Table 4, Figure S1), with the QTLR distributed unevenly around the markers. Inspecting marker locations and distances between them (Table S10, Fig. 1), split this QTLR into 3 regions: 2 markers in the upstream region at 12.6–12.7 Mb (Markers MD09_01B, MD09_01A), 11 markers in the central region at 14.0–14.1 Mb (Markers MD09_02A – MD09_06C), and 7 markers in the downstream region at 16.1–16.5 Mb (Markers MD09_07C - MD09_08D).

All 8 upstream and central markers tested in Line WL2 were significant, but none of the remaining 4 downstream markers were (Table 6; Fig. 1). Location, *P*-values and LD blocks accorded well with one another (Table 6). The 2 significant markers of the upstream *DLG1* gene formed one simple high LD block. Six significant markers of the central *GMNC* gene, micro-RNA gga-mir-1762 and the gene *TMEM207*, formed another simple high LD block. Finally, of the 4 downstream non-significant markers, the 2 most non-significant in the gene *EPHB* formed yet a third, but fragmented, high LD block. These results suggest two causative elements distributed in Line WL2; one in or close to the upstream gene *DLG1*, and another in the neighbourhood of the central three significant elements.

A stretch of 9 significant and 1 approaching-significance markers in Line WPR1 distributed in the central region of QTLR 28, from *GMNC* to *TMEM207* (Table S10). The first 2 upstream and the last 2 downstream markers tested in this line were not significant. Similar, albeit somewhat more complex distributions of locations, *P*-values and LD blocks (Table S12), suggest that only a central causative element of Line WL2 is distributed in this line. In Line WL5 too, a central stretch of significant markers (Table S10) formed a simple high to moderate LD block (Table S12), again suggesting a single central causative element, likely shared with Lines WL2 and WPR1.

Line RIR1 differs from the previous 3 significant lines. The 4 significant markers included 1 upstream, 1 central, and 2 downstream markers. The upper significant Marker MD09_01B had practically no LD with any other marker in this QTLR, the central significant Marker MD09_06A had very low to moderate LD with the central block of markers and the 2 downstream significant Markers MD09_08B and MD09_08C were in complete LD with one another. Thus, Line RIR1 may be displaying the upper and central causative elements found in the 3 previous significant lines, and also an additional downstream causative element.

The Across Lines significant tests distributed over the entire QTLR, mixed with non-significant markers, overall enhancing the presence of 3 causative elements in this QTLR, namely an upstream element distributed in Lines WL2 and RIR1, a central element distributed in Lines WL2, WPR1, WL5 and RIR1, and a downstream element distributed only in Line RIR1.

The upstream region includes the gene *DLG1 *(Discs Large MAGUK Scaffold Protein 1). This gene has, among others, a role in signal transduction, cell proliferation, synaptogenesis and lymphocyte activation^33^. Both markers tested in this region were non-coding.

The central region includes the gene *GMNC*, an intergenic marker, micro-RNA gga-mir-1762, and the *TMEM207* gene. *GMNC *(Geminin Coiled-Coil Domain Containing) has been predicted to affect chromatin binding activity, DNA replication, cell cycle, and cilium assembly^34^. Both markers tested in this region were non-coding. gga-mir-1762 (Gallus gallus (chicken) microRNA 1762) is where the most significant results were obtained in Lines WL2, WPR1 and WL5. Unfortunately, there is currently no known function for this miRNA. *TMEM207 *(Transmembrane Protein 207) has been associated with cancer in human^35^. This association makes it a prime causative candidate in this region, and indeed in Line RIR1 Marker MD09_06A in this gene was the only significant marker in the central region (Table S10). Two of the 3 markers tested in this gene are silent mutations (MD09_06A, MD09_06B), while MD09_06C lies in the 3’ region of this gene (Table S9).

*LAMP3 *(Lysosomal Associated Membrane Protein 3) was the only gene significant in the downstream region of QTLR 28, and only in Line RIR1 and Across lines tests. This gene is involved in the unfolded protein response (UPR) that contributes to protein degradation and cell survival during proteasomal dysfunction, autophagic process, dendritic cell function and adaptive immunity against microbial and viral infections^36^.

Two markers were significant in Line RIR1 (Table S10): Marker MD09_08B involved substitution between the nonpolar and hydrophobic amino acids glycine and proline, and Marker MD09_08B which is a silent mutation. Thus, both markers are unlikely to represent a causative mutation, although variation in a currently undefined regulatory element is possible.

### Summary

Marek’s Disease Virus imposes a heavy load on the global poultry industry. Currently, MD is controlled with limit success by vaccines and genetic breeding. Mapping QTLs affecting resistance may help the genetic improvement of birds. Here, we efficiently mapped 37 MD QTLRs by using various methods, including FSAIL, SDP, allele effect and LD. Impressive results were obtained, indicating the potential benefit of integrating these QTLRs in breeding programmes for MD resistance. The mapped QTLRs can be used directly by marker- or gene-assisted selection, or be incorporated in Genome Based Estimated Breeding Values (gEBV). Identification of the causative elements and mutations will increase the effect of the use of the mapped QTLRs. This can be achieved by fine mapping, animal models and molecular methods such as gene editing.

## Materials and methods

### Populations

All procedures carried out on the birds involved in this study were conducted in compliance with Hy-Line International Institutional Animal Care and Use Committee guidelines.

Eight of the populations described by Lipkin et al. (2023)^15^were used in the present study, namely sires from 8 elite commercial egg production lines used by Smith et al. (2020)^14^ to test autosomal QTLRs found in the F_6 _FSAIL and by Lipkin et al. (2023)^15^ to map and test QTLRs on GGZ. DNA from those same sires were used to identify autosomal QTLRs by SDP, and to test some of them.

Briefly, individual males were mated to multiple females to produce 30 half-sib female progeny per sire. Progeny were vaccinated at 1 day of age and at 7 days of age inoculated subcutaneously with MDV. Mortality was recorded from 3 to 17 weeks of age, when the experiment was terminated. At the termination of the study animals were humanely euthanized under the approval of the Hy-Line IACUC and supervision of licensed veterinarians.

Thus, the sire MD tolerance phenotype is the proportion of survivors among the daughters upon MD challenge. Data were available from 15 generations from 1995 to 2014.

MHC haplotypes for each sire were determined by an MHC-specific SNP panel as described by Fulton et al. (2016)^37^.

The 8 lines included three different egg production breeds, namely five White Leghorn lines (WL), two White Plymouth Rock lines (WPR), and one Rhode Island Red line (RIR1)^14^. Data were available for 15 generations (not the same generations in all lines). A total of 8,992 MD-phenotyped sires were genotyped for the MHC, 899–1,383 in any given line (Table S1). Based on MD daughter mortality corrected for MHC, 1,576 of these males were selected to the extreme tails, 137–225 in a line. Thus, the proportion in the high and the low pools together averaged 0.175 among the lines, 0.152–0.198 in a line. These selected sires were used to construct the high and low pools (Table S1).

### Mapping QTLRs affecting daughter MD mortality by selective DNA pooling (SDP)

Sires were phenotyped for the proportion of survivors among the daughters following MD challenge, and the mortality values were corrected for the MHC genotype as described^15^. DNA from 1,576 out of 8,992 MD-phenotyped and MHC-genotyped sires belonging to the extreme high and low tails of the phenotypic distribution was used to construct DNA pools (Table S1), the same pools used by Lipkin et al. (2023)^15^. All pools were genotyped using the Affymetrix 600 K chicken SNP array.

QTLR mapping analysis was conducted as in Lipkin et al. (2016)^38 ^and Lipkin et al. (2023)^15^. Following Lipkin et al. (2016)^38^, frequencies of SNP alleles were estimated based on raw intensities of alleles A and B, B% = B/(A + B). *P*-values of the frequency difference between tails were calculated based on empirical standard error (SE) within tails, assuming no QTL effect within tails. To account for the mix of high and low *P*-values (Figure S1), a moving average of -Log_10_P (mAvg) was then calculated. Given the marker spacing, a window of 59 SNPs (~ 100 kb) in steps of one marker was used to calculate the mAvg. As for the F_6_ (14] analysis, QTLs were then defined within lines by mAvg ≥ 2.0 (*p*≤ 0.05), and QTLR boundaries were defined by Log Drop 1^39^.

Following Lipkin et al. (2016)^38^, Smith et al. (2020)^14 ^and Lipkin et al. (2023)^15^, QTLRs within 1 Mb of one another were conservatively consolidated within and across lines.

Only autosomal markers were used. Originally, SNPs were used based on location within the Galgal4 chicken reference genome. Locations on the GRCg6a (Galgal6) reference were obtained by the Lift Genome Annotations tool (http://genome.ucsc.edu/cgi-bin/hgLiftOver) in the UCSC genome browser. A total of 544,473 SNPs from Galgal4 were also located on the autosomes in GRCg6a, and the updated locations were used in the subsequent QTL mapping procedure in all lines. To compare the present QTLRs to those found originally on Galgal4 in the F_6 _population of Smith et al. (2020)^14^, QTLRs were identified again in that population, using the same F_6_ phenotypes but with the updated GRCg6a locations.

### Allele substitution effect based on SDP genotyping

Following Darvasi and Soller (1994)^16 ^and Lipkin et al. (2018)^40^, allele effect δ, where 2δ is the phenotypic difference between alternative homozygotes, was calculated based on the estimated phenotypic difference between the two alternative homozygote groups across both tails, and the estimated frequency of the alternative alleles of the SNP in the pools, as follows:

Frequencies estimates of allele B in the High and Low pools (*F*_*BH*_ and *F*_*BL*_), based on the raw intensities of alleles A and B, were obtained as part of the QTLR mapping. *F*_*B*_, the frequency of allele B in the marker across both tails, was calculated as the average of *F*_*BH*_ and *F*_*BL*_; the allele frequency difference between the tails, was calculated as *D* = *F*_*BL*_ - *F*_*BH*_. The frequencies of the alternative homozygote genotypes BB and AA in the High tail were estimated as *BB*_*H*_ = *F*_*BH*_^2^ and *AA*_*H*_ = (1 - *F*_*BH*_)^2^, and their frequencies in the Low tail as *BB*_*L*_ = *F*_*BL*_^2^ and *AA*_*H*_ = (1 - *F*_*BH*_)^2^.

Mean daughter mortality corrected for MHC genotype was calculated within each line for the High and Low tails (*X*_*H*_ and *X*_*L*_). Weighted means of the genotype groups BB and AA across both tails were calculated as,

*BB* = (*BB*_H_*X_*H*_ + *BB*_L_*X_*L*_) / (*BB*_*H*_ + *BB*_*L*_).

and.

*AA* = (*AA*_H_*X_*H*_ + *AA*_L_*X_*L*_) / (*AA*_*H*_ + *AA*_*L*_).

The allele substitution effect was then calculated as,

*δ* = (*BB* - *AA*) / 2.

Delta is an upward estimate of the effect in the entire population, as it was obtained by the extreme tails of the population. Hence, following Lipkin et al. (1998)^17^, *δ* was corrected for the selection intensity, *i*_*p*/2_, where *i* is the line selection intensity and *p* is the proportion of the tail in the line, to obtain the population allele substitution effect,

*δ*_*T*_ = *δ/i*_*p*/2_

### QTLR allele substitution effect

Taking a conservative approach to avoid sampling fluctuations, the mean of the absolute *δ*_*T*_ values (|*δ*_*T*_|) of the top three markers having the largest |*δ*_*T*_| was used to estimate the allele effect of each QTLR. From this, following Mosig et al. (2001)^18^, the contribution of an individual QTLR to the genetic component (*VarG*) of the observed population phenotypic variation (*VarP*) was calculated as:

*VarQ* = 2*pq*δ_T_^2^ = 2*F*_*B*_(1-*F*_*B*_)*δ*_*T*_^2^

The proportional contribution of the QTLR to *VarP* was calculated as:

*cP* = *VarQ*/*VarP*.

*VarG* was calculated as,

*VarG* = *h*^2^*VarP*.

Where *h*^2 ^is the heritability, estimated as 0.13 by Wolc et al. (2013)^12^.

Then, the proportional contribution of the QTLR to *VarG* was calculated as,

*cG* = *VarQ*/*VarG*.

Finally, all QTLRs were summed within each line, to give the total proportions of phenotypic and genetic variations explained by the autosomal QTLRs in that line.

To estimate the same variations explained by all genomic QTLRs, data from Lipkin et al. (2023)^15^ were used to calculate the same parameters as for GGZ QTLRs.

### Bioinformatic analysis of QTLRs

Candidate elements, i.e., 28 protein coding genes and one miRNA (Table S8), were identified within and around the QTLRs as described by Smith et al. (2020)^14^. Sequence information obtained previously for each of the 8 lines^41^, was used to identify QTLR SNPs segregating in a line. Markers located in genomic elements within and around 7 of the QTLRs (Table S9), were identified from genome sequences aligned to the reference chicken genome for each line. Non-synonymous SNPs were preferred, but variants were also selected to cover the entire element if needed.

The QTLRs obtained were also compared to the Chicken QTL database (https://www.animalgenome.org/cgi-bin/QTLdb/GG/index) accessed during April 2023.

#### Testing QTLRs by individual genotyping

All selected SNPs were validated as described by Smith et al. (2020)^14^. All 8,992 MD-phenotyped and MHC-genotyped sires from the same 8 lines from which extreme samples of sires were used to construct the selected DNA pools, were individually genotyped at the selected markers by Kompetitive Allele Specific PCR (KASP)^42^.

Association of markers with daughters’ post-challenge mortality was tested using JMP Genomics SNP-Trait association Trend test (JMP Genomics, Version 9, SAS Institute Inc., Cary, NC, USA, 1989 − 201). When analyzing a line-marker combination (the “Within Line” test), the generation and MHC background were taken as class variables and fixed effects; when analyzing a marker combining all lines together (the “Across Lines” test), the line was added to the fixed effects.

Estimates of the allele substitution effect (*δ*) were obtained by the same JMP Genomics Trend test used for the SNP-MD association test. As the name ‘Trend’ implies, this test estimates the effect of substituting the minor, less frequent marker allele (m) by the major allele (M), from genotype mm through genotype Mm to MM (accordingly, a positive value of the effect associates the major allele with increased value and the minor allele with decreased value of the trait, and vice versa).

For each significant Within Line and Across Lines test (*P* ≤ 0.05), the proportional and total contributions of the marker to the phenotypic and genotypic variations were calculated as described above for the pools in the genome scan.

### Linkage disequilibrium (LD) among individually genotyped QTLR markers

LD *r*^2^ values between all possible marker pairs on the same chromosome were obtained within each line using the same JMP Genomics software used for the association test. As in Lipkin et al. (2023), LD blocks were defined as a group of markers having high LD (*r*^2^ ≥ 0.7), or moderate LD (0.15 ≥ r^2^< 0.70) with each other. The definition was applied even if markers with low LD appeared between the markers with high or moderate LD. This definition allowed identification of fragmented and interdigitated blocks^15,26^.

## Electronic Supplementary Material

Below is the link to the electronic supplementary material.

Supplementary Material 1

Supplementary Material 2

Supplementary Material 3

## Data Availability

Data have been submitted to the European Nucleotide Archive (ENA) at EMBL-EBI under study accession numbers PRJEB39142 (WGS).

## Abbreviations

FSAIL: full–sib advanced intercross line
KASP: Kompetitive Allele Specific PCR
LD: linkage disequilibrium
mAvg: moving average of -LogP of a marker association test
MD: Marek’s Disease
MDV: Marek’s Disease Virus
MHC: major histocompatibility complex
PFP: proportion of false positive
QTL: quantitative trait locus
QTLR: QTL region
SDP: selective DNA pooling
SNP: single nucleotide polymorphism
