# Supplementary material for: Mapping quantitative trait loci regions associated with Marek’s disease on chicken autosomes by means of selective DNA pooling
Source: Sci Rep. 2024 Dec 30;14:31896. doi: 10.1038/s41598-024-83356-w (PMC11686186; doi:10.1038/s41598-024-83356-w)
Supplement: Supplementary file 1 — Supplementary Material 1 [file 41598_2024_83356_MOESM1_ESM.pdf]

Chromosome 1

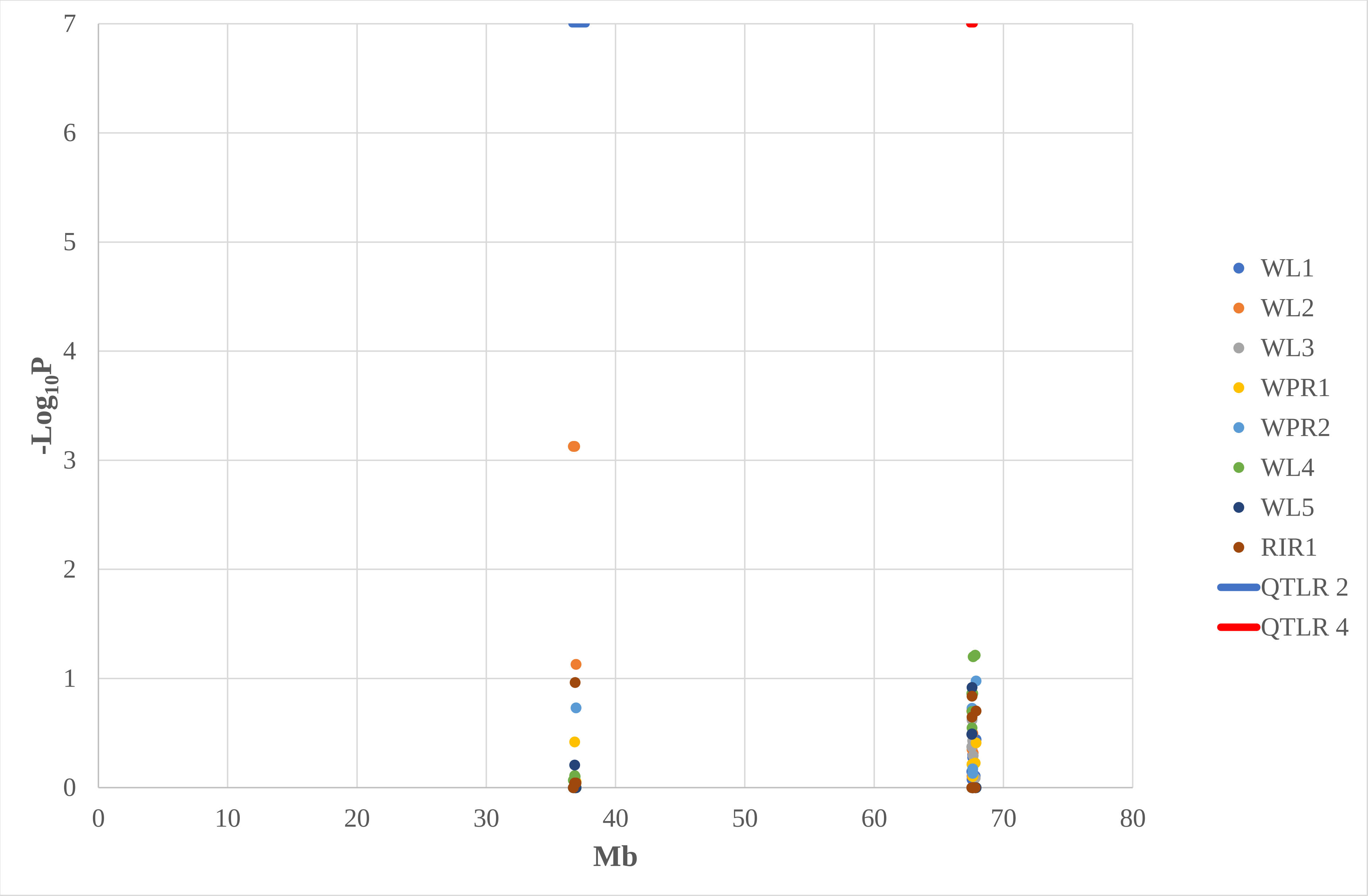

Individually genotyped QTLRs

Chromosome 4

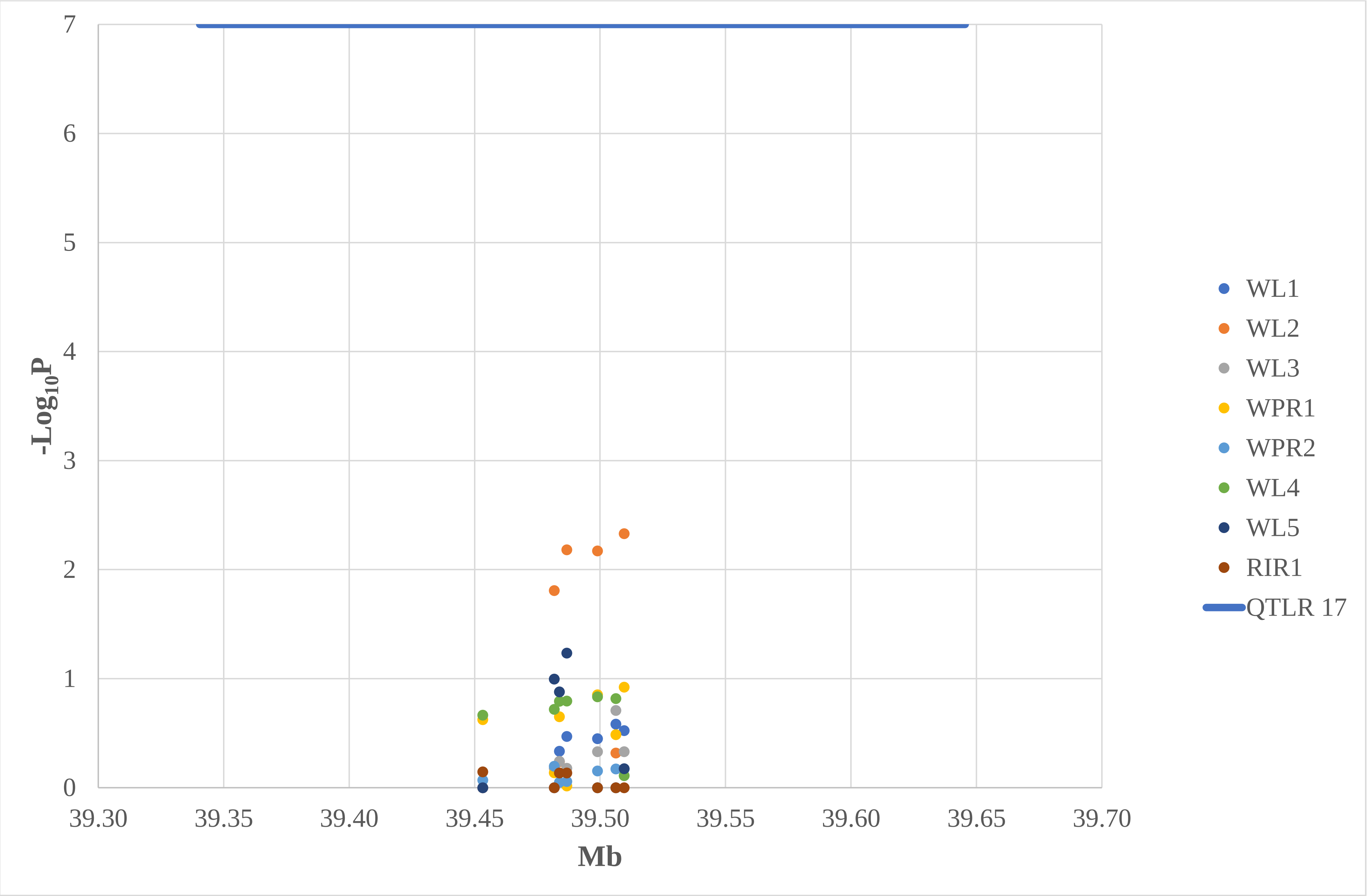

Individually genotyped QTLRs

Chromosome 9

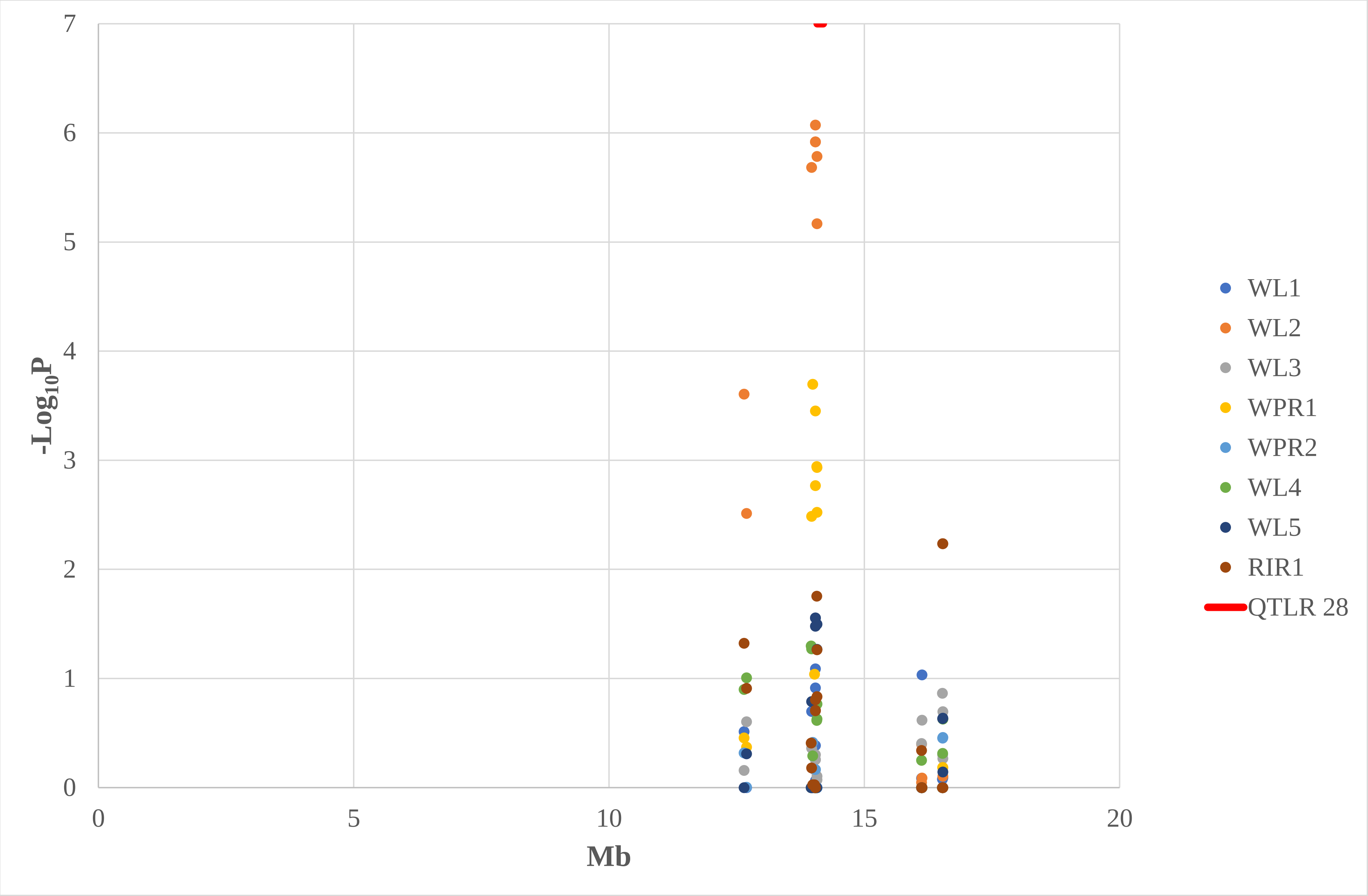

Individually genotyped QTLRs

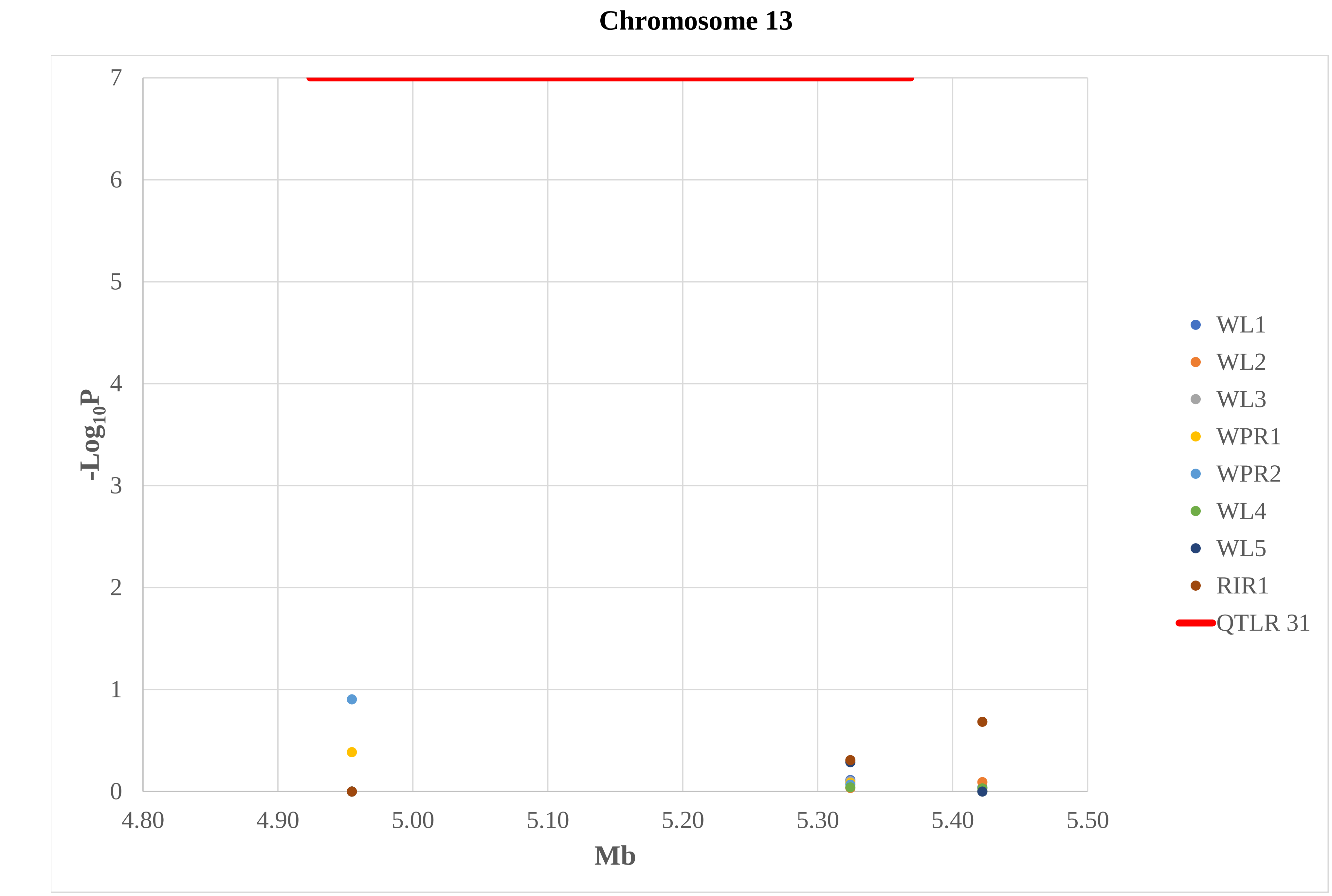

Individually genotyped QTLRs

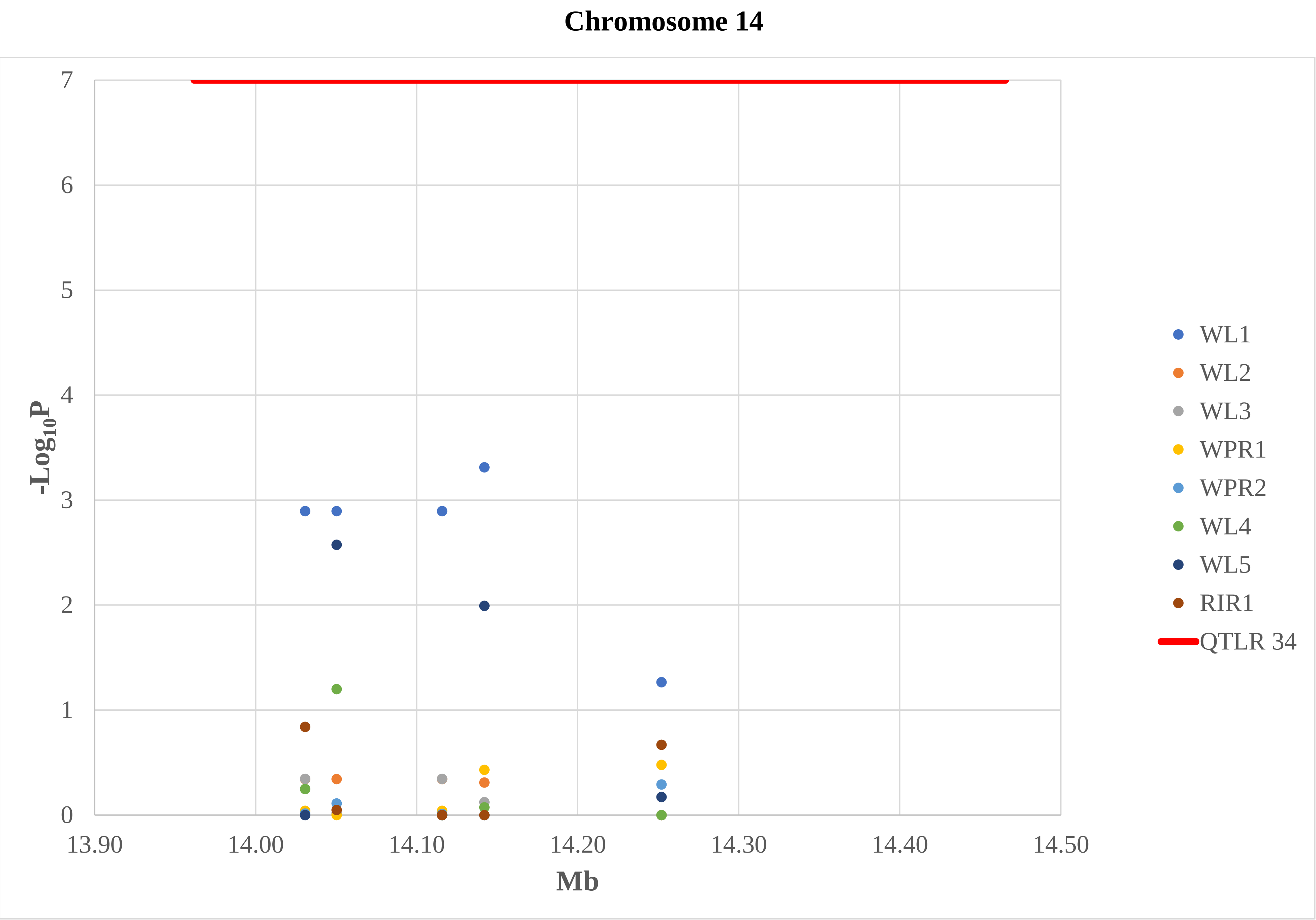

Individually genotyped QTLRs

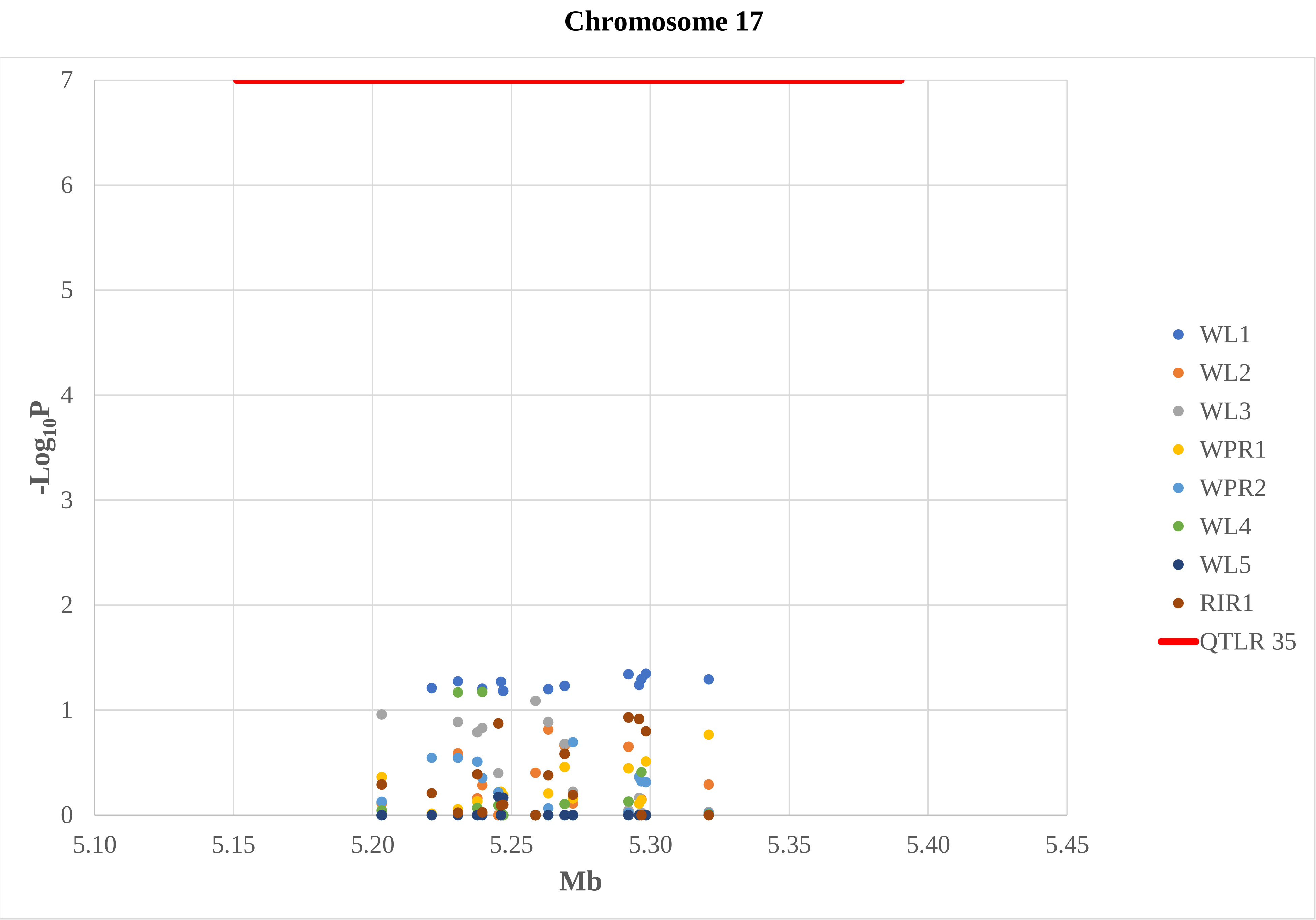

Individually genotyped QTLRs
