# Supplementary material for: Mapping quantitative trait loci regions associated with Marek’s disease on chicken autosomes by means of selective DNA pooling
Source: Sci Rep. 2024 Dec 30;14:31896. doi: 10.1038/s41598-024-83356-w (PMC11686186; doi:10.1038/s41598-024-83356-w)
Supplement: Supplementary file 2 — Supplementary Material 2 [file 41598_2024_83356_MOESM2_ESM.docx]

**Mapping quantitative trait loci regions associated with Marek’s Disease on chicken autosomes by means of selective DNA pooling**

Ehud Lipkin, Jacqueline Smith, Morris Soller, David W. Burt, Janet E. Fulton

# Supplementary materials

| **Line** | **Sires** | **Tails** | **Tail %** | ***i_p/2_*** | ***X_H_*** | ***X_L_*** | ***X_H_-X_L_*** | ***VarP*** | ***VarG*** |
| --- | --- | --- | --- | --- | --- | --- | --- | --- | --- |
| WL1 | 1,086 | 179 | 0.165 | 1.465 | 42.5 | 13.0 | 42.3 | 208.8 | 27.1 |
| WL2 | 1,109 | 211 | 0.190 | 1.395 | 46.7 | 14.0 | 46.6 | 296.1 | 38.5 |
| WL3 | 1,238 | 210 | 0.170 | 1.451 | 56.3 | 24.8 | 56.1 | 121.5 | 15.8 |
| WPR1 | 1,143 | 194 | 0.170 | 1.450 | 41.7 | 10.7 | 41.5 | 239.6 | 31.1 |
| WPR2 | 1,072 | 212 | 0.198 | 1.374 | 60.1 | 28.8 | 59.9 | 281.9 | 36.6 |
| WL4 | 1,062 | 207 | 0.195 | 1.382 | 41.4 | 14.8 | 41.2 | 307.1 | 39.9 |
| WL5 | 1,383 | 225 | 0.163 | 1.471 | 65.8 | 35.9 | 65.6 | 102.2 | 13.3 |
| RIR1 | 899 | 137 | 0.152 | 1.503 | 49.4 | 17.5 | 49.3 | 266.2 | 34.6 |
| Avg | 1,124 | 197 | 0.175 | 1.436 | 50.5 | 19.9 | 50.3 | 227.9 | 29.6 |
| Min | 899 | 137 | 0.152 | 1.374 | 41.4 | 10.7 | 41.2 | 102.2 | 13.3 |
| Max | 1,383 | 225 | 0.198 | 1.503 | 65.8 | 35.9 | 65.6 | 307.1 | 39.9 |

**Table S1.** Populations and selected pools within lines. Sires is the total number of MD-phenotyped and MHC-genotyped sires; Tails the is total number of sires selected for the extreme tails of MD daughters’ mortality corrected for MHC genotype; Tail % is the proportion of sires selected to the tails and used to construct the pools; *i_p_*_/2_: *i* is the selection intensity; *p* is the proportion of the tails in the line; *X_H_*, *X_L_* are the mean daughter mortality of the High and Low pools; *X_H_*-*X_L_* is the phenotypic difference between the means of the high and low pools; *VarP* is the population phenotypic variation; *VarG* is the genotypic variation.

| **Line** | **QTLR** | **GGA** | **Start** | **End** | **Length** | **Distance** |
| --- | --- | --- | --- | --- | --- | --- |
| WL1 | 1 | 1 | 4,101,076 | 4,888,441 | 787,366 |  |
| WL1 | 2 | 1 | 147,624,519 | 148,502,603 | 878,085 | 142,736,078 |
| WL1 | 3 | 5 | 32,350,686 | 32,769,920 | 419,235 |  |
| WL1 | 4 | 7 | 10,441,765 | 10,862,802 | 421,038 |  |
| WL1 | 5 | 9 | 5,947,704 | 6,191,375 | 243,672 |  |
| WL1 | 6 | 10 | 19,685,030 | 19,849,103 | 164,074 |  |
| WL1 | 7 | 14 | 4,965,157 | 5,237,518 | 272,362 |  |
| WL1 | 8 | 14 | 13,961,820 | 14,465,586 | 503,767 | 8,724,302 |
| WL1 | 9 | 17 | 5,151,108 | 5,390,165 | 239,058 |  |
| WL2 | 1 | 1 | 36,636,709 | 37,715,459 | 1,078,751 |  |
| WL2 | 2 | 1 | 75,444,583 | 75,915,908 | 471,326 | 37,729,124 |
| WL2 | 3 | 1 | 77,582,363 | 77,916,326 | 333,964 | 1,666,455 |
| WL2 | 4 | 1 | 138,838,105 | 139,496,897 | 658,793 | 60,921,779 |
| WL2 | 5 | 7 | 5,471,011 | 5,685,200 | 214,190 |  |
| WL2 | 6 | 8 | 14,660,931 | 14,871,100 | 210,170 |  |
| WL2 | 7 | 9 | 11,343,770 | 11,568,316 | 224,547 |  |
| WL2 | 8 | 9 | 14,077,151 | 14,196,605 | 119,455 | 2,508,835 |
| WL2 | 9 | 13 | 4,923,733 | 5,369,032 | 445,300 |  |
| WL2 | 10 | 20 | 12,400,315 | 12,569,402 | 169,088 |  |
| WL3 | 1 | 1 | 78,369,871 | 78,660,210 | 290,340 |  |
| WL3 | 2 | 1 | 79,968,892 | 80,789,292 | 820,401 | 1,308,682 |
| WL3 | 3 | 3 | 101,485,607 | 101,806,281 | 320,675 |  |
| WL3 | 4 | 4 | 10,620,348 | 10,874,666 | 254,319 |  |
| WL3 | 5 | 4 | 83,087,924 | 83,716,659 | 628,736 | 72,213,258 |
| WL3 | 6 | 7 | 6,918,127 | 8,284,022 | 1,365,896 |  |
| WL3 | 7 | 7 | 12,818,835 | 13,052,233 | 233,399 | 4,534,813 |
| WL3 | 8 | 8 | 23,451,547 | 23,800,610 | 349,064 |  |
| WPR1 | 1 | 2 | 139,229,747 | 140,356,041 | 1,126,295 |  |
| WPR1 | 2 | 3 | 36,447,453 | 36,704,640 | 257,188 |  |
| WPR1 | 3 | 22 | 4,357,977 | 4,418,077 | 60,101 |  |
| WPR2 | 1 | 1 | 67,399,722 | 67,727,909 | 328,188 |  |
| WPR2 | 2 | 2 | 142,856,002 | 143,682,824 | 826,823 |  |
| WPR2 | 3 | 12 | 14,037,596 | 14,611,072 | 573,477 |  |
| WL4 | 1 | 1 | 50,057,031 | 50,343,848 | 286,818 |  |
| WL4 | 2 | 1 | 139,053,697 | 139,441,904 | 388,208 | 88,709,849 |
| WL5 | 1 | 2 | 72,991,652 | 74,294,545 | 1,302,894 |  |
| WL5 | 2 | 3 | 32,434,477 | 32,826,841 | 392,365 |  |
| WL5 | 3 | 4 | 39,340,442 | 39,645,526 | 305,085 |  |
| WL5 | 4 | 14 | 6,948,282 | 7,084,042 | 135,761 |  |

**Table S2.** QTLRs found on the autosomes by pools of 8 elite chicken lines, by line. QTLR is the QTLR serial number within line; GGA is the chromosome; Start, End are the bp location on the GRCg6a reference of the first and last markers in the QTLR; Length is the size of the QTLR in bp; Distance are bp between the start of the QTLR and the end of the previous QTLR on the same chromosome.

| **Line** | **QTLR** | **GGA** | **Start** | **End** | **Length** | **Distance** |
| --- | --- | --- | --- | --- | --- | --- |
| WL1 | 1 | 1 | 4,101,076 | 4,888,441 | 787,366 |  |
| WL2 | 1 | 1 | 36,636,709 | 37,715,459 | 1,078,751 | 31,748,268 |
| WL4 | 1 | 1 | 50,057,031 | 50,343,848 | 286,818 | 12,341,572 |
| WPR2 | 1 | 1 | 67,399,722 | 67,727,909 | 328,188 | 17,055,874 |
| WL2 | 2 | 1 | 75,444,583 | 75,915,908 | 471,326 | 7,716,674 |
| WL2 | 3 | 1 | 77,582,363 | 77,916,326 | 333,964 | 1,666,455 |
| WL3 | 1 | 1 | 78,369,871 | 78,660,210 | 290,340 | 453,545 |
| WL3 | 2 | 1 | 79,968,892 | 80,789,292 | 820,401 | 1,308,682 |
| WL2 | 4 | 1 | 138,838,105 | 139,496,897 | 658,793 | 58,048,813 |
| WL4 | 2 | 1 | 139,053,697 | 139,441,904 | 388,208 | -443,200 |
| WL1 | 2 | 1 | 147,624,519 | 148,502,603 | 878,085 | 8,182,615 |
| WL5 | 1 | 2 | 72,991,652 | 74,294,545 | 1,302,894 |  |
| WPR1 | 1 | 2 | 139,229,747 | 140,356,041 | 1,126,295 | 64,935,202 |
| WPR2 | 2 | 2 | 142,856,002 | 143,682,824 | 826,823 | 2,499,961 |
| WL5 | 2 | 3 | 32,434,477 | 32,826,841 | 392,365 |  |
| WPR1 | 2 | 3 | 36,447,453 | 36,704,640 | 257,188 | 3,620,612 |
| WL3 | 3 | 3 | 101,485,607 | 101,806,281 | 320,675 | 64,780,967 |
| WL3 | 4 | 4 | 10,620,348 | 10,874,666 | 254,319 |  |
| WL5 | 3 | 4 | 39,340,442 | 39,645,526 | 305,085 | 28,465,776 |
| WL3 | 5 | 4 | 83,087,924 | 83,716,659 | 628,736 | 43,442,398 |
| WL1 | 3 | 5 | 32,350,686 | 32,769,920 | 419,235 |  |
| WL2 | 5 | 7 | 5,471,011 | 5,685,200 | 214,190 |  |
| WL3 | 6 | 7 | 6,918,127 | 8,284,022 | 1,365,896 | 1,232,927 |
| WL1 | 4 | 7 | 10,441,765 | 10,862,802 | 421,038 | 2,157,743 |
| WL3 | 7 | 7 | 12,818,835 | 13,052,233 | 233,399 | 1,956,033 |
| WL2 | 6 | 8 | 14,660,931 | 14,871,100 | 210,170 |  |
| WL3 | 8 | 8 | 23,451,547 | 23,800,610 | 349,064 | 8,580,447 |
| WL1 | 5 | 9 | 5,947,704 | 6,191,375 | 243,672 |  |
| WL2 | 7 | 9 | 11,343,770 | 11,568,316 | 224,547 | 5,152,395 |
| WL2 | 8 | 9 | 14,077,151 | 14,196,605 | 119,455 | 2,508,835 |
| WL1 | 6 | 10 | 19,685,030 | 19,849,103 | 164,074 |  |
| WPR2 | 3 | 12 | 14,037,596 | 14,611,072 | 573,477 |  |
| WL2 | 9 | 13 | 4,923,733 | 5,369,032 | 445,300 |  |
| WL1 | 7 | 14 | 4,965,157 | 5,237,518 | 272,362 |  |
| WL5 | 4 | 14 | 6,948,282 | 7,084,042 | 135,761 | 1,710,764 |
| WL1 | 8 | 14 | 13,961,820 | 14,465,586 | 503,767 | 6,877,778 |
| WL1 | 9 | 17 | 5,151,108 | 5,390,165 | 239,058 |  |
| WL2 | 10 | 20 | 12,400,315 | 12,569,402 | 169,088 |  |
| WPR1 | 3 | 22 | 4,357,977 | 4,418,077 | 60,101 |  |

**Table S3.** QTLRs found on the autosomes by pools of 8 elite chicken lines, by location. QTLR is the QTLR serial number within line (Table S2); GGA is the chromosome; Start, End are the bp location on the GRCg6a reference of the first and last markers in the QTLR; Length is the size of the QTLR in bp; Distance are the bp between the start of the QTLR and the end of the previous QTLR on the same chromosome: colored, overlap of QTLRs (the QTLR start before the end of the previous one).

| **Line** | **QTLRs** |
| --- | --- |
| WL1 | 9 |
| WL2 | 10 |
| WL3 | 8 |
| WPR1 | 3 |
| WPR2 | 3 |
| WL4 | 2 |
| WL5 | 4 |
| RIR1 | 0 |
| Sum | 39 |
| Average | 4.9 |
| Minimum | 0 |
| Maximum | 10 |

**Table S4.** Number of QTLRs found, by line. QTLRs is the number of QTLRs found in the line.

| **GGA** | **Line's**  **QTLRs** | **Consolidated**  **QTLRs** |
| --- | --- | --- |
| 1 | 11 | 9 |
| 2 | 3 | 3 |
| 3 | 3 | 3 |
| 4 | 3 | 3 |
| 5 | 1 | 1 |
| 7 | 4 | 4 |
| 8 | 2 | 2 |
| 9 | 3 | 3 |
| 10 | 1 | 1 |
| 12 | 1 | 1 |
| 13 | 1 | 1 |
| 14 | 3 | 3 |
| 17 | 1 | 1 |
| 20 | 1 | 1 |
| 22 | 1 | 1 |
| Chromosomes | 15 | 15 |
| QTLRs | 39 | 37 |
| Average | 2.6 | 2.5 |
| Minimum | 1 | 1 |
| Maximum | 11 | 9 |

**Table S5.** Number of QTLRs found, by chromosome. GGA is the chromosome; Line's QTLRs is the number of QTLRs found within lines (Table S2); Consolidated QTLRs is the number of consolidated QTLRs across lines (Table 1); Chromosomes is the number of chromosomes harboring QTLRs; QTLRs is the total number of QTLRs.

| **QTLR** | **GGA** | **Start** | **End** | **QTLR** | **GGA** | **Start** | **End** |
| --- | --- | --- | --- | --- | --- | --- | --- |
| 1 | 1 | 9,510,148 | 9,902,036 | 20 | 5 | 8,388,371 | 8,967,466 |
| 2 | 1 | 13,994,599 | 14,950,768 | 21 | 5 | 19,753,005 | 20,610,009 |
| 3 | 1 | 52,166,588 | 52,643,244 | 22 | 6 | 3,323,132 | 3,946,659 |
| 4 | 1 | 71,892,917 | 73,277,481 | 23 | 6 | 30,954,349 | 31,233,344 |
| 5 | 1 | 75,513,671 | 79,029,197 | 24 | 6 | 32,440,880 | 32,888,648 |
| 6 | 1 | 93,533,567 | 93,853,587 | 25 | 7 | 13,563,779 | 16,986,311 |
| 7 | 1 | 103,738,415 | 106,416,920 | 26 | 10 | 1,025,523 | 2,668,959 |
| 8 | 1 | 111,372,866 | 112,400,685 | 27 | 11 | 7,912,510 | 8,959,749 |
| 9 | 1 | 171,680,812 | 174,306,953 | 28 | 12 | 9,414,714 | 9,845,036 |
| 10 | 1 | 176,474,702 | 177,748,402 | 29 | 13 | 11,756,937 | 13,566,822 |
| 11 | 1 | 196,152,404 | 196,750,875 | 30 | 14 | 8,499,374 | 9,745,708 |
| 12 | 2 | 48,621 | 959,053 | 31 | 14 | 13,542,085 | 15,384,231 |
| 13 | 2 | 45,786,534 | 46,247,754 | 32 | 16 | 1,852,095 | 2,669,032 |
| 14 | 2 | 105,791,822 | 109,334,178 | 33 | 17 | 3,808,082 | 5,932,858 |
| 15 | 2 | 125,532,963 | 127,219,187 | 34 | 18 | 3,221,049 | 4,118,252 |
| 16 | 2 | 139,198,404 | 140,160,087 | 35 | 24 | 4,160,414 | 5,498,172 |
| 17 | 3 | 108,593,746 | 109,643,999 | 36 | 26 | 4,438,584 | 5,002,302 |
| 18 | 4 | 8,328,709 | 11,309,259 | 37 | 27 | 3,930,559 | 4,689,821 |
| 19 | 4 | 84,829,085 | 89,057,374 | 38 | 28 | 1,447,725 | 1,687,264 |

**Table S6.** QTLRs found in the FSAIL F6 population [14], updated to the GRCg6a genome assembly. QTLR is the QTLR serial number [14]; GGA is the chromosome; Start, End are the bp location on the GRCg6a reference of the first and last markers in the QTLR.

| **QTLR** | **GGA** | **Start** | **End** | **F_6_** | | | **Chicken QTLdb** | | |
| --- | --- | --- | --- | --- | --- | --- | --- | --- | --- |
|  |  |  |  | **QTLR** | **Start** | **End** | **Ref** | **Start** | **End** |
| 1 | 1 | 4,101,076 | 4,888,441 |  |  |  |  |  |  |
| 2 | 1 | 36,636,709 | 37,715,459 |  |  |  |  |  |  |
| 3 | 1 | 50,057,031 | 50,343,848 |  |  |  | **Heifetz 2007** | **40.2** | **52.1** |
| 4 | 1 | 67,399,722 | 67,727,909 |  |  |  |  |  |  |
| 5 | 1 | 75,444,583 | 75,915,908 | **5** | **75,513,671** | **79,029,197** | *Heifetz 2007* | *73.9* | *75.0* |
| 6 | 1 | 77,582,363 | 78,660,210 |  |  |  |  |  |  |
| 7 | 1 | 79,968,892 | 80,789,292 | *5* | *75,513,671* | *79,029,197* |  |  |  |
| 8 | 1 | 138,838,105 | 139,496,897 |  |  |  |  |  |  |
| 9 | 1 | 147,624,519 | 148,502,603 |  |  |  |  |  |  |
| 10 | 2 | 72,991,652 | 74,294,545 |  |  |  |  |  |  |
| 11 | 2 | 139,229,747 | 140,356,041 | **16** | **139,198,404** | **140,160,087** |  |  |  |
| 12 | 2 | 142,856,002 | 143,682,824 |  |  |  |  |  |  |
| 13 | 3 | 32,434,477 | 32,826,841 |  |  |  |  |  |  |
| 14 | 3 | 36,447,453 | 36,704,640 |  |  |  |  |  |  |
| 15 | 3 | 101,485,607 | 101,806,281 |  |  |  |  |  |  |
| 16 | 4 | 10,620,348 | 10,874,666 | **18** | **8,328,709** | **11,309,259** |  |  |  |
| 17 | 4 | 39,340,442 | 39,645,526 |  |  |  | **Yonash 1999** | **25.6** | **40.1** |
| 18 | 4 | 83,087,924 | 83,716,659 |  |  |  | *Yonash 1999* | *40.1* | *51.6* |
| 19 | 5 | 32,350,686 | 32,769,920 |  |  |  |  |  |  |
| 20 | 7 | 5,471,011 | 5,685,200 |  |  |  |  |  |  |
| 21 | 7 | 6,918,127 | 8,284,022 |  |  |  |  |  |  |
| 22 | 7 | 10,441,765 | 10,862,802 |  |  |  |  |  |  |
| 23 | 7 | 12,818,835 | 13,052,233 | *25* | *13,563,779* | *16,986,311* |  |  |  |
| 24 | 8 | 14,660,931 | 14,871,100 |  |  |  |  |  |  |
| 25 | 8 | 23,451,547 | 23,800,610 |  |  |  | **Heifetz 2007** | **8.6** | **20.8** |
| 26 | 9 | 5,947,704 | 6,191,375 |  |  |  | *Heifetz 2007* | *6.3-6.9*^a^ | *7.4-7.6*^a^ |
| 27 | 9 | 11,343,770 | 11,568,316 |  |  |  |  |  |  |
| 28 | 9 | 14,077,151 | 14,196,605 |  |  |  |  |  |  |
| 29 | 10 | 19,685,030 | 19,849,103 |  |  |  |  |  |  |
| 30 | 12 | 14,037,596 | 14,611,072 |  |  |  |  |  |  |
| 31 | 13 | 4,923,733 | 5,369,032 |  |  |  |  |  |  |
| 32 | 14 | 4,965,157 | 5,237,518 |  |  |  |  |  |  |
| 33 | 14 | 6,948,282 | 7,084,042 |  |  |  |  |  |  |
| 34 | 14 | 13,961,820 | 14,465,586 | **31** | **13,542,085** | **15,384,231** |  |  |  |
| 35 | 17 | 5,151,108 | 5,390,165 | **33** | **3,808,082** | **5,932,858** |  |  |  |
| 36 | 20 | 12,400,315 | 12,569,402 |  |  |  |  |  |  |
| 37 | 22 | 4,357,977 | 4,418,077 |  |  |  |  |  |  |

^a.^ From several QTLRs.

**Table S7.** Comparison between QTLRs found here and in previous reports. QTLR is the QTLR serial number (Table 1); GGA is the chromosome; Start, End are the bp location on the GRCg6a reference of the first and last markers in the QTLR; F_6_ are QTLRs mapped in the F_6_ population (Table S5) [14]; QTLR under F_6_ is the F_6_ QTLR serial number (Table S6); Start, End under F_6_ are the bp location on the GRCg6a reference of the first and last markers in the F_6_ QTLR (Table S6); Chicken QTLdb if the ChickenQTLdb: [https://www.animalgenome.org/cgi-bin/QTLdb/GG/index;](https://www.animalgenome.org/cgi-bin/QTLdb/GG/index) Ref are references Yonash et al. (1999) [19], and Heifetz et al (2007, 2009) [20, 21]. Start, End under Ref are the bp location on the GRCg6a reference of the first and last markers in (Table S6); Under F_6_ and Ref: Bolded - overlap between QTLR mapped by the pools and a previous report; Italic - QTLR mapped by the pools and a previous report are within 1 Mb of each other.

| **GGA** | **QTLR** | **Element** | **Description** | **1^st^ marker** |
| --- | --- | --- | --- | --- |
| 1 | 2 | TRHDE | Thyrotropin Releasing Hormone Degrading Enzyme | 36,735,741 |
| 1 | 4 | RASSF8 | Ras Association Domain Family Member 8 | 67,554,921 |
| 1 | 4 | SSPN | K-Ras Oncogene-Associated Protein | 67,629,704 |
| 1 | 4 | ITPR2 | Inositol 1,4,5-Trisphosphate Receptor Type 2 | 67,659,080 |
| 4 | 17 | ACSL1 | Acyl-CoA Synthetase Long-Chain Family Member 1 | 39,453,244 |
| 4 | 17 | CCDC111 | Primase And DNA Directed Polymerase | 39,481,749 |
| 4 | 17 | CASP3 | Caspase 3 | 39,498,984 |
| 9 | 28 | DLG1 | Discs Large MAGUK Scaffold Protein 1 | 12,646,991 |
| 9 | 28 | GMNC | Geminin Coiled-Coil Domain Containing | 13,960,302 |
| 9 | 28 | Intergenic |  | 13,991,344 |
| 9 | 28 | IL1RAP | Interleukin 1 Receptor Accessory Protein | 14,024,326 |
| 9 | 28 | gga-mir-1762 | microRNA 1762 ( Gallus gallus (chicken) ) | 14,044,716 |
| 9 | 28 | TMEM207 | Transmembrane Protein 207 | 14,069,448 |
| 9 | 28 | EPHB | EPH Receptor B2 | 16,120,414 |
| 9 | 28 | LAMP3 | Lysosomal Associated Membrane Protein 3 | 16,528,620 |
| 13 | 31 | SLIT3 | Slit Guidance Ligand 3 | 4,954,745 |
| 14 | 34 | HN1L | Hematological And Neurological Expressed 1 Like | 14,030,671 |
| 14 | 34 | CRAMP1 | Cramped Chromatin Regulator Homolog 1 | 14,050,330 |
| 14 | 34 | TMEM204 | Transmembrane Protein 204 | 14,115,781 |
| 14 | 34 | ATP6V0C | ATPase H+ Transporting V0 Subunit C | 14,252,012 |
| 17 | 35 | FAM102A | Family With Sequence Similarity 102 Member A | 5,203,358 |
| 17 | 35 | DPM2 | Dolichyl-Phosphate Mannosyltransferase Subunit 2, Regulatory | 5,237,745 |
| 17 | 35 | ST6GALNAC6 | ST6 N-Acetylgalactosaminide Alpha-2,6-Sialyltransferase 6 | 5,245,311 |
| 17 | 35 | ENG | Endoglin | 5,258,665 |
| 17 | 35 | CDK10 | Cyclin Dependent Kinase 10 | 5,269,149 |
| 17 | 35 | CDK11 | Cyclin Dependent Kinase 11B | 5,272,121 |
| 17 | 35 | SH2D3C | SH2 Domain Containing 3C | 5,292,163 |
| 17 | 35 | TOR2A | Torsin Family 2 Member A | 5,296,779 |
| 17 | 35 | URM1 | Ubiquitin Related Modifier 1 | 5,321,060 |

**Table S8.** QTLR elements tested for association with daughter MD mortality. GGA is the chromosome; QTLR is the QTLR serial number (Table 1); 1^st^ Marker is the location of the first marker in the element on the GRCg6a genome assembly (Table S9).

| **GGA** | **QTLR** | **Element** | **SNP** | **bp** | **Location/alleles** | **AA** |
| --- | --- | --- | --- | --- | --- | --- |
| 1 | 2 | TRHDE | MD01_02A | 36,735,741 | Upstream |  |
| 1 | 2 | TRHDE | MD01_02B | 36,839,853 | 1092A>G | Thr364 |
| 1 | 2 | TRHDE | MD01_02C | 36,879,328 | 1366G>A | Ala456Thr |
| 1 | 2 | TRHDE | MD01_02D | 36,951,385 | 2541T>A | Thr847 |
| 1 | 4 | RASSF8 | MD01_03A | 67,554,921 | Upstream |  |
| 1 | 4 | RASSF8 | MD01_03B | 67,564,586 | 187G>Aa | Ala163b |
| 1 | 4 | RASSF8 | MD01_03C | 67,569,296 | Downstream |  |
| 1 | 4 | SSPN | MD01_04A | 67,612,246 | 23T>C | Val8Ala |
| 1 | 4 | SSPN | MD01_04B | 67,629,704 | 500C>A | Ser167Tyr |
| 1 | 4 | SSPN | MD01_04C | 67,629,779 | 575G>C | Ser192Thr |
| 1 | 4 | ITPR2 | MD01_05C | 67,659,080 | Downstream |  |
| 1 | 4 | ITPR2 | MD01_05B | 67,810,544 | 3400A>G | Ser1134Gly |
| 1 | 4 | ITPR2 | MD01_05A | 67,882,133 | 399A>G | Thr133 |
| 4 | 17 | ACSL1 | MD04_02A | 39,453,244 | 675C>T | Ile225 |
| 4 | 17 | CCDC111 | MD04_03C | 39,481,749 | 3' |  |
| 4 | 17 | CCDC111 | MD04_03B | 39,483,799 | 1065G>A | Glu355 |
| 4 | 17 | CCDC111 | MD04_03A | 39,486,747 | 595A>T | Ile199Leu |
| 4 | 17 | CASP3 | MD04_04A | 39,498,984 | Intron |  |
| 4 | 17 | CASP3 | MD04_04B | 39,506,324 | 480G>A | Ala160 |
| 4 | 17 | CASP3 | MD04_04C | 39,509,637 | Downstream |  |
| 9 | 28 | DLG1 | MD09_01B | 12,646,991 | Intron |  |
| 9 | 28 | DLG1 | MD09_01A | 12,691,840 |  |  |
| 9 | 28 | GMNC | MD09_02A | 13,960,302 | Upstream |  |
| 9 | 28 | GMNC | MD09_02B | 13,967,979 | Downstream |  |
| 9 | 28 | intergenic | MD09_03A | 13,991,344 | Intergenic |  |
| 9 | 28 | IL1RAP | MD09_04A | 14,024,326 | 501C>T | Pro167 |
| 9 | 28 | gga-mir-1762 | MD09_05Da | 14,044,716 |  |  |
| 9 | 28 | gga-mir-1762 | MD09_05Db | 14,044,717 |  |  |
| 9 | 28 | gga-mir-1762 | MD09_05C | 14,044,719 |  |  |
| 9 | 28 | gga-mir-1762 | MD09_05B | 14,044,741 | Non coding transcript exon variant | |
| 9 | 28 | gga-mir-1762 | MD09_05A | 14,044,752 |  |  |
| 9 | 28 | TMEM207 | MD09_06A | 14,069,448 | 48C>T | Ala16 |
| 9 | 28 | TMEM207 | MD09_06B | 14,072,014 | 184C>T | Leu62 |
| 9 | 28 | TMEM207 | MD09_06C | 14,073,188 | 3' |  |
| 9 | 28 | EPHB | MD09_07C | 16,120,414 | 2772G>A | Leu924 |
| 9 | 28 | EPHB | MD09_07B | 16,122,876 |  |  |
| 9 | 28 | EPHB | MD09_07A | 16,129,963 | 615A>G | Glu205 |
| 9 | 28 | LAMP3 | MD09_08A | 16,528,620 | Intron |  |
| 9 | 28 | LAMP3 | MD09_08B | 16,532,498 | 182A>C | Gln61Pro |
| 9 | 28 | LAMP3 | MD09_08C | 16,537,751 | 1125T>C | Asp375 |
| 9 | 28 | LAMP3 | MD09_08D | 16,540,194 | 1275T>C | Arg425 |
| 13 | 31 | SLIT3 | MD13_03A | 4,954,745 |  |  |
| 13 | 31 | SLIT3 | MD13_03B | 5,324,164 | 1728G>A | Ser576 |
| 13 | 31 | SLIT3 | MD13_03C | 5,421,966 |  |  |
| 14 | 34 | HN1L | MD14_01A | 14,030,671 | 237A>G | Gln79 |
| 14 | 34 | CRAMP1 | MD14_02A | 14,050,330 | Intron |  |
| 14 | 34 | TMEM204 | MD14_03B | 14,115,781 | Intron |  |
| 14 | 34 | TMEM204 | MD14_03A | 14,142,117 | 5' |  |
| 14 | 34 | ATP6V0C | MD14_04A | 14,252,012 | Intron |  |
| 17 | 35 | FAM102A | MD17_01A | 5,203,358 | 5' |  |
| 17 | 35 | FAM102A | MD17_01B | 5,221,308 | Intron |  |
| 17 | 35 | FAM102A | MD17_01C | 5,230,753 | Intron |  |
| 17 | 35 | DPM2 | MD17_02A | 5,237,745 | 66A>G | Val22 |
| 17 | 35 | DPM2 | MD17_02B | 5,239,548 | 3' |  |
| 17 | 35 | ST6GALNAC6 | MD17_03A | 5,245,311 | Upstream |  |
| 17 | 35 | ST6GALNAC6 | MD17_03B | 5,246,277 | 378C>T | Ser126 |
| 17 | 35 | ST6GALNAC6 | MD17_03C | 5,247,038 |  |  |
| 17 | 35 | ENG | MD17_04A | 5,258,665 | 108G>T | Pro36 |
| 17 | 35 | ENG | MD17_04B | 5,263,306 | Intergenic |  |
| 17 | 35 | CDK9 | MD17_05B | 5,269,149 | 3' |  |
| 17 | 35 | CDK9 | MD17_05A | 5,272,121 | 475C>A | Arg159 |
| 17 | 35 | SH2D3C | MD17_06A | 5,292,163 | 702T>C | Cys234 |
| 17 | 35 | SH2D3C | MD17_06B | 5,295,958 | 3' |  |
| 17 | 35 | TOR2A | MD17_07A | 5,296,779 | 288G>C | Ala96 |
| 17 | 35 | TOR2A | MD17_07B | 5,298,419 |  |  |
| 17 | 35 | URM1 | MD17_08A | 5,321,060 | 3' |  |

**Table S9.** Markers found by *in-silico* investigation of 7 of the identified QTLRs. GGA is the chromosome; QTLR is the QTLR serial number (Table 1); bp is the location on GRCg6a genome assembly; Location/alleles is the location respect to the gene or SNP alleles; AA is Amino Acid.

| **Marker** | **GGA** | **bp** | **Distance** | **Element** | **QTLR** | **Within Line** | | | | | | | | **Across Lines** |
| --- | --- | --- | --- | --- | --- | --- | --- | --- | --- | --- | --- | --- | --- | --- |
|  |  |  |  |  |  | **WL1** | **WL2** | **WL3** | **WPR1** | **WPR2** | **WL4** | **WL5** | **RIR1** |  |
| MD01_02A | 1 | 36,735,741 |  | TRHDE | 2 |  | 7.5E-04 |  |  |  | 8.6E-01 |  |  | 2.1E-03 |
| MD01_02B | 1 | 36,839,853 | 104,112 | TRHDE | 2 |  | 7.5E-04 |  | 3.8E-01 | 8.4E-01 | 7.8E-01 | 6.2E-01 | 9.1E-01 | 4.8E-02 |
| MD01_02C | 1 | 36,879,328 | 39,475 | TRHDE | 2 |  |  |  |  |  | 7.9E-01 |  | 1.1E-01 | 4.0E-01 |
| MD01_02D | 1 | 36,951,385 | 72,057 | TRHDE | 2 |  | 7.4E-02 |  | 8.8E-01 | 1.9E-01 |  |  | 9.1E-01 | 9.7E-01 |
| MD01_03A | 1 | 67,554,921 | 30,603,536 | RASSF8 | 4 | 7.1E-01 | 2.0E-01 | 4.2E-01 |  |  | 1.4E-01 | 3.2E-01 |  | 1.7E-01 |
| MD01_03B | 1 | 67,564,586 | 9,665 | RASSF8 | 4 | 8.4E-01 | 7.9E-01 | 2.4E-01 | 6.1E-01 | 1.9E-01 | 2.0E-01 | 3.2E-01 | 2.3E-01 | 7.7E-01 |
| MD01_03C | 1 | 67,569,296 | 4,710 | RASSF8 | 4 |  | 4.5E-01 | 1.4E-01 | 6.1E-01 | 1.9E-01 | 2.8E-01 | 1.2E-01 | 1.5E-01 | 3.6E-01 |
| MD01_04B | 1 | 67,629,704 | 60,408 | SSPN | 4 | 5.0E-01 | 3.2E-01 | 3.7E-01 | 7.9E-01 | 7.4E-01 | 1.4E-01 |  |  | 6.4E-01 |
| MD01_04C | 1 | 67,629,779 | 75 | SSPN | 4 | 5.3E-01 | 3.2E-01 | 3.7E-01 | 7.9E-01 | 6.7E-01 | 1.4E-01 |  |  | 6.6E-01 |
| MD01_05C | 1 | 67,659,080 | 29,301 | ITPR2 | 4 | 7.4E-01 | 4.8E-01 | 5.1E-01 |  |  | 6.3E-02 |  |  | 1.6E-01 |
| MD01_05B | 1 | 67,810,544 | 151,464 | ITPR2 | 4 | 7.8E-01 | 2.0E-01 | 8.2E-01 | 6.0E-01 |  | 6.1E-02 |  |  | 8.5E-02 |
| MD01_05A | 1 | 67,882,133 | 71,589 | ITPR2 | 4 | 3.6E-01 |  |  | 3.9E-01 | 1.1E-01 |  |  | 2.0E-01 | 8.2E-01 |
| MD04_02A | 4 | 39,453,244 |  | ACSL1 | 17 |  |  |  | 2.4E-01 | 8.5E-01 | 2.2E-01 |  | 7.2E-01 | 1.7E-07 |
| MD04_03C | 4 | 39,481,749 | 28,505 | CCDC111 | 17 |  | 1.6E-02 | 6.7E-01 | 7.3E-01 | 6.4E-01 | 1.9E-01 | 1.0E-01 |  | 6.5E-02 |
| MD04_03B | 4 | 39,483,799 | 2,050 | CCDC111 | 17 | 4.6E-01 | 5.8E-01 | 5.7E-01 | 2.2E-01 | 8.9E-01 | 1.6E-01 | 1.3E-01 | 7.3E-01 | 4.5E-01 |
| MD04_03A | 4 | 39,486,747 | 2,948 | CCDC111 | 17 | 3.4E-01 | 6.6E-03 | 6.7E-01 | 9.7E-01 | 8.8E-01 | 1.6E-01 | 5.8E-02 | 7.3E-01 | 5.7E-03 |
| MD04_04A | 4 | 39,498,984 | 12,237 | CASP3 | 17 | 3.6E-01 | 6.7E-03 | 4.7E-01 | 1.4E-01 | 7.0E-01 | 1.5E-01 |  |  | 6.8E-02 |
| MD04_04B | 4 | 39,506,324 | 7,340 | CASP3 | 17 | 2.6E-01 | 4.8E-01 | 2.0E-01 | 3.3E-01 | 6.7E-01 | 1.5E-01 |  |  | 9.3E-01 |
| MD04_04C | 4 | 39,509,637 | 3,313 | CASP3 | 17 | 3.0E-01 | 4.7E-03 | 4.7E-01 | 1.2E-01 | 6.7E-01 | 7.8E-01 | 6.7E-01 |  | 3.2E-01 |
| MD09_01B | 9 | 12,646,991 |  | DLG1 | 28 | 3.1E-01 | 2.5E-04 | 7.0E-01 | 3.5E-01 | 4.8E-01 | 1.3E-01 |  | 4.7E-02 | 2.5E-04 |
| MD09_01A | 9 | 12,691,840 | 44,849 | DLG1 | 28 |  | 3.1E-03 | 2.5E-01 | 4.3E-01 | 9.9E-01 | 9.9E-02 | 4.9E-01 | 1.2E-01 | 6.4E-04 |
| MD09_02A | 9 | 13,960,302 | 1,268,462 | GMNC | 28 |  |  |  |  |  | 5.0E-02 |  | 3.9E-01 | 2.9E-01 |
| MD09_02B | 9 | 13,967,979 | 7,677 | GMNC | 28 | 2.0E-01 | 2.1E-06 | 4.4E-01 | 3.3E-03 |  | 5.4E-02 | 1.6E-01 | 6.6E-01 | 1.1E-04 |
| MD09_03A | 9 | 13,991,344 | 23,365 | intergenic | 28 |  |  |  | 2.0E-04 | 3.9E-01 | 5.1E-01 |  | 9.4E-01 | 1.0E-02 |
| MD09_04A | 9 | 14,024,326 | 32,982 | IL1RAP | 28 |  |  | 6.9E-01 | 9.1E-02 |  |  |  | 9.4E-01 | 3.8E-01 |
| MD09_05Da | 9 | 14,044,716 | 20,390 | gga-mir-1762 | 28 | 8.2E-02 | 1.2E-06 | 5.6E-01 |  |  |  | 2.8E-02 | 2.0E-01 | 5.1E-04 |
| MD09_05Db | 9 | 14,044,717 | 1 | gga-mir-1762 | 28 | 8.7E-01 | 1.2E-06 | 5.6E-01 | 3.5E-04 |  |  | 2.8E-02 | 1.6E-01 | 5.4E-01 |
| MD09_05C | 9 | 14,044,719 | 2 | gga-mir-1762 | 28 | 1.2E-01 | 8.5E-07 | 5.0E-01 |  |  |  | 3.3E-02 | 2.0E-01 | 5.2E-04 |
| MD09_05A | 9 | 14,044,752 | 33 | gga-mir-1762 | 28 | 4.1E-01 |  | 6.9E-01 | 1.7E-03 | 6.8E-01 |  |  |  | 6.1E-03 |
| MD09_06A | 9 | 14,069,448 | 24,696 | TMEM207 | 28 |  |  |  | 1.1E-03 |  | 2.4E-01 |  | 1.8E-02 | 2.0E-04 |
| MD09_06B | 9 | 14,072,014 | 2,566 | TMEM207 | 28 |  | 1.6E-06 | 7.9E-01 | 1.2E-03 |  | 1.7E-01 | 3.2E-02 | 1.5E-01 | 8.2E-01 |
| MD09_06C | 9 | 14,073,188 | 1,174 | TMEM207 | 28 |  | 6.8E-06 | 8.5E-01 | 3.0E-03 |  | 2.4E-01 | 5.4E-02 | 5.5E-02 | 6.0E-01 |
| MD09_07C | 9 | 16,120,414 | 2,047,226 | EPHB | 28 |  | 9.0E-01 | 4.0E-01 |  |  | 5.6E-01 |  | 4.6E-01 | 8.3E-01 |
| MD09_07B | 9 | 16,122,876 | 2,462 | EPHB | 28 | 8.2E-01 | 4.0E-01 |  |  |  |  |  |  | 4.0E-01 |
| MD09_07A | 9 | 16,129,963 | 7,087 | EPHB | 28 | 9.3E-02 | 8.2E-01 | 2.4E-01 |  |  |  |  |  | 5.4E-01 |
| MD09_08A | 9 | 16,528,620 | 398,657 | LAMP3 | 28 | 8.4E-01 |  | 1.4E-01 |  |  |  |  |  | 1.6E-01 |
| MD09_08B | 9 | 16,532,498 | 3,878 | LAMP3 | 28 |  |  | 5.0E-01 | 6.6E-01 | 3.5E-01 | 4.9E-01 | 2.3E-01 | 5.8E-03 | 5.8E-04 |
| MD09_08C | 9 | 16,537,751 | 5,253 | LAMP3 | 28 |  |  | 5.4E-01 | 6.6E-01 | 3.5E-01 | 2.4E-01 | 2.3E-01 | 5.8E-03 | 1.1E-03 |
| MD09_08D | 9 | 16,540,194 | 2,443 | LAMP3 | 28 | 8.1E-01 | 7.9E-01 | 2.0E-01 |  |  | 2.4E-01 | 7.2E-01 |  | 1.4E-01 |
| MD13_03A | 13 | 4,954,745 |  | SLIT3 | 31 |  |  |  | 4.1E-01 | 1.2E-01 |  |  |  | 1.3E-01 |
| MD13_03B | 13 | 5,324,164 | 369,419 | SLIT3 | 31 | 7.7E-01 | 9.2E-01 | 8.0E-01 | 8.3E-01 | 8.6E-01 | 9.1E-01 | 5.2E-01 | 4.9E-01 | 6.5E-01 |
| MD13_03C | 13 | 5,421,966 | 97,802 | SLIT3 | 31 | 9.1E-01 | 8.1E-01 | 9.8E-01 |  |  | 9.3E-01 |  | 2.1E-01 | 9.5E-01 |
| MD14_01A | 14 | 14,030,671 | 8,694,778 | HN1L | 34 | 1.3E-03 | 4.6E-01 | 4.5E-01 | 9.1E-01 | 9.7E-01 | 5.7E-01 |  | 1.4E-01 | 7.9E-01 |
| MD14_02A | 14 | 14,050,330 | 19,659 | CRAMP1 | 34 | 1.3E-03 | 4.6E-01 | 9.6E-01 |  | 7.8E-01 | 6.3E-02 | 2.7E-03 | 8.9E-01 | 1.4E-03 |
| MD14_03B | 14 | 14,115,781 | 65,451 | TMEM204 | 34 | 1.3E-03 | 4.6E-01 | 4.5E-01 | 9.1E-01 | 9.8E-01 |  |  |  | 5.6E-01 |
| MD14_03A | 14 | 14,142,117 | 26,336 | TMEM204 | 34 | 4.9E-04 | 4.9E-01 | 7.5E-01 | 3.7E-01 |  | 8.4E-01 | 1.0E-02 | 1.8E-02 | 3.1E-01 |
| MD14_04A | 14 | 14,252,012 | 109,895 | ATP6V0C | 34 | 5.4E-02 |  |  | 3.3E-01 | 5.1E-01 |  | 6.7E-01 | 2.1E-01 | 6.6E-01 |
| MD17_01A | 17 | 5,203,358 |  | FAM102A | 35 |  | 7.7E-01 | 1.1E-01 | 4.4E-01 | 7.5E-01 | 9.1E-01 |  | 5.1E-01 | 7.4E-01 |
| MD17_01B | 17 | 5,221,308 | 17,950 | FAM102A | 35 | 6.2E-02 |  |  | 9.7E-01 | 2.8E-01 |  |  | 6.2E-01 | 4.9E-01 |
| MD17_01C | 17 | 5,230,753 | 9,445 | FAM102A | 35 | 5.3E-02 | 2.6E-01 | 1.3E-01 | 8.8E-01 | 2.8E-01 | 6.8E-02 |  | 9.5E-01 | 6.9E-02 |
| MD17_02A | 17 | 5,237,745 | 6,992 | DPM2 | 35 |  | 6.9E-01 | 1.6E-01 | 7.4E-01 | 3.1E-01 | 8.6E-01 |  | 4.1E-01 | 1.9E-01 |
| MD17_02B | 17 | 5,239,548 | 1,803 | DPM2 | 35 | 6.2E-02 | 5.2E-01 | 1.5E-01 | 9.6E-01 | 4.4E-01 | 6.7E-02 |  | 9.4E-01 | 1.3E-01 |
| MD17_03A | 17 | 5,245,311 | 5,763 | ST6GALNAC6 | 35 |  |  | 4.0E-01 | 6.0E-01 | 6.1E-01 | 8.1E-01 | 6.7E-01 | 1.3E-01 | 8.3E-01 |
| MD17_03B | 17 | 5,246,277 | 966 | ST6GALNAC6 | 35 | 5.4E-02 |  |  | 6.0E-01 |  |  |  | 8.1E-01 | 6.8E-01 |
| MD17_03C | 17 | 5,247,038 | 761 | ST6GALNAC6 | 35 | 6.6E-02 |  |  | 6.5E-01 |  |  | 6.8E-01 | 8.0E-01 | 6.6E-01 |
| MD17_04A | 17 | 5,258,665 | 11,627 | ENG | 35 |  | 4.0E-01 | 8.1E-02 |  |  |  |  |  | 2.3E-01 |
| MD17_04B | 17 | 5,263,306 | 4,641 | ENG | 35 | 6.3E-02 | 1.5E-01 | 1.3E-01 | 6.2E-01 | 8.7E-01 |  |  | 4.2E-01 | 6.7E-02 |
| MD17_05B | 17 | 5,269,149 | 5,843 | CDK10 | 35 | 5.9E-02 | 2.2E-01 | 2.1E-01 | 3.5E-01 |  | 7.9E-01 |  | 2.6E-01 | 1.8E-02 |
| MD17_05A | 17 | 5,272,121 | 2,972 | CDK11 | 35 |  | 7.8E-01 | 6.0E-01 | 7.0E-01 | 2.0E-01 |  |  | 6.4E-01 | 7.5E-01 |
| MD17_06A | 17 | 5,292,163 | 20,042 | SH2D3C | 35 | 4.6E-02 | 2.2E-01 | 9.1E-01 | 3.6E-01 | 9.6E-01 | 7.4E-01 |  | 1.2E-01 | 4.9E-01 |
| MD17_06B | 17 | 5,295,958 | 3,795 | SH2D3C | 35 | 5.8E-02 | 4.3E-01 | 6.8E-01 | 7.8E-01 | 4.3E-01 |  |  | 1.2E-01 | 8.9E-01 |
| MD17_07A | 17 | 5,296,779 | 821 | TOR2A | 35 | 5.1E-02 | 7.1E-01 | 9.5E-01 | 7.2E-01 | 4.8E-01 | 3.9E-01 |  |  | 4.5E-01 |
| MD17_07B | 17 | 5,298,419 | 1,640 | TOR2A | 35 | 4.5E-02 |  |  | 3.1E-01 | 4.9E-01 |  |  | 1.6E-01 | 6.8E-01 |
| MD17_08A | 17 | 5,321,060 | 22,641 | URM1 | 35 | 5.1E-02 | 5.1E-01 | 9.4E-01 | 1.7E-01 | 9.7E-01 | 9.9E-01 |  |  | 1.2E-01 |

**Table S10.** Association tests of QTLR markers, by location (GRCg6a). GGA is the chromosome; bp is the location on the chromosome; Distance are the bp between the marker and the previous marker; Element is the QTLR genomic element (Table S8); QTLR is the QTLR serial number (Table 1): QTLRs are separated by double lines; Within Line is a *P*-value of a test within a line: pink highlight, *P* ≤ 0.05; white, *P* > 0.05; Across Lines is a *P*-value of a test across all lines: pink highlight, *p* ≤ 0.05; white, *P* > 0.05.

| **Line** | ***cP*** | ***cG*** |
| --- | --- | --- |
| WL1 | 0.032 | 0.250 |
| WL2 | 0.125 | 0.962 |
| WL3 | - | - |
| WPR1 | 0.052 | 0.398 |
| WPR2 | - | - |
| WL4 | - | - |
| WL5 | 0.006 | 0.045 |
| RIR1 | 0.016 | 0.125 |
| Average | 0.046 | 0.356 |
| Minimum | 0.006 | 0.045 |
| Maximum | 0.125 | 0.962 |

**Table S11.** Contribution to the phenotypic (*cP*) and genotypic (*cG*) variations obtained by individual genotyping of markers in the regions tested. “-“: no QTLR marker was significant (*p* ≤ 0.05) in Lines WL3, WPR2 or WL4 (Table S10).

**Table S12.** Linkage disequilibrium matrices of all markers on the same chromosome within lines.

Chr is the chromosome; Element is the QTLR genomic element (Table S8): different elements have different colours; bp is the location on the GRCg6a genome assembly; Dist. are bp between the marker and the previous marker; QTLR is the QTLR serial number found by the Pools (Table 1): different QTLRs on the same chromosome have different colours; Marker is the marker tested (Table S9); LD values: red, r^2^ ≥ 0.7; pink, 0.15 ≥ r^2^ < 0.7; white, r^2^ < 0.15; P is the P-value of the Trend association test (Table S9): pink, P ≤ 0.05; white, P > 0.05; |α| is the absolute marker allele substitution effect; cP is the marker contribution to the population phenotypic variation; cG is the marker contribution to the population genotypic variation. The LD matrices were created by JMP Genomics, and then more data were added.

| **Chr 1** | | | | | | | | | | | |
| --- | --- | --- | --- | --- | --- | --- | --- | --- | --- | --- | --- |
| Element | | | | | RASSF8 | RASSF8 | SSPN | SSPN | ITPR2 | ITPR2 | ITPR2 |
|  | bp | | | | 67,554,921 | 67,564,586 | 67,629,704 | 67,629,779 | 67,659,080 | 67,810,544 | 67,882,133 |
|  |  | Dist. | | |  | 9,665 | 65,118 | 75 | 29,301 | 151,464 | 71,589 |
|  |  |  | QTLR | | 4 | 4 | 4 | 4 | 4 | 4 | 4 |
|  |  |  |  | Marker | MD01_03A | MD01_03B | MD01_04B | MD01_04C | MD01_05C | MD01_05B | MD01_05A |
| RASSF8 | 67,554,921 |  | 4 | MD01_03A |  |  |  |  |  |  |  |
| RASSF8 | 67,564,586 | 9,665 | 4 | MD01_03B | 0.330 |  |  |  |  |  |  |
| SSPN | 67,629,704 | 65,118 | 4 | MD01_04B | 0.485 | 0.006 |  |  |  |  |  |
| SSPN | 67,629,779 | 75 | 4 | MD01_04C | 0.484 | 0.006 | 1.000 |  |  |  |  |
| ITPR2 | 67,659,080 | 29,301 | 4 | MD01_05C | 0.704 | 0.121 | 0.574 | 0.574 |  |  |  |
| ITPR2 | 67,810,544 | 151,464 | 4 | MD01_05B | 0.639 | 0.084 | 0.590 | 0.591 | 0.904 |  |  |
| ITPR2 | 67,882,133 | 71,589 | 4 | MD01_05A | 0.000 | 0.000 | 0.001 | 0.001 | 0.001 | 0.001 |  |
|  | | | | P: | 7.13E-01 | 8.40E-01 | 5.00E-01 | 5.28E-01 | 7.42E-01 | 7.83E-01 | 3.63E-01 |
|  |  |  |  | \|α\|: | 0.1997 | 0.1251 | 0.4113 | 0.3848 | 0.1837 | 0.1547 | 9.8808 |
|  |  |  |  | cP: | 0.0001 | 0.0000 | 0.0003 | 0.0003 | 0.0001 | 0.0001 | 0.0003 |
|  |  |  |  | cG: | 0.0007 | 0.0002 | 0.0024 | 0.0021 | 0.0006 | 0.0004 | 0.0024 |

| **Chr 4** | | | | | | | | | |
| --- | --- | --- | --- | --- | --- | --- | --- | --- | --- |
| Element | | | | | CCDC111 | CCDC111 | CASP3 | CASP3 | CASP3 |
|  | bp | | | | 39,483,799 | 39,486,747 | 39,498,984 | 39,506,324 | 39,509,637 |
|  |  | Dist. | | |  | 2,948 | 12,237 | 7,340 | 3,313 |
|  |  |  | QTLR | | 17 | 17 | 17 | 17 | 17 |
|  |  |  |  | Marker | MD04_03B | MD04_03A | MD04_04A | MD04_04B | MD04_04C |
| CCDC111 | 39,483,799 |  | 17 | MD04_03B |  |  |  |  |  |
| CCDC111 | 39,486,747 | 2,948 | 17 | MD04_03A | 0.060 |  |  |  |  |
| CASP3 | 39,498,984 | 12,237 | 17 | MD04_04A | 0.060 | 0.992 |  |  |  |
| CASP3 | 39,506,324 | 7,340 | 17 | MD04_04B | 0.986 | 0.059 | 0.059 |  |  |
| CASP3 | 39,509,637 | 3,313 | 17 | MD04_04C | 0.059 | 0.978 | 0.983 | 0.058 |  |
|  | | | | P: | 4.63E-01 | 3.39E-01 | 3.56E-01 | 2.61E-01 | 3.00E-01 |
|  |  |  |  | \|α\|: | 0.6900 | 0.5074 | 0.4933 | 1.0652 | 0.5543 |
|  |  |  |  | cP: | 0.0003 | 0.0006 | 0.0006 | 0.0008 | 0.0007 |
|  |  |  |  | cG: | 0.0025 | 0.0046 | 0.0043 | 0.0059 | 0.0054 |

| **Chr 9** | | | | | | | | | | | | | | |
| --- | --- | --- | --- | --- | --- | --- | --- | --- | --- | --- | --- | --- | --- | --- |
| Element | | | | | DLG1 | GMNC | gga-mir-1762 | gga-mir-1762 | gga-mir-1762 | gga-mir-1762 | EPHB | EPHB | LAMP3 | LAMP3 |
|  | bp | | | | 12,646,991 | 13,967,979 | 14,044,716 | 14,044,717 | 14,044,719 | 14,044,752 | 16,122,876 | 16,129,963 | 16,528,620 | 16,540,194 |
|  |  | Dist. | | |  | 1,320,988 | 76,737 | 1 | 2 | 33 | 2,078,124 | 7,087 | 398,657 | 11,574 |
|  |  |  | QTLR | | 28 | 28 | 28 | 28 | 28 | 28 | 28 | 28 | 28 | 28 |
|  |  |  |  | Marker | MD09_01B | MD09_02B | MD09_05Da | MD09_05Db | MD09_05C | MD09_05A | MD09_07B | MD09_07A | MD09_08A | MD09_08D |
| DLG1 | 12,646,991 |  | 28 | MD09_01B |  |  |  |  |  |  |  |  |  |  |
| GMNC | 13,967,979 | 1,320,988 | 28 | MD09_02B | 0.002 |  |  |  |  |  |  |  |  |  |
| gga-mir-1762 | 14,044,716 | 76,737 | 28 | MD09_05Da | 0.024 | 0.594 |  |  |  |  |  |  |  |  |
| gga-mir-1762 | 14,044,717 | 1 | 28 | MD09_05Db | 0.164 | 0.074 | 0.134 |  |  |  |  |  |  |  |
| gga-mir-1762 | 14,044,719 | 2 | 28 | MD09_05C | 0.001 | 0.874 | 0.663 | 0.088 |  |  |  |  |  |  |
| gga-mir-1762 | 14,044,752 | 33 | 28 | MD09_05A | 0.178 | 0.001 | 0.019 | 0.883 | 0.001 |  |  |  |  |  |
| EPHB | 16,122,876 | 2,078,124 | 28 | MD09_07B | 0.002 | 0.001 | 0.000 | 0.004 | 0.001 | 0.007 |  |  |  |  |
| EPHB | 16,129,963 | 7,087 | 28 | MD09_07A | 0.000 | 0.005 | 0.002 | 0.002 | 0.006 | 0.005 | 0.386 |  |  |  |
| LAMP3 | 16,528,620 | 398,657 | 28 | MD09_08A | 0.003 | 0.000 | 0.000 | 0.000 | 0.000 | 0.000 | 0.000 | 0.108 |  |  |
| LAMP3 | 16,540,194 | 11,574 | 28 | MD09_08D | 0.000 | 0.000 | 0.000 | 0.000 | 0.000 | 0.000 | 0.000 | 0.109 | 0.939 |  |
|  | | | | P: | 3.07E-01 | 2.00E-01 | 8.16E-02 | 8.66E-01 | 1.22E-01 | 4.11E-01 | 8.20E-01 | 9.28E-02 | 8.39E-01 | 8.12E-01 |
|  |  |  |  | \|α\|: | 0.9633 | 2.5308 | 2.8157 | 0.1208 | 3.1079 | 0.6188 | 0.1203 | 1.0412 | 0.1203 | 0.1382 |
|  |  |  |  | cP: | 0.0005 | 0.0008 | 0.0013 | 0.0000 | 0.0011 | 0.0003 | 0.0000 | 0.0018 | 0.0000 | 0.0000 |
|  |  |  |  | cG: | 0.0036 | 0.0063 | 0.0099 | 0.0001 | 0.0081 | 0.0026 | 0.0003 | 0.0142 | 0.0002 | 0.0003 |

| **Chr 13** | | | | | | |
| --- | --- | --- | --- | --- | --- | --- |
| Element | | | | | SLIT3 | SLIT3 |
|  | bp | | | | 5,324,164 | 5,421,966 |
|  |  | Dist. | | |  | 97,802 |
|  |  |  | QTLR | | 31 | 31 |
|  |  |  |  | Marker | MD13_03B | MD13_03C |
| SLIT3 | 5,324,164 |  | 31 | MD13_03B |  |  |
| SLIT3 | 5,421,966 | 97,802 | 31 | MD13_03C | 0.502 |  |
|  | | | | P: | 7.7E-01 | 9.1E-01 |
|  |  |  |  | \|α\|: | 0.1475 | 0.0583 |
|  |  |  |  | cP: | 0.0001 | 0.0000 |
|  |  |  |  | cG: | 0.0004 | 0.0001 |

| **Chr 14** | | | | | | | | | |
| --- | --- | --- | --- | --- | --- | --- | --- | --- | --- |
| Element | | | | | HN1L | CRAMP1 | TMEM204 | TMEM204 | ATP6V0C |
|  | bp | | | | 14,030,671 | 14,050,330 | 14,115,781 | 14,142,117 | 14,252,012 |
|  |  | Dist. | | |  | 19,659 | 65,451 | 26,336 | 109,895 |
|  |  |  | QTLR | | 34 | 34 | 34 | 34 | 34 |
|  |  |  |  | Marker | MD14_01A | MD14_02A | MD14_03B | MD14_03A | MD14_04A |
| HN1L | 14,030,671 |  | 34 | MD14_01A |  |  |  |  |  |
| CRAMP1 | 14,050,330 | 19,659 | 34 | MD14_02A | 1.000 |  |  |  |  |
| TMEM204 | 14,115,781 | 65,451 | 34 | MD14_03B | 1.000 | 1.000 |  |  |  |
| TMEM204 | 14,142,117 | 26,336 | 34 | MD14_03A | 0.997 | 0.997 | 0.997 |  |  |
| ATP6V0C | 14,252,012 | 109,895 | 34 | MD14_04A | 0.011 | 0.011 | 0.011 | 0.011 |  |
|  | | | | P: | 1.27E-03 | 1.27E-03 | 1.27E-03 | 4.87E-04 | 5.42E-02 |
|  |  |  |  | \|α\|: | 1.6758 | 1.6758 | 1.6758 | 1.8190 | 1.4131 |
|  |  |  |  | cP: | 0.0066 | 0.0066 | 0.0066 | 0.0078 | 0.0022 |
|  |  |  |  | cG: | 0.0511 | 0.0511 | 0.0511 | 0.0602 | 0.0168 |

| **Chr 17** | | | | | | | | | | | | | | | | |
| --- | --- | --- | --- | --- | --- | --- | --- | --- | --- | --- | --- | --- | --- | --- | --- | --- |
| Element | | | | | FAM102A | FAM102A | DPM2 | ST6GALNAC6 | ST6GALNAC6 | ENG | CDK10 | SH2D3C | SH2D3C | TOR2A | TOR2A | URM1 |
|  | bp | | | | 5,221,308 | 5,230,753 | 5,239,548 | 5,246,277 | 5,247,038 | 5,263,306 | 5,269,149 | 5,292,163 | 5,295,958 | 5,296,779 | 5,298,419 | 5,321,060 |
|  |  | Dist. | | |  | 9,445 | 8,795 | 6,729 | 761 | 16,268 | 5,843 | 23,014 | 3,795 | 821 | 1,640 | 22,641 |
|  |  |  | QTLR | | 35 | 35 | 35 | 35 | 35 | 35 | 35 | 35 | 35 | 35 | 35 | 35 |
|  |  |  |  | Marker | MD17_01B | MD17_01C | MD17_02B | MD17_03B | MD17_03C | MD17_04B | MD17_05B | MD17_06A | MD17_06B | MD17_07A | MD17_07B | MD17_08A |
| FAM102A | 5,221,308 |  | 35 | MD17_01B |  |  |  |  |  |  |  |  |  |  |  |  |
| FAM102A | 5,230,753 | 9,445 | 35 | MD17_01C | 0.994 |  |  |  |  |  |  |  |  |  |  |  |
| DPM2 | 5,239,548 | 8,795 | 35 | MD17_02B | 1.000 | 0.994 |  |  |  |  |  |  |  |  |  |  |
| ST6GALNAC6 | 5,246,277 | 6,729 | 35 | MD17_03B | 0.994 | 0.994 | 0.994 |  |  |  |  |  |  |  |  |  |
| ST6GALNAC6 | 5,247,038 | 761 | 35 | MD17_03C | 1.000 | 0.994 | 1.000 | 0.994 |  |  |  |  |  |  |  |  |
| ENG | 5,263,306 | 16,268 | 35 | MD17_04B | 0.985 | 0.982 | 0.985 | 0.988 | 0.985 |  |  |  |  |  |  |  |
| CDK10 | 5,269,149 | 5,843 | 35 | MD17_05B | 1.000 | 0.994 | 1.000 | 0.994 | 1.000 | 0.985 |  |  |  |  |  |  |
| SH2D3C | 5,292,163 | 23,014 | 35 | MD17_06A | 0.964 | 0.964 | 0.964 | 0.970 | 0.964 | 0.976 | 0.964 |  |  |  |  |  |
| SH2D3C | 5,295,958 | 3,795 | 35 | MD17_06B | 0.934 | 0.934 | 0.933 | 0.940 | 0.934 | 0.945 | 0.933 | 0.968 |  |  |  |  |
| TOR2A | 5,296,779 | 821 | 35 | MD17_07A | 0.934 | 0.934 | 0.934 | 0.940 | 0.934 | 0.945 | 0.934 | 0.968 | 1.000 |  |  |  |
| TOR2A | 5,298,419 | 1,640 | 35 | MD17_07B | 0.934 | 0.934 | 0.933 | 0.940 | 0.934 | 0.945 | 0.934 | 0.968 | 1.000 | 1.000 |  |  |
| URM1 | 5,321,060 | 22,641 | 35 | MD17_08A | 0.915 | 0.912 | 0.914 | 0.918 | 0.915 | 0.923 | 0.914 | 0.945 | 0.974 | 0.974 | 0.974 |  |
|  | | | | P: | 6.18E-02 | 5.32E-02 | 6.25E-02 | 5.37E-02 | 6.55E-02 | 6.31E-02 | 5.89E-02 | 4.57E-02 | 5.76E-02 | 5.05E-02 | 4.49E-02 | 5.12E-02 |
|  |  |  |  | \|α\|: | 1.3732 | 1.4220 | 1.3685 | 1.4146 | 1.3536 | 1.3731 | 1.3880 | 1.4820 | 1.4013 | 1.4425 | 1.4791 | 1.4266 |
|  |  |  |  | cP: | 0.0020 | 0.0022 | 0.0020 | 0.0022 | 0.0020 | 0.0020 | 0.0021 | 0.0023 | 0.0021 | 0.0023 | 0.0024 | 0.0023 |
|  |  |  |  | cG: | 0.0155 | 0.0166 | 0.0154 | 0.0165 | 0.0151 | 0.0155 | 0.0158 | 0.0180 | 0.0165 | 0.0175 | 0.0184 | 0.0174 |

| **Chr 1** | | | | | | | | | | | | | | |
| --- | --- | --- | --- | --- | --- | --- | --- | --- | --- | --- | --- | --- | --- | --- |
| Element | | | | | TRHDE | TRHDE | TRHDE | RASSF8 | RASSF8 | RASSF8 | SSPN | SSPN | ITPR2 | ITPR2 |
|  | bp | | | | 36,735,741 | 36,839,853 | 36,951,385 | 67,554,921 | 67,564,586 | 67,569,296 | 67,629,704 | 67,629,779 | 67,659,080 | 67,810,544 |
|  |  | Dist. | | |  | 104,112 | 111,532 | 30,603,536 | 9,665 | 4,710 | 60,408 | 75 | 29,301 | 151,464 |
|  |  |  | QTLR | | 2 | 2 | 2 | 4 | 4 | 4 | 4 | 4 | 4 | 4 |
|  |  |  |  | Marker | MD01_02A | MD01_02B | MD01_02D | MD01_03A | MD01_03B | MD01_03C | MD01_04B | MD01_04C | MD01_05C | MD01_05B |
| TRHDE | 36,735,741 |  | 2 | MD01_02A |  |  |  |  |  |  |  |  |  |  |
| TRHDE | 36,839,853 | 104,112 | 2 | MD01_02B | 1.000 |  |  |  |  |  |  |  |  |  |
| TRHDE | 36,951,385 | 111,532 | 2 | MD01_02D | 0.864 | 0.864 |  |  |  |  |  |  |  |  |
| RASSF8 | 67,554,921 | 30,603,536 | 4 | MD01_03A | 0.000 | 0.000 | 0.001 |  |  |  |  |  |  |  |
| RASSF8 | 67,564,586 | 9,665 | 4 | MD01_03B | 0.004 | 0.004 | 0.002 | 0.092 |  |  |  |  |  |  |
| RASSF8 | 67,569,296 | 4,710 | 4 | MD01_03C | 0.003 | 0.003 | 0.000 | 0.060 | 0.683 |  |  |  |  |  |
| SSPN | 67,629,704 | 60,408 | 4 | MD01_04B | 0.002 | 0.002 | 0.000 | 0.219 | 0.056 | 0.272 |  |  |  |  |
| SSPN | 67,629,779 | 75 | 4 | MD01_04C | 0.002 | 0.002 | 0.000 | 0.219 | 0.056 | 0.272 | 1.000 |  |  |  |
| ITPR2 | 67,659,080 | 29,301 | 4 | MD01_05C | 0.001 | 0.001 | 0.001 | 0.255 | 0.026 | 0.220 | 0.835 | 0.835 |  |  |
| ITPR2 | 67,810,544 | 151,464 | 4 | MD01_05B | 0.000 | 0.000 | 0.001 | 0.955 | 0.091 | 0.062 | 0.222 | 0.222 | 0.261 |  |
|  | | | | P: | 7.48E-04 | 7.48E-04 | 7.42E-02 | 1.99E-01 | 7.87E-01 | 4.46E-01 | 3.17E-01 | 3.17E-01 | 4.81E-01 | 1.98E-01 |
|  |  |  |  | \|α\|: | 2.2243 | 2.2243 | 1.2183 | 1.5910 | 0.1729 | 0.5036 | 0.6715 | 0.6715 | 0.5014 | 1.5974 |
|  |  |  |  | cP: | 0.0071 | 0.0071 | 0.0020 | 0.0011 | 0.0000 | 0.0004 | 0.0006 | 0.0006 | 0.0003 | 0.0011 |
|  |  |  |  | cG: | 0.0547 | 0.0547 | 0.0157 | 0.0087 | 0.0004 | 0.0030 | 0.0043 | 0.0043 | 0.0022 | 0.0086 |

| **Chr 4** | | | | | | | | | | |
| --- | --- | --- | --- | --- | --- | --- | --- | --- | --- | --- |
| Element | | | | | CCDC111 | CCDC111 | CCDC111 | CASP3 | CASP3 | CASP3 |
|  | bp | | | | 39,481,749 | 39,483,799 | 39,486,747 | 39,498,984 | 39,506,324 | 39,509,637 |
|  |  | Dist. | | |  | 2,050 | 2,948 | 12,237 | 7,340 | 3,313 |
|  |  |  | QTLR | | 17 | 17 | 17 | 17 | 17 | 17 |
|  |  |  |  | Marker | MD04_03C | MD04_03B | MD04_03A | MD04_04A | MD04_04B | MD04_04C |
| CCDC111 | 39,481,749 |  | 17 | MD04_03C |  |  |  |  |  |  |
| CCDC111 | 39,483,799 | 2,050 | 17 | MD04_03B | 0.079 |  |  |  |  |  |
| CCDC111 | 39,486,747 | 2,948 | 17 | MD04_03A | 0.672 | 0.116 |  |  |  |  |
| CASP3 | 39,498,984 | 12,237 | 17 | MD04_04A | 0.643 | 0.099 | 0.955 |  |  |  |
| CASP3 | 39,506,324 | 7,340 | 17 | MD04_04B | 0.076 | 0.940 | 0.112 | 0.116 |  |  |
| CASP3 | 39,509,637 | 3,313 | 17 | MD04_04C | 0.646 | 0.098 | 0.956 | 1.000 | 0.116 |  |
|  | | | | P: | 1.56E-02 | 5.80E-01 | 6.58E-03 | 6.73E-03 | 4.81E-01 | 4.67E-03 |
|  |  |  |  | \|α\|: | 1.7276 | 0.4224 | 1.8327 | 1.8185 | 0.5376 | 1.9137 |
|  |  |  |  | cP: | 0.0034 | 0.0002 | 0.0045 | 0.0045 | 0.0003 | 0.0050 |
|  |  |  |  | cG: | 0.0261 | 0.0014 | 0.0349 | 0.0348 | 0.0021 | 0.0386 |

| **Chr 9** | | | | | | | | | | | | | | | | |
| --- | --- | --- | --- | --- | --- | --- | --- | --- | --- | --- | --- | --- | --- | --- | --- | --- |
| Element | | | | | DLG1 | DLG1 | GMNC | gga-mir-1762 | gga-mir-1762 | gga-mir-1762 | TMEM207 | TMEM207 | EPHB | EPHB | EPHB | LAMP3 |
|  | bp | | | | 12,646,991 | 12,691,840 | 13,967,979 | 14,044,716 | 14,044,717 | 14,044,719 | 14,072,014 | 14,073,188 | 16,120,414 | 16,122,876 | 16,129,963 | 16,540,194 |
|  |  | Dist. | | |  | 44,849 | 1,276,139 | 76,737 | 1 | 2 | 27,295 | 1,174 | 2,047,226 | 2,462 | 7,087 | 410,231 |
|  |  |  | QTLR | | 28 | 28 | 28 | 28 | 28 | 28 | 28 | 28 | 28 | 28 | 28 | 28 |
|  |  |  |  | Marker | MD09_01B | MD09_01A | MD09_02B | MD09_05Da | MD09_05Db | MD09_05C | MD09_06B | MD09_06C | MD09_07C | MD09_07B | MD09_07A | MD09_08D |
| DLG1 | 12,646,991 |  | 28 | MD09_01B |  |  |  |  |  |  |  |  |  |  |  |  |
| DLG1 | 12,691,840 | 44,849 | 28 | MD09_01A | 0.810 |  |  |  |  |  |  |  |  |  |  |  |
| GMNC | 13,967,979 | 1,276,139 | 28 | MD09_02B | 0.017 | 0.008 |  |  |  |  |  |  |  |  |  |  |
| gga-mir-1762 | 14,044,716 | 76,737 | 28 | MD09_05Da | 0.018 | 0.009 | 0.973 |  |  |  |  |  |  |  |  |  |
| gga-mir-1762 | 14,044,717 | 1 | 28 | MD09_05Db | 0.018 | 0.009 | 0.973 | 1.000 |  |  |  |  |  |  |  |  |
| gga-mir-1762 | 14,044,719 | 2 | 28 | MD09_05C | 0.018 | 0.009 | 0.973 | 1.000 | 1.000 |  |  |  |  |  |  |  |
| TMEM207 | 14,072,014 | 27,295 | 28 | MD09_06B | 0.020 | 0.010 | 0.951 | 0.975 | 0.975 | 0.974 |  |  |  |  |  |  |
| TMEM207 | 14,073,188 | 1,174 | 28 | MD09_06C | 0.020 | 0.010 | 0.950 | 0.975 | 0.975 | 0.974 | 1.000 |  |  |  |  |  |
| EPHB | 16,120,414 | 2,047,226 | 28 | MD09_07C | 0.001 | 0.001 | 0.005 | 0.005 | 0.005 | 0.004 | 0.006 | 0.006 |  |  |  |  |
| EPHB | 16,122,876 | 2,462 | 28 | MD09_07B | 0.000 | 0.001 | 0.001 | 0.003 | 0.003 | 0.003 | 0.002 | 0.002 | 0.045 |  |  |  |
| EPHB | 16,129,963 | 7,087 | 28 | MD09_07A | 0.001 | 0.001 | 0.004 | 0.004 | 0.004 | 0.003 | 0.005 | 0.005 | 0.991 | 0.046 |  |  |
| LAMP3 | 16540194 | 410231 | 28 | MD09_08D | 0.001 | 0.001 | 0.001 | 0.001 | 0.001 | 0.001 | 0.001 | 0.000 | 0.001 | 0.000 | 0.001 |  |
|  | | | | P: | 2.47E-04 | 3.07E-03 | 2.07E-06 | 1.21E-06 | 1.21E-06 | 8.48E-07 | 1.64E-06 | 6.79E-06 | 9.00E-01 | 4.01E-01 | 8.16E-01 | 7.88E-01 |
|  |  |  |  | \|α\|: | 2.3801 | 1.9159 | 3.0081 | 3.0569 | 3.0569 | 3.1031 | 3.0339 | 2.8748 | 0.0839 | 1.0406 | 0.1543 | 2.9211 |
|  |  |  |  | cP: | 0.0081 | 0.0053 | 0.0132 | 0.0136 | 0.0136 | 0.0140 | 0.0134 | 0.0120 | 0.0000 | 0.0005 | 0.0000 | 0.0000 |
|  |  |  |  | cG: | 0.0624 | 0.0409 | 0.1018 | 0.1048 | 0.1048 | 0.1081 | 0.1029 | 0.0924 | 0.0001 | 0.0038 | 0.0003 | 0.0001 |

| **Chr 13** | | | | | | |
| --- | --- | --- | --- | --- | --- | --- |
| Element | | | | | SLIT3 | SLIT3 |
|  | bp | | | | 5,324,164 | 5,421,966 |
|  |  | Dist. | | |  | 97,802 |
|  |  |  | QTLR | | 31 | 31 |
|  |  |  |  | Marker | MD13_03B | MD13_03C |
| SLIT3 | 5,324,164 |  | 31 | MD13_03B |  |  |
| SLIT3 | 5,421,966 | 97,802 | 31 | MD13_03C | 0.788 |  |
|  | | | | P: | 9.23E-01 | 8.10E-01 |
|  |  |  |  | \|α\|: | 0.0627 | 0.1572 |
|  |  |  |  | cP: | 0.0000 | 0.0000 |
|  |  |  |  | cG: | 0.0000 | 0.0003 |

| **Chr 14** | | | | | | | | |
| --- | --- | --- | --- | --- | --- | --- | --- | --- |
| Element | | | | | HN1L | CRAMP1 | TMEM204 | TMEM204 |
|  | bp | | | | 14,030,671 | 14,050,330 | 14,115,781 | 14,142,117 |
|  |  | Dist. | | |  | 19,659 | 65,451 | 26,336 |
|  |  |  | QTLR | | 34 | 34 | 34 | 34 |
|  |  |  |  | Marker | MD14_01A | MD14_02A | MD14_03B | MD14_03A |
| HN1L | 14,030,671 |  | 34 | MD14_01A |  |  |  |  |
| CRAMP1 | 14,050,330 | 19,659 | 34 | MD14_02A | 1.000 |  |  |  |
| TMEM204 | 14,115,781 | 65,451 | 34 | MD14_03B | 1.000 | 1.000 |  |  |
| TMEM204 | 14,142,117 | 26,336 | 34 | MD14_03A | 0.996 | 0.996 | 0.996 |  |
|  | | | | P: | 4.55E-01 | 4.55E-01 | 4.55E-01 | 4.91E-01 |
|  |  |  |  | \|α\|: | 0.5471 | 0.5471 | 0.5471 | 0.5090 |
|  |  |  |  | cP: | 0.0003 | 0.0003 | 0.0003 | 0.0003 |
|  |  |  |  | cG: | 0.0025 | 0.0025 | 0.0025 | 0.0022 |

| **Chr 17** | | | | | | | | | | | | | | | | |
| --- | --- | --- | --- | --- | --- | --- | --- | --- | --- | --- | --- | --- | --- | --- | --- | --- |
| Element | | | | | FAM102A | FAM102A | DPM2 | DPM2 | ENG | ENG | CDK10 | CDK11 | SH2D3C | SH2D3C | TOR2A | URM1 |
|  | bp | | | | 5,203,358 | 5,230,753 | 5,237,745 | 5,239,548 | 5,258,665 | 5,263,306 | 5,269,149 | 5,272,121 | 5,292,163 | 5,295,958 | 5,296,779 | 5,321,060 |
|  |  | Dist. | | |  | 27,395 | 6,992 | 1,803 | 19,117 | 4,641 | 5,843 | 2,972 | 20,042 | 3,795 | 821 | 24,281 |
|  |  |  | QTLR | | 35 | 35 | 35 | 35 | 35 | 35 | 35 | 35 | 35 | 35 | 35 | 35 |
|  |  |  |  | Marker | MD17_01A | MD17_01C | MD17_02A | MD17_02B | MD17_04A | MD17_04B | MD17_05B | MD17_05A | MD17_06A | MD17_06B | MD17_07A | MD17_08A |
| FAM102A | 5,203,358 |  | 35 | MD17_01A |  |  |  |  |  |  |  |  |  |  |  |  |
| FAM102A | 5,230,753 | 27,395 | 35 | MD17_01C | 0.051 |  |  |  |  |  |  |  |  |  |  |  |
| DPM2 | 5,237,745 | 6,992 | 35 | MD17_02A | 0.994 | 0.050 |  |  |  |  |  |  |  |  |  |  |
| DPM2 | 5,239,548 | 1,803 | 35 | MD17_02B | 0.991 | 0.051 | 0.999 |  |  |  |  |  |  |  |  |  |
| ENG | 5,258,665 | 19,117 | 35 | MD17_04A | 0.512 | 0.025 | 0.515 | 0.516 |  |  |  |  |  |  |  |  |
| ENG | 5,263,306 | 4,641 | 35 | MD17_04B | 0.341 | 0.083 | 0.343 | 0.339 | 0.788 |  |  |  |  |  |  |  |
| CDK10 | 5,269,149 | 5,843 | 35 | MD17_05B | 0.053 | 0.980 | 0.052 | 0.052 | 0.027 | 0.084 |  |  |  |  |  |  |
| CDK11 | 5,272,121 | 2,972 | 35 | MD17_05A | 0.001 | 0.364 | 0.000 | 0.000 | 0.025 | 0.225 | 0.371 |  |  |  |  |  |
| SH2D3C | 5,292,163 | 20,042 | 35 | MD17_06A | 0.051 | 0.966 | 0.050 | 0.051 | 0.027 | 0.085 | 0.986 | 0.374 |  |  |  |  |
| SH2D3C | 5,295,958 | 3,795 | 35 | MD17_06B | 0.013 | 0.581 | 0.013 | 0.014 | 0.000 | 0.143 | 0.595 | 0.619 | 0.604 |  |  |  |
| TOR2A | 5,296,779 | 821 | 35 | MD17_07A | 0.032 | 0.001 | 0.032 | 0.032 | 0.062 | 0.050 | 0.000 | 0.215 | 0.001 | 0.350 |  |  |
| URM1 | 5,321,060 | 24,281 | 35 | MD17_08A | 0.018 | 0.540 | 0.018 | 0.019 | 0.002 | 0.125 | 0.553 | 0.584 | 0.563 | 0.937 | 0.335 |  |
|  | | | | P: | 7.75E-01 | 2.59E-01 | 6.92E-01 | 5.18E-01 | 3.96E-01 | 1.53E-01 | 2.18E-01 | 7.79E-01 | 2.24E-01 | 4.35E-01 | 7.07E-01 | 5.13E-01 |
|  |  |  |  | \|α\|: | 0.1844 | 1.3062 | 0.2560 | 0.4173 | 0.5967 | 0.9422 | 1.4302 | 0.2523 | 1.4170 | 0.7775 | 0.5665 | 0.6452 |
|  |  |  |  | cP: | 0.0001 | 0.0005 | 0.0001 | 0.0003 | 0.0005 | 0.0013 | 0.0006 | 0.0000 | 0.0006 | 0.0003 | 0.0001 | 0.0002 |
|  |  |  |  | cG: | 0.0004 | 0.0039 | 0.0008 | 0.0021 | 0.0038 | 0.0101 | 0.0047 | 0.0003 | 0.0046 | 0.0022 | 0.0005 | 0.0015 |

| **Chr 1** | | | | | | | | | | | |
| --- | --- | --- | --- | --- | --- | --- | --- | --- | --- | --- | --- |
| Element | | | | | RASSF8 | RASSF8 | RASSF8 | SSPN | SSPN | ITPR2 | ITPR2 |
|  | bp | | | | 67,554,921 | 67,564,586 | 67,569,296 | 67,629,704 | 67,629,779 | 67,659,080 | 67,810,544 |
|  |  | Dist. | | |  | 9,665 | 4,710 | 60,408 | 75 | 29,301 | 151,464 |
|  |  |  | QTLR | | 4 | 4 | 4 | 4 | 4 | 4 | 4 |
|  |  |  |  | Marker | MD01_03A | MD01_03B | MD01_03C | MD01_04B | MD01_04C | MD01_05C | MD01_05B |
| RASSF8 | 67,554,921 |  | 4 | MD01_03A |  |  |  |  |  |  |  |
| RASSF8 | 67,564,586 | 9,665 | 4 | MD01_03B | 0.315 |  |  |  |  |  |  |
| RASSF8 | 67,569,296 | 4,710 | 4 | MD01_03C | 0.009 | 0.027 |  |  |  |  |  |
| SSPN | 67,629,704 | 60,408 | 4 | MD01_04B | 0.575 | 0.027 | 0.014 |  |  |  |  |
| SSPN | 67,629,779 | 75 | 4 | MD01_04C | 0.575 | 0.027 | 0.012 | 1.000 |  |  |  |
| ITPR2 | 67,659,080 | 29,301 | 4 | MD01_05C | 0.953 | 0.309 | 0.008 | 0.583 | 0.583 |  |  |
| ITPR2 | 67,810,544 | 151,464 | 4 | MD01_05B | 0.863 | 0.264 | 0.008 | 0.532 | 0.532 | 0.909 |  |
|  | | | | P: | 4.24E-01 | 2.40E-01 | 1.37E-01 | 3.72E-01 | 3.72E-01 | 5.10E-01 | 8.16E-01 |
|  |  |  |  | \|α\|: | 0.3744 | 0.5796 | 2.5438 | 0.4088 | 0.4088 | 0.3062 | 0.1098 |
|  |  |  |  | cP: | 0.0001 | 0.0003 | 0.0003 | 0.0002 | 0.0002 | 0.0001 | 0.0000 |
|  |  |  |  | cG: | 0.0011 | 0.0023 | 0.0025 | 0.0013 | 0.0013 | 0.0007 | 0.0001 |

| **Chr 4** | | | | | | | | | | |
| --- | --- | --- | --- | --- | --- | --- | --- | --- | --- | --- |
| Element | | | | | CCDC111 | CCDC111 | CCDC111 | CASP3 | CASP3 | CASP3 |
|  | bp | | | | 39,481,749 | 39,483,799 | 39,486,747 | 39,498,984 | 39,506,324 | 39,509,637 |
|  |  | Dist. | | |  | 2,050 | 2,948 | 12,237 | 7,340 | 3,313 |
|  |  |  | QTLR | | 17 | 17 | 17 | 17 | 17 | 17 |
|  |  |  |  | Marker | MD04_03C | MD04_03B | MD04_03A | MD04_04A | MD04_04B | MD04_04C |
| CCDC111 | 39,481,749 |  | 17 | MD04_03C |  |  |  |  |  |  |
| CCDC111 | 39,483,799 | 2,050 | 17 | MD04_03B | 0.370 |  |  |  |  |  |
| CCDC111 | 39,486,747 | 2,948 | 17 | MD04_03A | 1.000 | 0.370 |  |  |  |  |
| CASP3 | 39,498,984 | 12,237 | 17 | MD04_04A | 0.000 | 0.000 | 0.000 |  |  |  |
| CASP3 | 39,506,324 | 7,340 | 17 | MD04_04B | 0.132 | 0.255 | 0.132 | 0.001 |  |  |
| CASP3 | 39,509,637 | 3,313 | 17 | MD04_04C | 0.000 | 0.000 | 0.000 | 1.000 | 0.001 |  |
|  | | | | P: | 6.65E-01 | 5.73E-01 | 6.65E-01 | 4.68E-01 | 1.96E-01 | 4.67E-01 |
|  |  |  |  | \|α\|: | 0.2126 | 0.2531 | 0.2126 | 7.5779 | 0.7622 | 7.5779 |
|  |  |  |  | cP: | 0.0000 | 0.0001 | 0.0000 | 0.0001 | 0.0004 | 0.0001 |
|  |  |  |  | cG: | 0.0003 | 0.0005 | 0.0003 | 0.0006 | 0.0033 | 0.0006 |

| **Chr 9** | | | | | | | | | | | | | | | | | | | | |
| --- | --- | --- | --- | --- | --- | --- | --- | --- | --- | --- | --- | --- | --- | --- | --- | --- | --- | --- | --- | --- |
| Element | | | | | DLG1 | DLG1 | GMNC | IL1RAP | gga-mir-1762 | gga-mir-1762 | gga-mir-1762 | gga-mir-1762 | TMEM207 | TMEM207 | EPHB | EPHB | LAMP3 | LAMP3 | LAMP3 | LAMP3 |
|  | bp | | | | 12,646,991 | 12,691,840 | 13,967,979 | 14,024,326 | 14,044,716 | 14,044,717 | 14,044,719 | 14,044,752 | 14,072,014 | 14,073,188 | 16,120,414 | 16,129,963 | 16,528,620 | 16,532,498 | 16,537,751 | 16,540,194 |
|  |  | Dist. | | |  | 44,849 | 1,276,139 | 56,347 | 20,390 | 1 | 2 | 33 | 27,262 | 1,174 | 2,047,226 | 9,549 | 398,657 | 3,878 | 5,253 | 2,443 |
|  |  |  | QTLR | | 28 | 28 | 28 | 28 | 28 | 28 | 28 | 28 | 28 | 28 | 28 | 28 | 28 | 28 | 28 | 28 |
|  |  |  |  | Marker | MD09_01B | MD09_01A | MD09_02B | MD09_04A | MD09_05Da | MD09_05Db | MD09_05C | MD09_05A | MD09_06B | MD09_06C | MD09_07C | MD09_07A | MD09_08A | MD09_08B | MD09_08C | MD09_08D |
| DLG1 | 12,646,991 |  | 28 | MD09_01B |  |  |  |  |  |  |  |  |  |  |  |  |  |  |  |  |
| DLG1 | 12,691,840 | 44,849 | 28 | MD09_01A | 0.000 |  |  |  |  |  |  |  |  |  |  |  |  |  |  |  |
| GMNC | 13,967,979 | 1,276,139 | 28 | MD09_02B | 0.052 | 0.017 |  |  |  |  |  |  |  |  |  |  |  |  |  |  |
| IL1RAP | 14,024,326 | 56,347 | 28 | MD09_04A | 0.000 | 0.000 | 0.052 |  |  |  |  |  |  |  |  |  |  |  |  |  |
| gga-mir-1762 | 14,044,716 | 20,390 | 28 | MD09_05Da | 0.066 | 0.000 | 0.788 | 0.000 |  |  |  |  |  |  |  |  |  |  |  |  |
| gga-mir-1762 | 14,044,717 | 1 | 28 | MD09_05Db | 0.066 | 0.000 | 0.788 | 0.000 | 1.000 |  |  |  |  |  |  |  |  |  |  |  |
| gga-mir-1762 | 14,044,719 | 2 | 28 | MD09_05C | 0.055 | 0.018 | 0.947 | 0.055 | 0.833 | 0.833 |  |  |  |  |  |  |  |  |  |  |
| gga-mir-1762 | 14,044,752 | 33 | 28 | MD09_05A | 0.000 | 0.000 | 0.052 | 1.000 | 0.000 | 0.000 | 0.055 |  |  |  |  |  |  |  |  |  |
| TMEM207 | 14,072,014 | 27,262 | 28 | MD09_06B | 0.000 | 0.000 | 0.000 | 0.000 | 0.000 | 0.000 | 0.000 | 0.000 |  |  |  |  |  |  |  |  |
| TMEM207 | 14,073,188 | 1,174 | 28 | MD09_06C | 0.000 | 0.000 | 0.000 | 0.000 | 0.000 | 0.000 | 0.000 | 0.000 | 1.000 |  |  |  |  |  |  |  |
| EPHB | 16,120,414 | 2,047,226 | 28 | MD09_07C | 0.000 | 0.001 | 0.000 | 0.004 | 0.000 | 0.000 | 0.000 | 0.004 | 0.003 | 0.003 |  |  |  |  |  |  |
| EPHB | 16,129,963 | 9,549 | 28 | MD09_07A | 0.000 | 0.005 | 0.000 | 0.004 | 0.000 | 0.000 | 0.000 | 0.004 | 0.003 | 0.002 | 0.893 |  |  |  |  |  |
| LAMP3 | 16,528,620 | 398,657 | 28 | MD09_08A | 0.000 | 0.000 | 0.001 | 0.000 | 0.001 | 0.001 | 0.001 | 0.000 | 0.005 | 0.005 | 0.486 | 0.525 |  |  |  |  |
| LAMP3 | 16,532,498 | 3,878 | 28 | MD09_08B | 0.000 | 0.000 | 0.000 | 0.000 | 0.000 | 0.000 | 0.000 | 0.000 | 0.002 | 0.002 | 0.000 | 0.058 | 0.033 |  |  |  |
| LAMP3 | 16,537,751 | 5,253 | 28 | MD09_08C | 0.000 | 0.000 | 0.000 | 0.000 | 0.000 | 0.000 | 0.000 | 0.000 | 0.002 | 0.002 | 0.000 | 0.057 | 0.033 | 1.000 |  |  |
| LAMP3 | 16,540,194 | 2,443 | 28 | MD09_08D | 0.000 | 0.000 | 0.000 | 0.000 | 0.000 | 0.000 | 0.000 | 0.000 | 0.002 | 0.002 | 0.000 | 0.057 | 0.033 | 1.000 | 1.000 |  |
|  | | | | P: | 6.96E-01 | 2.50E-01 | 4.36E-01 | 6.86E-01 | 5.55E-01 | 5.55E-01 | 5.00E-01 | 6.86E-01 | 7.88E-01 | 8.48E-01 | 3.95E-01 | 2.41E-01 | 1.37E-01 | 5.00E-01 | 5.41E-01 | 2.01E-01 |
|  |  |  |  | \|α\|: | 4.0743 | 4.3265 | 2.2028 | 4.2164 | 1.8063 | 1.8063 | 1.9784 | 4.2164 | 0.3212 | 0.2323 | 0.7663 | 0.9883 | 1.0003 | 1.8593 | 1.7001 | 3.1368 |
|  |  |  |  | cP: | 0.0000 | 0.0003 | 0.0001 | 0.0000 | 0.0001 | 0.0001 | 0.0001 | 0.0000 | 0.0000 | 0.0000 | 0.0002 | 0.0003 | 0.0005 | 0.0001 | 0.0001 | 0.0003 |
|  |  |  |  | cG: | 0.0002 | 0.0021 | 0.0009 | 0.0002 | 0.0005 | 0.0005 | 0.0007 | 0.0002 | 0.0001 | 0.0001 | 0.0013 | 0.0023 | 0.0037 | 0.0006 | 0.0005 | 0.0020 |

| **Chr 13** | | | | | | |
| --- | --- | --- | --- | --- | --- | --- |
| Element | | | | | SLIT3 | SLIT3 |
|  | bp | | | | 5,324,164 | 5,421,966 |
|  |  | Dist. | | |  | 97,802 |
|  |  |  | QTLR | | 31 | 31 |
|  |  |  |  | Marker | MD13_03B | MD13_03C |
| SLIT3 | 5,324,164 |  | 31 | MD13_03B |  |  |
| SLIT3 | 5,421,966 | 97,802 | 31 | MD13_03C | 0.230 |  |
|  | | | | P: | 7.96E-01 | 9.76E-01 |
|  |  |  |  | \|α\|: | 0.1294 | 0.0144 |
|  |  |  |  | cP: | 0.0000 | 0.0000 |
|  |  |  |  | cG: | 0.0001 | 0.0000 |

| **Chr 14** | | | | | | | | |
| --- | --- | --- | --- | --- | --- | --- | --- | --- |
| Element | | | | | HN1L | CRAMP1 | TMEM204 | TMEM204 |
|  | bp | | | | 14,030,671 | 14,050,330 | 14,115,781 | 14,142,117 |
|  |  | Dist. | | |  | 19,659 | 65,451 | 26,336 |
|  |  |  | QTLR | | 34 | 34 | 34 | 34 |
|  |  |  |  | Marker | MD14_01A | MD14_02A | MD14_03B | MD14_03A |
| HN1L | 14,030,671 |  | 34 | MD14_01A |  |  |  |  |
| CRAMP1 | 14,050,330 | 19,659 | 34 | MD14_02A | 0.599 |  |  |  |
| TMEM204 | 14,115,781 | 65,451 | 34 | MD14_03B | 1.000 | 0.599 |  |  |
| TMEM204 | 14,142,117 | 26,336 | 34 | MD14_03A | 0.617 | 0.968 | 0.617 |  |
|  | | | | P: | 4.51E-01 | 9.60E-01 | 4.51E-01 | 7.54E-01 |
|  |  |  |  | \|α\|: | 0.3580 | 0.0251 | 0.3580 | 0.1558 |
|  |  |  |  | cP: | 0.0001 | 0.0000 | 0.0001 | 0.0000 |
|  |  |  |  | cG: | 0.0010 | 0.0000 | 0.0010 | 0.0002 |

| **Chr 17** | | | | | | | | | | | | | | | | | |
| --- | --- | --- | --- | --- | --- | --- | --- | --- | --- | --- | --- | --- | --- | --- | --- | --- | --- |
| Element | | | | | FAM102A | FAM102A | DPM2 | DPM2 | ST6GALNAC6 | ENG | ENG | CDK10 | CDK11 | SH2D3C | SH2D3C | TOR2A | URM1 |
|  | bp | | | | 5,203,358 | 5,230,753 | 5,237,745 | 5,239,548 | 5,245,311 | 5,258,665 | 5,263,306 | 5,269,149 | 5,272,121 | 5,292,163 | 5,295,958 | 5,296,779 | 5,321,060 |
|  |  | Dist. | | |  | 27,395 | 6,992 | 1,803 | 5,763 | 13,354 | 4,641 | 5,843 | 2,972 | 20,042 | 3,795 | 821 | 24,281 |
|  |  |  | QTLR | | 35 | 35 | 35 | 35 | 35 | 35 | 35 | 35 | 35 | 35 | 35 | 35 | 35 |
|  |  |  |  | Marker | MD17_01A | MD17_01C | MD17_02A | MD17_02B | MD17_03A | MD17_04A | MD17_04B | MD17_05B | MD17_05A | MD17_06A | MD17_06B | MD17_07A | MD17_08A |
| FAM102A | 5,203,358 |  | 35 | MD17_01A |  |  |  |  |  |  |  |  |  |  |  |  |  |
| FAM102A | 5,230,753 | 27,395 | 35 | MD17_01C | 0.975 |  |  |  |  |  |  |  |  |  |  |  |  |
| DPM2 | 5,237,745 | 6,992 | 35 | MD17_02A | 0.968 | 0.987 |  |  |  |  |  |  |  |  |  |  |  |
| DPM2 | 5,239,548 | 1,803 | 35 | MD17_02B | 0.966 | 0.985 | 0.997 |  |  |  |  |  |  |  |  |  |  |
| ST6GALNAC6 | 5,245,311 | 5,763 | 35 | MD17_03A | 0.002 | 0.009 | 0.009 | 0.009 |  |  |  |  |  |  |  |  |  |
| ENG | 5,258,665 | 13,354 | 35 | MD17_04A | 0.645 | 0.632 | 0.645 | 0.644 | 0.001 |  |  |  |  |  |  |  |  |
| ENG | 5,263,306 | 4,641 | 35 | MD17_04B | 0.000 | 0.000 | 0.000 | 0.000 | 0.000 | 0.000 |  |  |  |  |  |  |  |
| CDK10 | 5,269,149 | 5,843 | 35 | MD17_05B | 0.552 | 0.562 | 0.564 | 0.567 | 0.005 | 0.220 | 0.000 |  |  |  |  |  |  |
| CDK11 | 5,272,121 | 2,972 | 35 | MD17_05A | 0.167 | 0.187 | 0.190 | 0.192 | 0.025 | 0.026 | 0.000 | 0.329 |  |  |  |  |  |
| SH2D3C | 5,292,163 | 20,042 | 35 | MD17_06A | 0.181 | 0.176 | 0.178 | 0.181 | 0.000 | 0.025 | 0.000 | 0.312 | 0.941 |  |  |  |  |
| SH2D3C | 5,295,958 | 3,795 | 35 | MD17_06B | 0.162 | 0.182 | 0.185 | 0.187 | 0.025 | 0.026 | 0.000 | 0.320 | 0.979 | 0.953 |  |  |  |
| TOR2A | 5,296,779 | 821 | 35 | MD17_07A | 0.177 | 0.173 | 0.176 | 0.178 | 0.000 | 0.026 | 0.000 | 0.309 | 0.932 | 0.991 | 0.953 |  |  |
| URM1 | 5,321,060 | 24,281 | 35 | MD17_08A | 0.171 | 0.169 | 0.169 | 0.173 | 0.000 | 0.025 | 0.000 | 0.298 | 0.899 | 0.956 | 0.916 | 0.956 |  |
|  | | | | P: | 1.10E-01 | 1.30E-01 | 1.63E-01 | 1.48E-01 | 4.00E-01 | 8.15E-02 | 1.29E-01 | 2.10E-01 | 5.97E-01 | 9.09E-01 | 6.85E-01 | 9.52E-01 | 9.39E-01 |
|  |  |  |  | \|α\|: | 0.7888 | 0.7470 | 0.6942 | 0.7180 | 2.7118 | 0.9600 | 7.9095 | 0.7121 | 0.4266 | 0.0948 | 0.3285 | 0.0492 | 0.0632 |
|  |  |  |  | cP: | 0.0006 | 0.0005 | 0.0004 | 0.0005 | 0.0001 | 0.0007 | 0.0002 | 0.0003 | 0.0001 | 0.0000 | 0.0000 | 0.0000 | 0.0000 |
|  |  |  |  | cG: | 0.0043 | 0.0039 | 0.0033 | 0.0036 | 0.0010 | 0.0052 | 0.0013 | 0.0027 | 0.0004 | 0.0000 | 0.0003 | 0.0000 | 0.0000 |

| **Chr 1** | | | | | | | | | | | | |
| --- | --- | --- | --- | --- | --- | --- | --- | --- | --- | --- | --- | --- |
| Element | | | | | TRHDE | TRHDE | RASSF8 | RASSF8 | SSPN | SSPN | ITPR2 | ITPR2 |
|  | bp | | | | 36,839,853 | 36,951,385 | 67,564,586 | 67,569,296 | 67,629,704 | 67,629,779 | 67,810,544 | 67,882,133 |
|  |  | Dist. | | |  | 111,532 | 30,613,201 | 4,710 | 60,408 | 75 | 180,765 | 71,589 |
|  |  |  | QTLR | | 2 | 2 | 4 | 4 | 4 | 4 | 4 | 4 |
|  |  |  |  | Marker | MD01_02B | MD01_02D | MD01_03B | MD01_03C | MD01_04B | MD01_04C | MD01_05B | MD01_05A |
| TRHDE | 36,839,853 |  | 2 | MD01_02B |  |  |  |  |  |  |  |  |
| TRHDE | 36,951,385 | 111,532 | 2 | MD01_02D | 0.067 |  |  |  |  |  |  |  |
| RASSF8 | 67,564,586 | 30,613,201 | 4 | MD01_03B | 0.000 | 0.001 |  |  |  |  |  |  |
| RASSF8 | 67,569,296 | 4,710 | 4 | MD01_03C | 0.000 | 0.001 | 1.000 |  |  |  |  |  |
| SSPN | 67,629,704 | 60,408 | 4 | MD01_04B | 0.004 | 0.004 | 0.392 | 0.392 |  |  |  |  |
| SSPN | 67,629,779 | 75 | 4 | MD01_04C | 0.004 | 0.004 | 0.392 | 0.392 | 1.000 |  |  |  |
| ITPR2 | 67,810,544 | 180,765 | 4 | MD01_05B | 0.002 | 0.000 | 0.001 | 0.001 | 0.003 | 0.003 |  |  |
| ITPR2 | 67882133 | 71589 | 4 | MD01_05A | 0.000 | 0.000 | 0.658 | 0.658 | 0.245 | 0.245 | 0.001 |  |
|  | | | | P: | 3.81E-01 | 8.81E-01 | 6.13E-01 | 6.13E-01 | 7.90E-01 | 7.90E-01 | 5.95E-01 | 3.90E-01 |
|  |  |  |  | \|α\|: | 0.8313 | 0.0946 | 0.3758 | 0.3758 | 0.2919 | 0.2919 | 6.4520 | 0.6511 |
|  |  |  |  | cP: | 0.0006 | 0.0000 | 0.0002 | 0.0002 | 0.0001 | 0.0001 | 0.0001 | 0.0005 |
|  |  |  |  | cG: | 0.0043 | 0.0001 | 0.0014 | 0.0014 | 0.0004 | 0.0004 | 0.0008 | 0.0037 |

| **Chr 4** | | | | | | | | | | | |
| --- | --- | --- | --- | --- | --- | --- | --- | --- | --- | --- | --- |
| Element | | | | | ACSL1 | CCDC111 | CCDC111 | CCDC111 | CASP3 | CASP3 | CASP3 |
|  | bp | | | | 39,453,244 | 39,481,749 | 39,483,799 | 39,486,747 | 39,498,984 | 39,506,324 | 39,509,637 |
|  |  | Dist. | | |  | 28,505 | 2,050 | 2,948 | 12,237 | 7,340 | 3,313 |
|  |  |  | QTLR | | 17 | 17 | 17 | 17 | 17 | 17 | 17 |
|  |  |  |  | Marker | MD04_02A | MD04_03C | MD04_03B | MD04_03A | MD04_04A | MD04_04B | MD04_04C |
| ACSL1 | 39,453,244 |  | 17 | MD04_02A |  |  |  |  |  |  |  |
| CCDC111 | 39,481,749 | 28,505 | 17 | MD04_03C | 0.688 |  |  |  |  |  |  |
| CCDC111 | 39,483,799 | 2,050 | 17 | MD04_03B | 1.000 | 0.690 |  |  |  |  |  |
| CCDC111 | 39,486,747 | 2,948 | 17 | MD04_03A | 0.664 | 0.981 | 0.665 |  |  |  |  |
| CASP3 | 39,498,984 | 12,237 | 17 | MD04_04A | 0.540 | 0.373 | 0.539 | 0.376 |  |  |  |
| CASP3 | 39,506,324 | 7,340 | 17 | MD04_04B | 0.900 | 0.624 | 0.900 | 0.635 | 0.593 |  |  |
| CASP3 | 39,509,637 | 3,313 | 17 | MD04_04C | 0.541 | 0.375 | 0.541 | 0.380 | 0.997 | 0.596 |  |
|  | | | | P: | 2.38E-01 | 7.28E-01 | 2.24E-01 | 9.65E-01 | 1.41E-01 | 3.26E-01 | 1.20E-01 |
|  |  |  |  | \|α\|: | 0.8935 | 0.2453 | 0.9184 | 0.0311 | 1.4444 | 0.7747 | 1.5212 |
|  |  |  |  | cP: | 0.0011 | 0.0001 | 0.0012 | 0.0000 | 0.0021 | 0.0008 | 0.0024 |
|  |  |  |  | cG: | 0.0088 | 0.0008 | 0.0093 | 0.0000 | 0.0164 | 0.0063 | 0.0182 |

| **Chr 9** | | | | | | | | | | | | | | | | |
| --- | --- | --- | --- | --- | --- | --- | --- | --- | --- | --- | --- | --- | --- | --- | --- | --- |
| Element | | | | | DLG1 | DLG1 | GMNC | intergenic | IL1RAP | gga-mir-1762 | gga-mir-1762 | TMEM207 | TMEM207 | TMEM207 | LAMP3 | LAMP3 |
|  | bp | | | | 12,646,991 | 12,691,840 | 13,967,979 | 13,991,344 | 14,024,326 | 14,044,717 | 14,044,752 | 14,069,448 | 14,072,014 | 14,073,188 | 16,532,498 | 16,537,751 |
|  |  | Dist. | | |  | 44,849 | 1,276,139 | 23,365 | 32,982 | 20,391 | 35 | 24,696 | 2,566 | 1,174 | 2,459,310 | 5,253 |
|  |  |  | QTLR | | 28 | 28 | 28 | 28 | 28 | 28 | 28 | 28 | 28 | 28 | 28 | 28 |
|  |  |  |  | Marker | MD09_01B | MD09_01A | MD09_02B | MD09_03A | MD09_04A | MD09_05Db | MD09_05A | MD09_06A | MD09_06B | MD09_06C | MD09_08B | MD09_08C |
| DLG1 | 12,646,991 |  | 28 | MD09_01B |  |  |  |  |  |  |  |  |  |  |  |  |
| DLG1 | 12,691,840 | 44,849 | 28 | MD09_01A | 0.760 |  |  |  |  |  |  |  |  |  |  |  |
| GMNC | 13,967,979 | 1,276,139 | 28 | MD09_02B | 0.045 | 0.059 |  |  |  |  |  |  |  |  |  |  |
| intergenic | 13,991,344 | 23,365 | 28 | MD09_03A | 0.000 | 0.004 | 0.771 |  |  |  |  |  |  |  |  |  |
| IL1RAP | 14,024,326 | 32,982 | 28 | MD09_04A | 0.248 | 0.179 | 0.022 | 0.115 |  |  |  |  |  |  |  |  |
| gga-mir-1762 | 14,044,717 | 20,391 | 28 | MD09_05Db | 0.011 | 0.001 | 0.324 | 0.578 | 0.185 |  |  |  |  |  |  |  |
| gga-mir-1762 | 14,044,752 | 35 | 28 | MD09_05A | 0.074 | 0.033 | 0.256 | 0.464 | 0.156 | 0.852 |  |  |  |  |  |  |
| TMEM207 | 14,069,448 | 24,696 | 28 | MD09_06A | 0.000 | 0.004 | 0.748 | 0.963 | 0.110 | 0.601 | 0.484 |  |  |  |  |  |
| TMEM207 | 14,072,014 | 2,566 | 28 | MD09_06B | 0.000 | 0.004 | 0.748 | 0.963 | 0.110 | 0.599 | 0.482 | 1.000 |  |  |  |  |
| TMEM207 | 14,073,188 | 1,174 | 28 | MD09_06C | 0.032 | 0.007 | 0.653 | 0.843 | 0.098 | 0.531 | 0.623 | 0.876 | 0.876 |  |  |  |
| LAMP3 | 16,532,498 | 2,459,310 | 28 | MD09_08B | 0.000 | 0.002 | 0.023 | 0.030 | 0.005 | 0.021 | 0.026 | 0.034 | 0.034 | 0.040 |  |  |
| LAMP3 | 16,537,751 | 5,253 | 28 | MD09_08C | 0.000 | 0.002 | 0.023 | 0.030 | 0.005 | 0.021 | 0.026 | 0.034 | 0.035 | 0.040 | 1.000 |  |
|  | | | | P: | 3.51E-01 | 4.26E-01 | 3.27E-03 | 2.01E-04 | 9.12E-02 | 3.54E-04 | 1.71E-03 | 1.14E-03 | 1.16E-03 | 3.01E-03 | 6.56E-01 | 6.56E-01 |
|  |  |  |  | \|a\|: | 0.8509 | 0.6570 | 2.0800 | 2.4741 | 2.2320 | 2.6022 | 2.2098 | 2.1642 | 2.1597 | 1.9440 | 0.5311 | 0.5311 |
|  |  |  |  | cP: | 0.0005 | 0.0004 | 0.0061 | 0.0095 | 0.0018 | 0.0086 | 0.0067 | 0.0074 | 0.0073 | 0.0062 | 0.0001 | 0.0001 |
|  |  |  |  | cG: | 0.0040 | 0.0029 | 0.0470 | 0.0733 | 0.0137 | 0.0659 | 0.0515 | 0.0566 | 0.0564 | 0.0475 | 0.0011 | 0.0011 |

| **Chr 13** | | | | | | |
| --- | --- | --- | --- | --- | --- | --- |
| Element | | | | | SLIT3 | SLIT3 |
|  | bp | | | | 4,954,745 | 5,324,164 |
|  |  | Dist. | | |  | 369,419 |
|  |  |  | QTLR | | 31 | 31 |
|  |  |  |  | Marker | MD13_03A | MD13_03B |
| SLIT3 | 4,954,745 |  | 31 | MD13_03A |  |  |
| SLIT3 | 5,324,164 | 369,419 | 31 | MD13_03B | 0.136 |  |
|  | | | | P: | 4.11E-01 | 8.25E-01 |
|  |  |  |  | \|α\|: | 0.5384 | 0.1656 |
|  |  |  |  | cP: | 0.0004 | 0.0000 |
|  |  |  |  | cG: | 0.0034 | 0.0003 |

| **Chr 14** | | | | | | | | |
| --- | --- | --- | --- | --- | --- | --- | --- | --- |
| Element | | | | | HN1L | TMEM204 | TMEM204 | ATP6V0C |
|  | bp | | | | 14,030,671 | 14,115,781 | 14,142,117 | 14,252,012 |
|  |  | Dist. | | |  | 85,110 | 26,336 | 109,895 |
|  |  |  | QTLR | | 34 | 34 | 34 | 34 |
|  |  |  |  | Marker | MD14_01A | MD14_03B | MD14_03A | MD14_04A |
| HN1L | 14,030,671 |  | 34 | MD14_01A |  |  |  |  |
| TMEM204 | 14,115,781 | 85,110 | 34 | MD14_03B | 1.000 |  |  |  |
| TMEM204 | 14,142,117 | 26,336 | 34 | MD14_03A | 0.424 | 0.424 |  |  |
| ATP6V0C | 14,252,012 | 109,895 | 34 | MD14_04A | 0.002 | 0.002 | 0.004 |  |
|  | | | | P: | 9.11E-01 | 9.11E-01 | 3.72E-01 | 3.32E-01 |
|  |  |  |  | \|α\|: | 0.0717 | 0.0717 | 0.6594 | 0.6598 |
|  |  |  |  | cP: | 0.0000 | 0.0000 | 0.0005 | 0.0006 |
|  |  |  |  | cG: | 0.0001 | 0.0001 | 0.0041 | 0.0045 |

| **Chr 17** | | | | | | | | | | | | | | | | | | | | |
| --- | --- | --- | --- | --- | --- | --- | --- | --- | --- | --- | --- | --- | --- | --- | --- | --- | --- | --- | --- | --- |
| Element | | | | | FAM102A | FAM102A | FAM102A | DPM2 | DPM2 | ST6GALNAC6 | ST6GALNAC6 | ST6GALNAC6 | ENG | CDK10 | CDK11 | SH2D3C | SH2D3C | TOR2A | TOR2A | URM1 |
|  | bp | | | | 5,203,358 | 5,221,308 | 5,230,753 | 5,237,745 | 5,239,548 | 5,245,311 | 5,246,277 | 5,247,038 | 5,263,306 | 5,269,149 | 5,272,121 | 5,292,163 | 5,295,958 | 5,296,779 | 5,298,419 | 5,321,060 |
|  |  | Dist. | | |  | 17,950 | 9,445 | 6,992 | 1,803 | 5,763 | 966 | 761 | 16,268 | 5,843 | 2,972 | 20,042 | 3,795 | 821 | 1,640 | 22,641 |
|  |  |  | QTLR | | 35 | 35 | 35 | 35 | 35 | 35 | 35 | 35 | 35 | 35 | 35 | 35 | 35 | 35 | 35 | 35 |
|  |  |  |  | Marker | MD17_01A | MD17_01B | MD17_01C | MD17_02A | MD17_02B | MD17_03A | MD17_03B | MD17_03C | MD17_04B | MD17_05B | MD17_05A | MD17_06A | MD17_06B | MD17_07A | MD17_07B | MD17_08A |
| FAM102A | 5,203,358 |  | 35 | MD17_01A |  |  |  |  |  |  |  |  |  |  |  |  |  |  |  |  |
| FAM102A | 5,221,308 | 17,950 | 35 | MD17_01B | 0.161 |  |  |  |  |  |  |  |  |  |  |  |  |  |  |  |
| FAM102A | 5,230,753 | 9,445 | 35 | MD17_01C | 0.173 | 0.968 |  |  |  |  |  |  |  |  |  |  |  |  |  |  |
| DPM2 | 5,237,745 | 6,992 | 35 | MD17_02A | 0.345 | 0.717 | 0.741 |  |  |  |  |  |  |  |  |  |  |  |  |  |
| DPM2 | 5,239,548 | 1,803 | 35 | MD17_02B | 0.197 | 0.927 | 0.954 | 0.692 |  |  |  |  |  |  |  |  |  |  |  |  |
| ST6GALNAC6 | 5,245,311 | 5,763 | 35 | MD17_03A | 0.711 | 0.386 | 0.384 | 0.284 | 0.367 |  |  |  |  |  |  |  |  |  |  |  |
| ST6GALNAC6 | 5,246,277 | 966 | 35 | MD17_03B | 0.711 | 0.388 | 0.383 | 0.284 | 0.368 | 0.998 |  |  |  |  |  |  |  |  |  |  |
| ST6GALNAC6 | 5,247,038 | 761 | 35 | MD17_03C | 0.709 | 0.390 | 0.386 | 0.285 | 0.370 | 0.997 | 0.998 |  |  |  |  |  |  |  |  |  |
| ENG | 5,263,306 | 16,268 | 35 | MD17_04B | 0.081 | 0.155 | 0.140 | 0.005 | 0.106 | 0.062 | 0.062 | 0.062 |  |  |  |  |  |  |  |  |
| CDK10 | 5,269,149 | 5,843 | 35 | MD17_05B | 0.063 | 0.125 | 0.129 | 0.020 | 0.134 | 0.050 | 0.050 | 0.050 | 0.796 |  |  |  |  |  |  |  |
| CDK11 | 5,272,121 | 2,972 | 35 | MD17_05A | 0.344 | 0.667 | 0.659 | 0.893 | 0.688 | 0.265 | 0.267 | 0.269 | 0.018 | 0.033 |  |  |  |  |  |  |
| SH2D3C | 5,292,163 | 20,042 | 35 | MD17_06A | 0.965 | 0.143 | 0.156 | 0.320 | 0.179 | 0.688 | 0.687 | 0.685 | 0.089 | 0.076 | 0.345 |  |  |  |  |  |
| SH2D3C | 5,295,958 | 3,795 | 35 | MD17_06B | 0.273 | 0.131 | 0.133 | 0.093 | 0.128 | 0.200 | 0.201 | 0.201 | 0.017 | 0.015 | 0.095 | 0.270 |  |  |  |  |
| TOR2A | 5,296,779 | 821 | 35 | MD17_07A | 0.271 | 0.149 | 0.146 | 0.100 | 0.139 | 0.196 | 0.196 | 0.196 | 0.029 | 0.024 | 0.097 | 0.281 | 0.970 |  |  |  |
| TOR2A | 5,298,419 | 1,640 | 35 | MD17_07B | 0.305 | 0.539 | 0.557 | 0.757 | 0.582 | 0.219 | 0.219 | 0.219 | 0.021 | 0.020 | 0.785 | 0.317 | 0.107 | 0.105 |  |  |
| URM1 | 5,321,060 | 22,641 | 35 | MD17_08A | 0.000 | 0.012 | 0.005 | 0.005 | 0.004 | 0.005 | 0.005 | 0.005 | 0.053 | 0.017 | 0.004 | 0.008 | 0.000 | 0.002 | 0.000 |  |
|  | | | | P: | 4.36E-01 | 9.74E-01 | 8.82E-01 | 7.40E-01 | 9.61E-01 | 6.05E-01 | 5.98E-01 | 6.49E-01 | 6.21E-01 | 3.48E-01 | 6.97E-01 | 3.59E-01 | 7.80E-01 | 7.19E-01 | 3.08E-01 | 1.71E-01 |
|  |  |  |  | \|α\|: | 0.4960 | 0.0204 | 0.0950 | 0.2154 | 0.0312 | 0.3365 | 0.3431 | 0.2983 | 0.5651 | 1.0858 | 0.2558 | 0.5829 | 0.2030 | 0.2612 | 0.6984 | 6.8243 |
|  |  |  |  | cP: | 0.0004 | 0.0000 | 0.0000 | 0.0001 | 0.0000 | 0.0002 | 0.0002 | 0.0001 | 0.0002 | 0.0006 | 0.0001 | 0.0006 | 0.0001 | 0.0001 | 0.0007 | 0.0024 |
|  |  |  |  | cG: | 0.0033 | 0.0000 | 0.0001 | 0.0005 | 0.0000 | 0.0015 | 0.0015 | 0.0012 | 0.0014 | 0.0043 | 0.0007 | 0.0046 | 0.0004 | 0.0007 | 0.0050 | 0.0182 |

| **Chr 1** | | | | | | | | | | | |
| --- | --- | --- | --- | --- | --- | --- | --- | --- | --- | --- | --- |
| Element | | | | | TRHDE | TRHDE | RASSF8 | RASSF8 | SSPN | SSPN | ITPR2 |
|  | bp | | | | 36,839,853 | 36,951,385 | 67,564,586 | 67,569,296 | 67,629,704 | 67,629,779 | 67,882,133 |
|  |  | Dist. | | |  | 111,532 | 30,613,201 | 4,710 | 60,408 | 75 | 252,354 |
|  |  |  | QTLR | | 2 | 2 | 4 | 4 | 4 | 4 | 4 |
|  |  |  |  | Marker | MD01_02B | MD01_02D | MD01_03B | MD01_03C | MD01_04B | MD01_04C | MD01_05A |
| TRHDE | 36,839,853 |  | 2 | MD01_02B |  |  |  |  |  |  |  |
| TRHDE | 36,951,385 | 111,532 | 2 | MD01_02D | 0.305 |  |  |  |  |  |  |
| RASSF8 | 67,564,586 | 30,613,201 | 4 | MD01_03B | 0.000 | 0.009 |  |  |  |  |  |
| RASSF8 | 67,569,296 | 4,710 | 4 | MD01_03C | 0.000 | 0.009 | 1.000 |  |  |  |  |
| SSPN | 67,629,704 | 60,408 | 4 | MD01_04B | 0.001 | 0.003 | 0.006 | 0.006 |  |  |  |
| SSPN | 67,629,779 | 75 | 4 | MD01_04C | 0.001 | 0.002 | 0.005 | 0.005 | 0.832 |  |  |
| ITPR2 | 67,882,133 | 252,354 | 4 | MD01_05A | 0.000 | 0.009 | 0.788 | 0.788 | 0.001 | 0.000 |  |
|  | | | | P: | 8.36E-01 | 1.86E-01 | 1.88E-01 | 1.88E-01 | 7.39E-01 | 6.74E-01 | 1.05E-01 |
|  |  |  |  | \|α\|: | 0.1453 | 0.8808 | 0.8849 | 0.8849 | 1.1211 | 1.4038 | 1.0842 |
|  |  |  |  | cP: | 0.0000 | 0.0013 | 0.0013 | 0.0013 | 0.0001 | 0.0001 | 0.0020 |
|  |  |  |  | cG: | 0.0003 | 0.0097 | 0.0103 | 0.0103 | 0.0005 | 0.0007 | 0.0156 |

| **Chr 4** | | | | | | | | | | | |
| --- | --- | --- | --- | --- | --- | --- | --- | --- | --- | --- | --- |
| Element | | | | | ACSL1 | CCDC111 | CCDC111 | CCDC111 | CASP3 | CASP3 | CASP3 |
|  | bp | | | | 39,453,244 | 39,481,749 | 39,483,799 | 39,486,747 | 39,498,984 | 39,506,324 | 39,509,637 |
|  |  | Dist. | | |  | 28,505 | 2,050 | 2,948 | 12,237 | 7,340 | 3,313 |
|  |  |  | QTLR | | 17 | 17 | 17 | 17 | 17 | 17 | 17 |
|  |  |  |  | Marker | MD04_02A | MD04_03C | MD04_03B | MD04_03A | MD04_04A | MD04_04B | MD04_04C |
| ACSL1 | 39,453,244 |  | 17 | MD04_02A |  |  |  |  |  |  |  |
| CCDC111 | 39,481,749 | 28,505 | 17 | MD04_03C | 0.992 |  |  |  |  |  |  |
| CCDC111 | 39,483,799 | 2,050 | 17 | MD04_03B | 0.069 | 0.073 |  |  |  |  |  |
| CCDC111 | 39,486,747 | 2,948 | 17 | MD04_03A | 0.073 | 0.073 | 0.931 |  |  |  |  |
| CASP3 | 39,498,984 | 12,237 | 17 | MD04_04A | 0.955 | 0.960 | 0.073 | 0.075 |  |  |  |
| CASP3 | 39,506,324 | 7,340 | 17 | MD04_04B | 0.952 | 0.960 | 0.075 | 0.075 | 1.000 |  |  |
| CASP3 | 39,509,637 | 3,313 | 17 | MD04_04C | 0.952 | 0.960 | 0.075 | 0.075 | 1.000 | 1.000 |  |
|  | | | | P: | 8.52E-01 | 6.35E-01 | 8.93E-01 | 8.79E-01 | 7.01E-01 | 6.75E-01 | 6.75E-01 |
|  |  |  |  | \|α\|: | 0.1609 | 0.4092 | 0.2900 | 0.3528 | 0.3375 | 0.3691 | 0.3691 |
|  |  |  |  | cP: | 0.0000 | 0.0002 | 0.0000 | 0.0000 | 0.0001 | 0.0001 | 0.0001 |
|  |  |  |  | cG: | 0.0002 | 0.0012 | 0.0001 | 0.0001 | 0.0008 | 0.0010 | 0.0010 |

| **Chr 9** | | | | | | | | | | |
| --- | --- | --- | --- | --- | --- | --- | --- | --- | --- | --- |
| Element | | | | | DLG1 | DLG1 | intergenic | gga-mir-1762 | LAMP3 | LAMP3 |
|  | bp | | | | 12,646,991 | 12,691,840 | 13,991,344 | 14,044,752 | 16,532,498 | 16,537,751 |
|  |  | Dist. | | |  | 44,849 | 1,299,504 | 53,408 | 2,487,746 | 5,253 |
|  |  |  | QTLR | | 28 | 28 | 28 | 28 | 28 | 28 |
|  |  |  |  | Marker | MD09_01B | MD09_01A | MD09_03A | MD09_05A | MD09_08B | MD09_08C |
| DLG1 | 12,646,991 |  | 28 | MD09_01B |  |  |  |  |  |  |
| DLG1 | 12,691,840 | 44,849 | 28 | MD09_01A | 0.029 |  |  |  |  |  |
| intergenic | 13,991,344 | 1299504 | 28 | MD09_03A | 0.000 | 0.002 |  |  |  |  |
| gga-mir-1762 | 14,044,752 | 53,408 | 28 | MD09_05A | 0.000 | 0.002 | 0.000 |  |  |  |
| LAMP3 | 16,532,498 | 2487746 | 28 | MD09_08B | 0.001 | 0.006 | 0.000 | 0.003 |  |  |
| LAMP3 | 16,537,751 | 5,253 | 28 | MD09_08C | 0.001 | 0.006 | 0.000 | 0.003 | 1.000 |  |
|  | | | | P: | 4.82E-01 | 9.93E-01 | 3.86E-01 | 6.83E-01 | 3.52E-01 | 3.48E-01 |
|  |  |  |  | \|α\|: | 2.0999 | 0.0077 | 9.8306 | 1.2796 | 0.8671 | 0.8742 |
|  |  |  |  | cP: | 0.0002 | 0.0000 | 0.0002 | 0.0001 | 0.0008 | 0.0008 |
|  |  |  |  | cG: | 0.0013 | 0.0000 | 0.0017 | 0.0011 | 0.0058 | 0.0059 |

| **Chr 13** | | | | | | |
| --- | --- | --- | --- | --- | --- | --- |
| Element | | | | | SLIT3 | SLIT3 |
|  | bp | | | | 4,954,745 | 5,324,164 |
|  |  | Dist. | | |  | 369,419 |
|  |  |  | QTLR | | 31 | 31 |
|  |  |  |  | Marker | MD13_03A | MD13_03B |
| SLIT3 | 4,954,745 |  | 31 | MD13_03A |  |  |
| SLIT3 | 5,324,164 | 369,419 | 31 | MD13_03B | 0.010 |  |
|  | | | | P: | 1.25E-01 | 8.56E-01 |
|  |  |  |  | \|α\|: | 1.1457 | 0.3640 |
|  |  |  |  | cP: | 0.0016 | 0.0000 |
|  |  |  |  | cG: | 0.0123 | 0.0002 |

| **Chr 14** | | | | | | | | |
| --- | --- | --- | --- | --- | --- | --- | --- | --- |
| Element | | | | | HN1L | CRAMP1 | TMEM204 | ATP6V0C |
|  | bp | | | | 14,030,671 | 14,050,330 | 14,115,781 | 14,252,012 |
|  |  | Dist. | | |  | 19,659 | 65,451 | 136,231 |
|  |  |  | QTLR | | 34 | 34 | 34 | 34 |
|  |  |  |  | Marker | MD14_01A | MD14_02A | MD14_03B | MD14_04A |
| HN1L | 14,030,671 |  | 34 | MD14_01A |  |  |  |  |
| CRAMP1 | 14,050,330 | 19,659 | 34 | MD14_02A | 0.136 |  |  |  |
| TMEM204 | 14,115,781 | 65,451 | 34 | MD14_03B | 1.000 | 0.138 |  |  |
| ATP6V0C | 14,252,012 | 136,231 | 34 | MD14_04A | 0.000 | 0.000 | 0.000 |  |
|  | | | | P: | 9.70E-01 | 7.77E-01 | 9.80E-01 | 5.11E-01 |
|  |  |  |  | \|α\|: | 0.0256 | 0.4698 | 0.0170 | 0.4565 |
|  |  |  |  | cP: | 0.0000 | 0.0001 | 0.0000 | 0.0003 |
|  |  |  |  | cG: | 0.0000 | 0.0008 | 0.0000 | 0.0024 |

| **Chr 17** | | | | | | | | | | | | | | | | | |
| --- | --- | --- | --- | --- | --- | --- | --- | --- | --- | --- | --- | --- | --- | --- | --- | --- | --- |
| Element | | | | | FAM102A | FAM102A | FAM102A | DPM2 | DPM2 | ST6GALNAC6 | ENG | CDK11 | SH2D3C | SH2D3C | TOR2A | TOR2A | URM1 |
|  | bp | | | | 5,203,358 | 5,221,308 | 5,230,753 | 5,237,745 | 5,239,548 | 5,245,311 | 5,263,306 | 5,272,121 | 5,292,163 | 5,295,958 | 5,296,779 | 5,298,419 | 5,321,060 |
|  |  | Dist. | | |  | 17,950 | 9,445 | 6,992 | 1,803 | 5,763 | 17,995 | 8,815 | 20,042 | 3,795 | 821 | 1,640 | 22,641 |
|  |  |  | QTLR | | 35 | 35 | 35 | 35 | 35 | 35 | 35 | 35 | 35 | 35 | 35 | 35 | 35 |
|  |  |  |  | Marker | MD17_01A | MD17_01B | MD17_01C | MD17_02A | MD17_02B | MD17_03A | MD17_04B | MD17_05A | MD17_06A | MD17_06B | MD17_07A | MD17_07B | MD17_08A |
| FAM102A | 5,203,358 |  | 35 | MD17_01A |  |  |  |  |  |  |  |  |  |  |  |  |  |
| FAM102A | 5,221,308 | 17,950 | 35 | MD17_01B | 0.398 |  |  |  |  |  |  |  |  |  |  |  |  |
| FAM102A | 5,230,753 | 9,445 | 35 | MD17_01C | 0.398 | 1.000 |  |  |  |  |  |  |  |  |  |  |  |
| DPM2 | 5,237,745 | 6,992 | 35 | MD17_02A | 0.010 | 0.551 | 0.551 |  |  |  |  |  |  |  |  |  |  |
| DPM2 | 5,239,548 | 1,803 | 35 | MD17_02B | 0.063 | 0.646 | 0.646 | 0.218 |  |  |  |  |  |  |  |  |  |
| ST6GALNAC6 | 5,245,311 | 5,763 | 35 | MD17_03A | 0.367 | 0.146 | 0.146 | 0.264 | 0.058 |  |  |  |  |  |  |  |  |
| ENG | 5,263,306 | 17,995 | 35 | MD17_04B | 0.495 | 0.200 | 0.200 | 0.071 | 0.309 | 0.019 |  |  |  |  |  |  |  |
| CDK11 | 5,272,121 | 8,815 | 35 | MD17_05A | 0.362 | 0.841 | 0.841 | 0.445 | 0.524 | 0.138 | 0.173 |  |  |  |  |  |  |
| SH2D3C | 5,292,163 | 20,042 | 35 | MD17_06A | 0.291 | 0.098 | 0.098 | 0.148 | 0.044 | 0.654 | 0.002 | 0.190 |  |  |  |  |  |
| SH2D3C | 5,295,958 | 3,795 | 35 | MD17_06B | 0.339 | 0.857 | 0.857 | 0.526 | 0.503 | 0.163 | 0.131 | 0.810 | 0.061 |  |  |  |  |
| TOR2A | 5,296,779 | 821 | 35 | MD17_07A | 0.354 | 0.822 | 0.822 | 0.436 | 0.514 | 0.136 | 0.170 | 0.949 | 0.205 | 0.834 |  |  |  |
| TOR2A | 5,298,419 | 1,640 | 35 | MD17_07B | 0.042 | 0.544 | 0.544 | 0.211 | 0.847 | 0.054 | 0.233 | 0.511 | 0.081 | 0.630 | 0.527 |  |  |
| URM1 | 5,321,060 | 22,641 | 35 | MD17_08A | 0.368 | 0.148 | 0.148 | 0.243 | 0.058 | 0.938 | 0.017 | 0.126 | 0.677 | 0.154 | 0.142 | 0.056 |  |
|  | | | | P: | 7.49E-01 | 2.85E-01 | 2.85E-01 | 3.10E-01 | 4.44E-01 | 6.08E-01 | 8.67E-01 | 2.02E-01 | 9.56E-01 | 4.34E-01 | 4.76E-01 | 4.85E-01 | 9.66E-01 |
|  |  |  |  | \|α\|: | 0.2527 | 0.7132 | 0.7132 | 0.7139 | 0.5336 | 0.5551 | 0.1619 | 0.8434 | 0.0537 | 0.5195 | 0.4776 | 0.4861 | 0.0454 |
|  |  |  |  | cP: | 0.0001 | 0.0009 | 0.0009 | 0.0008 | 0.0004 | 0.0002 | 0.0000 | 0.0012 | 0.0000 | 0.0005 | 0.0004 | 0.0004 | 0.0000 |
|  |  |  |  | cG: | 0.0006 | 0.0067 | 0.0067 | 0.0058 | 0.0034 | 0.0016 | 0.0002 | 0.0095 | 0.0000 | 0.0035 | 0.0030 | 0.0027 | 0.0000 |

| **Chr 1** | | | | | | | | | | | | | | |
| --- | --- | --- | --- | --- | --- | --- | --- | --- | --- | --- | --- | --- | --- | --- |
| Element | | | | | TRHDE | TRHDE | TRHDE | RASSF8 | RASSF8 | RASSF8 | SSPN | SSPN | ITPR2 | ITPR2 |
|  | bp | | | | 36,735,741 | 36,839,853 | 36,879,328 | 67,554,921 | 67,564,586 | 67,569,296 | 67,629,704 | 67,629,779 | 67,659,080 | 67,810,544 |
|  |  | Dist. | | |  | 104,112 | 39,475 | 30,675,593 | 9,665 | 4,710 | 60,408 | 75 | 29,301 | 151,464 |
|  |  |  | QTLR | | 2 | 2 | 2 | 4 | 4 | 4 | 4 | 4 | 4 | 4 |
|  |  |  |  | Marker | MD01_02A | MD01_02B | MD01_02C | MD01_03A | MD01_03B | MD01_03C | MD01_04B | MD01_04C | MD01_05C | MD01_05B |
| TRHDE | 36,735,741 |  | 2 | MD01_02A |  |  |  |  |  |  |  |  |  |  |
| TRHDE | 36,839,853 | 104,112 | 2 | MD01_02B | 0.196 |  |  |  |  |  |  |  |  |  |
| TRHDE | 36,879,328 | 39,475 | 2 | MD01_02C | 0.196 | 0.994 |  |  |  |  |  |  |  |  |
| RASSF8 | 67,554,921 | 30,675,593 | 4 | MD01_03A | 0.002 | 0.002 | 0.002 |  |  |  |  |  |  |  |
| RASSF8 | 67,564,586 | 9,665 | 4 | MD01_03B | 0.001 | 0.006 | 0.006 | 0.543 |  |  |  |  |  |  |
| RASSF8 | 67,569,296 | 4,710 | 4 | MD01_03C | 0.001 | 0.006 | 0.006 | 0.542 | 0.995 |  |  |  |  |  |
| SSPN | 67,629,704 | 60,408 | 4 | MD01_04B | 0.001 | 0.002 | 0.003 | 0.993 | 0.536 | 0.537 |  |  |  |  |
| SSPN | 67,629,779 | 75 | 4 | MD01_04C | 0.001 | 0.002 | 0.003 | 0.993 | 0.536 | 0.537 | 1.000 |  |  |  |
| ITPR2 | 67,659,080 | 29,301 | 4 | MD01_05C | 0.001 | 0.002 | 0.002 | 0.954 | 0.509 | 0.511 | 0.958 | 0.958 |  |  |
| ITPR2 | 67,810,544 | 151,464 | 4 | MD01_05B | 0.001 | 0.002 | 0.002 | 0.907 | 0.492 | 0.494 | 0.909 | 0.909 | 0.949 |  |
|  | | | | P: | 8.56E-01 | 7.75E-01 | 7.91E-01 | 1.37E-01 | 2.01E-01 | 2.83E-01 | 1.37E-01 | 1.37E-01 | 6.31E-02 | 6.11E-02 |
|  |  |  |  | \|α\|: | 0.0887 | 0.1343 | 0.1244 | 0.6656 | 0.6195 | 0.5246 | 0.6666 | 0.6662 | 0.8324 | 0.8340 |
|  |  |  |  | cP: | 0.0000 | 0.0000 | 0.0000 | 0.0008 | 0.0006 | 0.0004 | 0.0008 | 0.0008 | 0.0012 | 0.0012 |
|  |  |  |  | cG: | 0.0001 | 0.0002 | 0.0002 | 0.0059 | 0.0044 | 0.0032 | 0.0059 | 0.0059 | 0.0092 | 0.0093 |

| **Chr 4** | | | | | | | | | | | |
| --- | --- | --- | --- | --- | --- | --- | --- | --- | --- | --- | --- |
| Element | | | | | ACSL1 | CCDC111 | CCDC111 | CCDC111 | CASP3 | CASP3 | CASP3 |
|  | bp | | | | 39,453,244 | 39,481,749 | 39,483,799 | 39,486,747 | 39,498,984 | 39,506,324 | 39,509,637 |
|  |  | Dist. | | |  | 28,505 | 2,050 | 2,948 | 12,237 | 7,340 | 3,313 |
|  |  |  | QTLR | | 17 | 17 | 17 | 17 | 17 | 17 | 17 |
|  |  |  |  | Marker | MD04_02A | MD04_03C | MD04_03B | MD04_03A | MD04_04A | MD04_04B | MD04_04C |
| ACSL1 | 39,453,244 |  | 17 | MD04_02A |  |  |  |  |  |  |  |
| CCDC111 | 39,481,749 | 28,505 | 17 | MD04_03C | 0.569 |  |  |  |  |  |  |
| CCDC111 | 39,483,799 | 2,050 | 17 | MD04_03B | 0.585 | 0.970 |  |  |  |  |  |
| CCDC111 | 39,486,747 | 2,948 | 17 | MD04_03A | 0.585 | 0.970 | 1.000 |  |  |  |  |
| CASP3 | 39,498,984 | 12,237 | 17 | MD04_04A | 0.985 | 0.577 | 0.593 | 0.593 |  |  |  |
| CASP3 | 39,506,324 | 7,340 | 17 | MD04_04B | 0.985 | 0.577 | 0.593 | 0.593 | 1.000 |  |  |
| CASP3 | 39,509,637 | 3,313 | 17 | MD04_04C | 0.001 | 0.000 | 0.001 | 0.000 | 0.000 | 0.002 |  |
|  | | | | P: | 2.16E-01 | 1.91E-01 | 1.61E-01 | 1.61E-01 | 1.47E-01 | 1.52E-01 | 7.76E-01 |
|  |  |  |  | \|α\|: | 0.5679 | 0.5830 | 0.6302 | 0.6287 | 0.6678 | 0.6599 | 1.1606 |
|  |  |  |  | cP: | 0.0005 | 0.0006 | 0.0007 | 0.0007 | 0.0008 | 0.0007 | 0.0000 |
|  |  |  |  | cG: | 0.0042 | 0.0045 | 0.0053 | 0.0052 | 0.0058 | 0.0057 | 0.0001 |

| **Chr 9** | | | | | | | | | | | | | | | | |
| --- | --- | --- | --- | --- | --- | --- | --- | --- | --- | --- | --- | --- | --- | --- | --- | --- |
| Element | | | | | DLG1 | DLG1 | GMNC | GMNC | intergenic | TMEM207 | TMEM207 | TMEM207 | EPHB | LAMP3 | LAMP3 | LAMP3 |
|  | bp | | | | 12,646,991 | 12,691,840 | 13,960,302 | 13,967,979 | 13,991,344 | 14,069,448 | 14,072,014 | 14,073,188 | 16,120,414 | 16,532,498 | 16,537,751 | 16,540,194 |
|  |  | Dist. | | |  | 44,849 | 1,268,462 | 7,677 | 23,365 | 78,104 | 2,566 | 1,174 | 2,047,226 | 412,084 | 5,253 | 2,443 |
|  |  |  | QTLR | | 28 | 28 | 28 | 28 | 28 | 28 | 28 | 28 | 28 | 28 | 28 | 28 |
|  |  |  |  | Marker | MD09_01B | MD09_01A | MD09_02A | MD09_02B | MD09_03A | MD09_06A | MD09_06B | MD09_06C | MD09_07C | MD09_08B | MD09_08C | MD09_08D |
| DLG1 | 12,646,991 |  | 28 | MD09_01B |  |  |  |  |  |  |  |  |  |  |  |  |
| DLG1 | 12,691,840 | 44,849 | 28 | MD09_01A | 0.787 |  |  |  |  |  |  |  |  |  |  |  |
| GMNC | 13,960,302 | 1,268,462 | 28 | MD09_02A | 0.002 | 0.002 |  |  |  |  |  |  |  |  |  |  |
| GMNC | 13,967,979 | 7,677 | 28 | MD09_02B | 0.002 | 0.002 | 1.000 |  |  |  |  |  |  |  |  |  |
| intergenic | 13,991,344 | 23,365 | 28 | MD09_03A | 0.000 | 0.000 | 0.000 | 0.000 |  |  |  |  |  |  |  |  |
| TMEM207 | 14,069,448 | 78,104 | 28 | MD09_06A | 0.005 | 0.057 | 0.003 | 0.003 | 0.000 |  |  |  |  |  |  |  |
| TMEM207 | 14,072,014 | 2,566 | 28 | MD09_06B | 0.002 | 0.062 | 0.006 | 0.006 | 0.000 | 0.926 |  |  |  |  |  |  |
| TMEM207 | 14,073,188 | 1,174 | 28 | MD09_06C | 0.002 | 0.061 | 0.000 | 0.000 | 0.000 | 0.925 | 1.000 |  |  |  |  |  |
| EPHB | 16,120,414 | 2,047,226 | 28 | MD09_07C | 0.000 | 0.000 | 0.018 | 0.018 | 0.000 | 0.001 | 0.009 | 0.008 |  |  |  |  |
| LAMP3 | 16,532,498 | 412,084 | 28 | MD09_08B | 0.000 | 0.000 | 0.001 | 0.001 | 0.000 | 0.021 | 0.034 | 0.034 | 0.052 |  |  |  |
| LAMP3 | 16,537,751 | 5,253 | 28 | MD09_08C | 0.000 | 0.001 | 0.001 | 0.001 | 0.000 | 0.028 | 0.044 | 0.044 | 0.060 | 0.898 |  |  |
| LAMP3 | 16,540,194 | 2,443 | 28 | MD09_08D | 0.000 | 0.001 | 0.001 | 0.001 | 0.000 | 0.028 | 0.044 | 0.044 | 0.060 | 0.898 | 1.000 |  |
|  | | | | P: | 1.26E-01 | 9.85E-02 | 5.03E-02 | 5.35E-02 | 5.11E-01 | 2.42E-01 | 1.72E-01 | 2.35E-01 | 5.63E-01 | 4.86E-01 | 2.35E-01 | 2.35E-01 |
|  |  |  |  | \|α\|: | 0.8177 | 0.8500 | 13.7464 | 13.5678 | 6.4682 | 1.0004 | 1.1008 | 0.9569 | 1.0940 | 0.4664 | 0.8276 | 0.8276 |
|  |  |  |  | cP: | 0.0009 | 0.0010 | 0.0009 | 0.0008 | 0.0001 | 0.0006 | 0.0007 | 0.0006 | 0.0001 | 0.0002 | 0.0005 | 0.0005 |
|  |  |  |  | cG: | 0.0066 | 0.0080 | 0.0066 | 0.0064 | 0.0007 | 0.0044 | 0.0057 | 0.0043 | 0.0006 | 0.0012 | 0.0036 | 0.0036 |

| **Chr 13** | | | | | | |
| --- | --- | --- | --- | --- | --- | --- |
| Element | | | | | SLIT3 | SLIT3 |
|  | bp | | | | 5,324,164 | 5,421,966 |
|  |  | Dist. | | |  | 97,802 |
|  |  |  | QTLR | | 31 | 31 |
|  |  |  |  | Marker | MD13_03B | MD13_03C |
| SLIT3 | 5,324,164 |  | 31 | MD13_03B |  |  |
| SLIT3 | 5,421,966 | 97,802 | 31 | MD13_03C | 0.940 |  |
|  | | | | P: | 9.13E-01 | 9.33E-01 |
|  |  |  |  | \|α\|: | 0.0734 | 0.0584 |
|  |  |  |  | cP: | 0.0000 | 0.0000 |
|  |  |  |  | cG: | 0.0000 | 0.0000 |

| **Chr 14** | | | | | | | |
| --- | --- | --- | --- | --- | --- | --- | --- |
| Element | | | | | HN1L | CRAMP1 | TMEM204 |
|  | bp | | | | 14,030,671 | 14,050,330 | 14,142,117 |
|  |  | Dist. | | |  | 19,659 | 91,787 |
|  |  |  | QTLR | | 34 | 34 | 34 |
|  |  |  |  | Marker | MD14_01A | MD14_02A | MD14_03A |
| HN1L | 14,030,671 |  | 34 | MD14_01A |  |  |  |
| CRAMP1 | 14,050,330 | 19,659 | 34 | MD14_02A | 0.000 |  |  |
| TMEM204 | 14,142,117 | 91,787 | 34 | MD14_03A | 0.000 | 0.064 |  |
|  | | | | P: | 5.66E-01 | 6.32E-02 | 8.43E-01 |
|  |  |  |  | \|α\|: | 5.6475 | 2.6360 | 0.0991 |
|  |  |  |  | cP: | 0.0001 | 0.0010 | 0.0000 |
|  |  |  |  | cG: | 0.0006 | 0.0075 | 0.0001 |

| **Chr 17** | | | | | | | | | | | | | |
| --- | --- | --- | --- | --- | --- | --- | --- | --- | --- | --- | --- | --- | --- |
| Element | | | | | FAM102A | FAM102A | DPM2 | DPM2 | ST6GALNAC6 | CDK10 | SH2D3C | TOR2A | URM1 |
|  | bp | | | | 5,203,358 | 5,230,753 | 5,237,745 | 5,239,548 | 5,245,311 | 5,269,149 | 5,292,163 | 5,296,779 | 5,321,060 |
|  |  | Dist. | | |  | 27,395 | 6,992 | 1,803 | 5,763 | 23,838 | 23,014 | 4,616 | 24,281 |
|  |  |  | QTLR | | 35 | 35 | 35 | 35 | 35 | 35 | 35 | 35 | 35 |
|  |  |  |  | Marker | MD17_01A | MD17_01C | MD17_02A | MD17_02B | MD17_03A | MD17_05B | MD17_06A | MD17_07A | MD17_08A |
| FAM102A | 5,203,358 |  | 35 | MD17_01A |  |  |  |  |  |  |  |  |  |
| FAM102A | 5,230,753 | 27,395 | 35 | MD17_01C | 0.001 |  |  |  |  |  |  |  |  |
| DPM2 | 5,237,745 | 6,992 | 35 | MD17_02A | 0.991 | 0.001 |  |  |  |  |  |  |  |
| DPM2 | 5,239,548 | 1,803 | 35 | MD17_02B | 0.002 | 0.969 | 0.003 |  |  |  |  |  |  |
| ST6GALNAC6 | 5,245,311 | 5,763 | 35 | MD17_03A | 0.928 | 0.034 | 0.937 | 0.031 |  |  |  |  |  |
| CDK10 | 5,269,149 | 23,838 | 35 | MD17_05B | 0.796 | 0.001 | 0.801 | 0.001 | 0.763 |  |  |  |  |
| SH2D3C | 5,292,163 | 23,014 | 35 | MD17_06A | 0.758 | 0.001 | 0.765 | 0.001 | 0.729 | 0.951 |  |  |  |
| TOR2A | 5,296,779 | 4,616 | 35 | MD17_07A | 0.707 | 0.033 | 0.716 | 0.033 | 0.776 | 0.887 | 0.930 |  |  |
| URM1 | 5,321,060 | 24,281 | 35 | MD17_08A | 0.414 | 0.001 | 0.417 | 0.001 | 0.401 | 0.525 | 0.575 | 0.534 |  |
|  | | | | P: | 9.07E-01 | 6.77E-02 | 8.58E-01 | 6.73E-02 | 8.11E-01 | 7.88E-01 | 7.42E-01 | 3.91E-01 | 9.89E-01 |
|  |  |  |  | \|α\|: | 0.0591 | 3.3650 | 0.0894 | 3.4892 | 0.1179 | 0.1452 | 0.1734 | 0.4421 | 0.0086 |
|  |  |  |  | cP: | 0.0000 | 0.0009 | 0.0000 | 0.0009 | 0.0000 | 0.0000 | 0.0000 | 0.0002 | 0.0000 |
|  |  |  |  | cG: | 0.0000 | 0.0068 | 0.0001 | 0.0067 | 0.0001 | 0.0002 | 0.0003 | 0.0018 | 0.0000 |

| **Chr 1** | | | | | | | | |
| --- | --- | --- | --- | --- | --- | --- | --- | --- |
| Element | | | | | TRHDE | RASSF8 | RASSF8 | RASSF8 |
|  | bp | | | | 36,839,853 | 67,554,921 | 67,564,586 | 67,569,296 |
|  |  | Dist. | | |  | 30,715,068 | 9,665 | 4,710 |
|  |  |  | QTLR | | 2 | 4 | 4 | 4 |
|  |  |  |  | Marker | MD01_02B | MD01_03A | MD01_03B | MD01_03C |
| TRHDE | 36,839,853 |  | 2 | MD01_02B |  |  |  |  |
| RASSF8 | 67,554,921 | 30,715,068 | 4 | MD01_03A | 0.002 |  |  |  |
| RASSF8 | 67,564,586 | 9,665 | 4 | MD01_03B | 0.002 | 1.000 |  |  |
| RASSF8 | 67,569,296 | 4,710 | 4 | MD01_03C | 0.000 | 0.231 | 0.231 |  |
|  | | | | P: | 6.22E-01 | 3.24E-01 | 3.21E-01 | 1.21E-01 |
|  |  |  |  | \|α\|: | 0.3244 | 1.3976 | 1.4061 | 4.1609 |
|  |  |  |  | cP: | 0.0000 | 0.0002 | 0.0002 | 0.0003 |
|  |  |  |  | cG: | 0.0004 | 0.0012 | 0.0013 | 0.0026 |

| **Chr 4** | | | | | | | | |
| --- | --- | --- | --- | --- | --- | --- | --- | --- |
| Element | | | | | CCDC111 | CCDC111 | CCDC111 | CASP3 |
|  | bp | | | | 39,481,749 | 39,483,799 | 39,486,747 | 39,509,637 |
|  |  | Dist. | | |  | 2,050 | 2,948 | 22,890 |
|  |  |  | QTLR | | 17 | 17 | 17 | 17 |
|  |  |  |  | Marker | MD04_03C | MD04_03B | MD04_03A | MD04_04C |
| CCDC111 | 39,481,749 |  | 17 | MD04_03C |  |  |  |  |
| CCDC111 | 39,483,799 | 2,050 | 17 | MD04_03B | 0.977 |  |  |  |
| CCDC111 | 39,486,747 | 2,948 | 17 | MD04_03A | 0.098 | 0.097 |  |  |
| CASP3 | 39,509,637 | 22,890 | 17 | MD04_04C | 0.000 | 0.022 | 0.000 |  |
|  | | | | P: | 1.01E-01 | 1.32E-01 | 5.82E-02 | 6.68E-01 |
|  |  |  |  | \|α\|: | 2.7261 | 2.4538 | 1.1655 | 4.2523 |
|  |  |  |  | cP: | 0.0004 | 0.0003 | 0.0006 | 0.0000 |
|  |  |  |  | cG: | 0.0031 | 0.0026 | 0.0046 | 0.0002 |

| **Chr 9** | | | | | | | | | | | | | | |
| --- | --- | --- | --- | --- | --- | --- | --- | --- | --- | --- | --- | --- | --- | --- |
| Element | | | | | DLG1 | GMNC | gga-mir-1762 | gga-mir-1762 | gga-mir-1762 | TMEM207 | TMEM207 | LAMP3 | LAMP3 | LAMP3 |
|  | bp | | | | 12,691,840 | 13,967,979 | 14,044,716 | 14,044,717 | 14,044,719 | 14,072,014 | 14,073,188 | 16,532,498 | 16,537,751 | 16,540,194 |
|  |  | Dist. | | |  | 1,276,139 | 76,737 | 1 | 2 | 27,295 | 1,174 | 2,459,310 | 5,253 | 2,443 |
|  |  |  | QTLR | | 28 | 28 | 28 | 28 | 28 | 28 | 28 | 28 | 28 | 28 |
|  |  |  |  | Marker | MD09_01A | MD09_02B | MD09_05Da | MD09_05Db | MD09_05C | MD09_06B | MD09_06C | MD09_08B | MD09_08C | MD09_08D |
| DLG1 | 12,691,840 |  | 28 | MD09_01A |  |  |  |  |  |  |  |  |  |  |
| GMNC | 13,967,979 | 1,276,139 | 28 | MD09_02B | 0.002 |  |  |  |  |  |  |  |  |  |
| gga-mir-1762 | 14,044,716 | 76,737 | 28 | MD09_05Da | 0.000 | 0.226 |  |  |  |  |  |  |  |  |
| gga-mir-1762 | 14,044,717 | 1 | 28 | MD09_05Db | 0.000 | 0.226 | 1.000 |  |  |  |  |  |  |  |
| gga-mir-1762 | 14,044,719 | 2 | 28 | MD09_05C | 0.000 | 0.255 | 0.980 | 0.980 |  |  |  |  |  |  |
| TMEM207 | 14,072,014 | 27,295 | 28 | MD09_06B | 0.000 | 0.260 | 0.980 | 0.980 | 1.000 |  |  |  |  |  |
| TMEM207 | 14,073,188 | 1,174 | 28 | MD09_06C | 0.001 | 0.257 | 0.979 | 0.979 | 1.000 | 1.000 |  |  |  |  |
| LAMP3 | 16,532,498 | 2,459,310 | 28 | MD09_08B | 0.002 | 0.001 | 0.001 | 0.001 | 0.001 | 0.001 | 0.002 |  |  |  |
| LAMP3 | 16,537,751 | 5,253 | 28 | MD09_08C | 0.002 | 0.001 | 0.001 | 0.001 | 0.001 | 0.001 | 0.002 | 1.000 |  |  |
| LAMP3 | 16,540,194 | 2,443 | 28 | MD09_08D | 0.000 | 0.000 | 0.000 | 0.000 | 0.000 | 0.000 | 0.000 | 0.000 | 0.000 |  |
|  | | | | P: | 4.91E-01 | 1.62E-01 | 2.79E-02 | 2.79E-02 | 3.31E-02 | 3.19E-02 | 5.41E-02 | 2.33E-01 | 2.31E-01 | 7.21E-01 |
|  |  |  |  | \|α\|: | 1.2733 | 1.8752 | 1.8346 | 1.8346 | 1.7810 | 1.7977 | 1.6375 | 0.4931 | 0.4955 | 3.5500 |
|  |  |  |  | cP: | 0.0001 | 0.0003 | 0.0008 | 0.0008 | 0.0008 | 0.0008 | 0.0007 | 0.0003 | 0.0003 | 0.0000 |
|  |  |  |  | cG: | 0.0007 | 0.0022 | 0.0065 | 0.0065 | 0.0062 | 0.0063 | 0.0052 | 0.0021 | 0.0021 | 0.0001 |

| **Chr 14** | | | | | | | |
| --- | --- | --- | --- | --- | --- | --- | --- |
| Element | | | | | CRAMP1 | TMEM204 | ATP6V0C |
|  | bp | | | | 14,050,330 | 14,142,117 | 14,252,012 |
|  |  | Dist. | | |  | 91,787 | 109,895 |
|  |  |  | QTLR | | 34 | 34 | 34 |
|  |  |  |  | Marker | MD14_02A | MD14_03A | MD14_04A |
| CRAMP1 | 14,050,330 |  | 34 | MD14_02A |  |  |  |
| TMEM204 | 14,142,117 | 91,787 | 34 | MD14_03A | 0.000 |  |  |
| ATP6V0C | 14,252,012 | 109,895 | 34 | MD14_04A | 0.000 | 0.000 |  |
|  | | | | P: | 2.66E-03 | 1.02E-02 | 6.74E-01 |
|  |  |  |  | \|α\|: | 1.2361 | 8.1822 | 4.1692 |
|  |  |  |  | cP: | 0.0017 | 0.0009 | 0.0000 |
|  |  |  |  | cG: | 0.0128 | 0.0066 | 0.0002 |

| **Chr 17** | | | | | | |
| --- | --- | --- | --- | --- | --- | --- |
| Element | | | | | ST6GALNAC6 | ST6GALNAC6 |
|  | bp | | | | 5,245,311 | 5,247,038 |
|  |  | Dist. | | |  | 1,727 |
|  |  |  | QTLR | | 35 | 35 |
|  |  |  |  | Marker | MD17_03A | MD17_03C |
| ST6GALNAC6 | 5,245,311 |  | 35 | MD17_03A |  |  |
| ST6GALNAC6 | 5,247,038 | 1,727 | 35 | MD17_03C | 1.000 |  |
|  | | | | P: | 6.70E-01 | 6.82E-01 |
|  |  |  |  | \|α\|: | 4.2213 | 4.0645 |
|  |  |  |  | cP: | 0.0000 | 0.0000 |
|  |  |  |  | cG: | 0.0002 | 0.0002 |

| **Chr 1** | | | | | | | | | | |
| --- | --- | --- | --- | --- | --- | --- | --- | --- | --- | --- |
| Element | | | | | TRHDE | TRHDE | TRHDE | RASSF8 | RASSF8 | ITPR2 |
|  | bp | | | | 36,839,853 | 36,879,328 | 36,951,385 | 67,564,586 | 67,569,296 | 67,882,133 |
|  |  | Dist. | | |  | 39,475 | 72,057 | 30,613,201 | 4,710 | 312,837 |
|  |  |  | QTLR | | 2 | 2 | 2 | 4 | 4 | 4 |
|  |  |  |  | Marker | MD01_02B | MD01_02C | MD01_02D | MD01_03B | MD01_03C | MD01_05A |
| TRHDE | 36,839,853 |  | 2 | MD01_02B |  |  |  |  |  |  |
| TRHDE | 36,879,328 | 39475 | 2 | MD01_02C | 0.007 |  |  |  |  |  |
| TRHDE | 36,951,385 | 72057 | 2 | MD01_02D | 1.000 | 0.007 |  |  |  |  |
| RASSF8 | 67,564,586 | 30,613,201 | 4 | MD01_03B | 0.000 | 0.001 | 0.000 |  |  |  |
| RASSF8 | 67,569,296 | 4,710 | 4 | MD01_03C | 0.003 | 0.003 | 0.003 | 0.004 |  |  |
| ITPR2 | 67,882,133 | 312,837 | 4 | MD01_05A | 0.003 | 0.008 | 0.003 | 0.004 | 0.883 |  |
|  | | | | P: | 9.06E-01 | 1.09E-01 | 9.06E-01 | 2.26E-01 | 1.45E-01 | 1.99E-01 |
|  |  |  |  | \|α\|: | 0.3600 | 0.9162 | 0.3600 | 13.6247 | 1.2945 | 1.1418 |
|  |  |  |  | cP: | 0.0000 | 0.0015 | 0.0000 | 0.0005 | 0.0010 | 0.0007 |
|  |  |  |  | cG: | 0.0000 | 0.0119 | 0.0000 | 0.0039 | 0.0075 | 0.0055 |

| **Chr 4** | | | | | | | |
| --- | --- | --- | --- | --- | --- | --- | --- |
| Element | | | | | ACSL1 | CCDC111 | CCDC111 |
|  | bp | | | | 39,453,244 | 39,483,799 | 39,486,747 |
|  |  | Dist. | | |  | 30,555 | 2,948 |
|  |  |  | QTLR | | 17 | 17 | 17 |
|  |  |  |  | Marker | MD04_02A | MD04_03B | MD04_03A |
| ACSL1 | 39,453,244 |  | 17 | MD04_02A |  |  |  |
| CCDC111 | 39,483,799 | 30,555 | 17 | MD04_03B | 1.000 |  |  |
| CCDC111 | 39,486,747 | 2,948 | 17 | MD04_03A | 1.000 | 1.000 |  |
|  | | | | P: | 7.17E-01 | 7.33E-01 | 7.33E-01 |
|  |  |  |  | \|α\|: | 0.1963 | 0.1844 | 0.1844 |
|  |  |  |  | cP: | 0.0001 | 0.0001 | 0.0001 |
|  |  |  |  | cG: | 0.0005 | 0.0005 | 0.0005 |

| **Chr 9** | | | | | | | | | | | | | | | | | | | |
| --- | --- | --- | --- | --- | --- | --- | --- | --- | --- | --- | --- | --- | --- | --- | --- | --- | --- | --- | --- |
| Element | | | | | DLG1 | DLG1 | GMNC | GMNC | intergenic | IL1RAP | gga-mir-1762 | gga-mir-1762 | gga-mir-1762 | TMEM207 | TMEM207 | TMEM207 | EPHB | LAMP3 | LAMP3 |
|  | bp | | | | 12,646,991 | 12,691,840 | 13,960,302 | 13,967,979 | 13,991,344 | 14,024,326 | 14,044,716 | 14,044,717 | 14,044,719 | 14,069,448 | 14,072,014 | 14,073,188 | 16,120,414 | 16,532,498 | 16,537,751 |
|  |  | Dist. | | |  | 44,849 | 1,268,462 | 7,677 | 23,365 | 32,982 | 20,390 | 1 | 2 | 24,729 | 2,566 | 1,174 | 2,047,226 | 412,084 | 5,253 |
|  |  |  | QTLR | | 28 | 28 | 28 | 28 | 28 | 28 | 28 | 28 | 28 | 28 | 28 | 28 | 28 | 28 | 28 |
|  |  |  |  | Marker | MD09_01B | MD09_01A | MD09_02A | MD09_02B | MD09_03A | MD09_04A | MD09_05Da | MD09_05Db | MD09_05C | MD09_06A | MD09_06B | MD09_06C | MD09_07C | MD09_08B | MD09_08C |
| DLG1 | 12,646,991 |  | 28 | MD09_01B |  |  |  |  |  |  |  |  |  |  |  |  |  |  |  |
| DLG1 | 12,691,840 | 44,849 | 28 | MD09_01A | 0.030 |  |  |  |  |  |  |  |  |  |  |  |  |  |  |
| GMNC | 13,960,302 | 1,268,462 | 28 | MD09_02A | 0.060 | 0.191 |  |  |  |  |  |  |  |  |  |  |  |  |  |
| GMNC | 13,967,979 | 7,677 | 28 | MD09_02B | 0.056 | 0.187 | 0.981 |  |  |  |  |  |  |  |  |  |  |  |  |
| intergenic | 13,991,344 | 23,365 | 28 | MD09_03A | 0.052 | 0.171 | 0.898 | 0.914 |  |  |  |  |  |  |  |  |  |  |  |
| IL1RAP | 14,024,326 | 32,982 | 28 | MD09_04A | 0.052 | 0.171 | 0.898 | 0.914 | 1.000 |  |  |  |  |  |  |  |  |  |  |
| gga-mir-1762 | 14,044,716 | 20,390 | 28 | MD09_05Da | 0.006 | 0.001 | 0.005 | 0.000 | 0.058 | 0.058 |  |  |  |  |  |  |  |  |  |
| gga-mir-1762 | 14,044,717 | 1 | 28 | MD09_05Db | 0.010 | 0.436 | 0.196 | 0.215 | 0.339 | 0.339 | 0.155 |  |  |  |  |  |  |  |  |
| gga-mir-1762 | 14,044,719 | 2 | 28 | MD09_05C | 0.006 | 0.001 | 0.005 | 0.000 | 0.059 | 0.059 | 1.000 | 0.157 |  |  |  |  |  |  |  |
| TMEM207 | 14,069,448 | 24,729 | 28 | MD09_06A | 0.018 | 0.534 | 0.283 | 0.284 | 0.276 | 0.276 | 0.000 | 0.809 | 0.000 |  |  |  |  |  |  |
| TMEM207 | 14,072,014 | 2,566 | 28 | MD09_06B | 0.023 | 0.007 | 0.565 | 0.551 | 0.508 | 0.508 | 0.003 | 0.017 | 0.003 | 0.013 |  |  |  |  |  |
| TMEM207 | 14,073,188 | 1,174 | 28 | MD09_06C | 0.017 | 0.007 | 0.491 | 0.484 | 0.568 | 0.568 | 0.071 | 0.002 | 0.072 | 0.017 | 0.856 |  |  |  |  |
| EPHB | 16,120,414 | 2,047,226 | 28 | MD09_07C | 0.016 | 0.010 | 0.071 | 0.070 | 0.072 | 0.072 | 0.000 | 0.008 | 0.000 | 0.008 | 0.041 | 0.041 |  |  |  |
| LAMP3 | 16,532,498 | 412,084 | 28 | MD09_08B | 0.016 | 0.005 | 0.027 | 0.028 | 0.025 | 0.025 | 0.001 | 0.001 | 0.001 | 0.002 | 0.034 | 0.032 | 0.007 |  |  |
| LAMP3 | 16,537,751 | 5,253 | 28 | MD09_08C | 0.016 | 0.005 | 0.027 | 0.028 | 0.025 | 0.025 | 0.001 | 0.001 | 0.001 | 0.002 | 0.034 | 0.032 | 0.007 | 1.000 |  |
|  | | | | P: | 4.75E-02 | 1.23E-01 | 3.90E-01 | 6.59E-01 | 9.42E-01 | 9.42E-01 | 1.95E-01 | 1.57E-01 | 1.98E-01 | 1.76E-02 | 1.47E-01 | 5.47E-02 | 4.56E-01 | 5.82E-03 | 5.82E-03 |
|  |  |  |  | \|α\|: | 1.0844 | 1.8246 | 0.5879 | 0.2924 | 0.0480 | 0.0480 | 2.2452 | 1.1710 | 2.2551 | 2.1457 | 1.2142 | 1.5122 | 0.4161 | 1.5159 | 1.5159 |
|  |  |  |  | cP: | 0.0020 | 0.0014 | 0.0004 | 0.0001 | 0.0000 | 0.0000 | 0.0007 | 0.0010 | 0.0007 | 0.0028 | 0.0013 | 0.0022 | 0.0003 | 0.0042 | 0.0042 |
|  |  |  |  | cG: | 0.0152 | 0.0106 | 0.0035 | 0.0009 | 0.0000 | 0.0000 | 0.0052 | 0.0076 | 0.0053 | 0.0215 | 0.0103 | 0.0172 | 0.0024 | 0.0323 | 0.0323 |

| **Chr 13** | | | | | | |
| --- | --- | --- | --- | --- | --- | --- |
| Element | | | | | SLIT3 | SLIT3 |
|  | bp | | | | 5,324,164 | 5,421,966 |
|  |  | Dist. | | |  | 97,802 |
|  |  |  | QTLR | | 31 | 31 |
|  |  |  |  | Marker | MD13_03B | MD13_03C |
| SLIT3 | 5,324,164 |  | 31 | MD13_03B |  |  |
| SLIT3 | 5,421,966 | 97,802 | 31 | MD13_03C | 0.013 |  |
|  | | | | P: | 4.92E-01 | 2.07E-01 |
|  |  |  |  | \|α\|: | 1.3461 | 0.6780 |
|  |  |  |  | cP: | 0.0002 | 0.0009 |
|  |  |  |  | cG: | 0.0014 | 0.0067 |

| **Chr 14** | | | | | | | | |
| --- | --- | --- | --- | --- | --- | --- | --- | --- |
| Element | | | | | HN1L | CRAMP1 | TMEM204 | ATP6V0C |
|  | bp | | | | 14,030,671 | 14,050,330 | 14,142,117 | 14,252,012 |
|  |  | Dist. | | |  | 19,659 | 91,787 | 109,895 |
|  |  |  | QTLR | | 34 | 34 | 34 | 34 |
|  |  |  |  | Marker | MD14_01A | MD14_02A | MD14_03A | MD14_04A |
| HN1L | 14,030,671 |  | 34 | MD14_01A |  |  |  |  |
| CRAMP1 | 14,050,330 | 19659 | 34 | MD14_02A | 0.000 |  |  |  |
| TMEM204 | 14,142,117 | 91,787 | 34 | MD14_03A | 0.262 | 0.001 |  |  |
| ATP6V0C | 14,252,012 | 109,895 | 34 | MD14_04A | 0.002 | 0.001 | 0.000 |  |
|  | | | | P: | 1.45E-01 | 8.95E-01 | 1.75E-02 | 2.14E-01 |
|  |  |  |  | \|α\|: | 1.1663 | 0.4605 | 1.5876 | 0.8450 |
|  |  |  |  | cP: | 0.0010 | 0.0000 | 0.0030 | 0.0009 |
|  |  |  |  | cG: | 0.0076 | 0.0001 | 0.0232 | 0.0068 |

| **Chr 17** | | | | | | | | | | | | | | | | | | |
| --- | --- | --- | --- | --- | --- | --- | --- | --- | --- | --- | --- | --- | --- | --- | --- | --- | --- | --- |
| Element | | | | | FAM102A | FAM102A | FAM102A | DPM2 | DPM2 | ST6GALNAC6 | ST6GALNAC6 | ST6GALNAC6 | ENG | CDK10 | CDK11 | SH2D3C | SH2D3C | TOR2A |
|  | bp | | | | 5,203,358 | 5,221,308 | 5,230,753 | 5,237,745 | 5,239,548 | 5,245,311 | 5,246,277 | 5,247,038 | 5,263,306 | 5,269,149 | 5,272,121 | 5,292,163 | 5,295,958 | 5,298,419 |
|  |  | Dist. | | |  | 17,950 | 9,445 | 6,992 | 1,803 | 5,763 | 966 | 761 | 16,268 | 5,843 | 2,972 | 20,042 | 3,795 | 2,461 |
|  |  |  | QTLR | | 35 | 35 | 35 | 35 | 35 | 35 | 35 | 35 | 35 | 35 | 35 | 35 | 35 | 35 |
|  |  |  |  | Marker | MD17_01A | MD17_01B | MD17_01C | MD17_02A | MD17_02B | MD17_03A | MD17_03B | MD17_03C | MD17_04B | MD17_05B | MD17_05A | MD17_06A | MD17_06B | MD17_07B |
| FAM102A | 5,203,358 |  | 35 | MD17_01A |  |  |  |  |  |  |  |  |  |  |  |  |  |  |
| FAM102A | 5,221,308 | 17,950 | 35 | MD17_01B | 0.071 |  |  |  |  |  |  |  |  |  |  |  |  |  |
| FAM102A | 5,230,753 | 9,445 | 35 | MD17_01C | 0.071 | 0.949 |  |  |  |  |  |  |  |  |  |  |  |  |
| DPM2 | 5,237,745 | 6,992 | 35 | MD17_02A | 0.055 | 0.712 | 0.751 |  |  |  |  |  |  |  |  |  |  |  |
| DPM2 | 5,239,548 | 1,803 | 35 | MD17_02B | 0.018 | 0.177 | 0.141 | 0.000 |  |  |  |  |  |  |  |  |  |  |
| ST6GALNAC6 | 5,245,311 | 5,763 | 35 | MD17_03A | 0.000 | 0.036 | 0.002 | 0.003 | 0.076 |  |  |  |  |  |  |  |  |  |
| ST6GALNAC6 | 5,246,277 | 966 | 35 | MD17_03B | 0.336 | 0.013 | 0.013 | 0.010 | 0.000 | 0.001 |  |  |  |  |  |  |  |  |
| ST6GALNAC6 | 5,247,038 | 761 | 35 | MD17_03C | 0.334 | 0.006 | 0.013 | 0.010 | 0.000 | 0.001 | 0.995 |  |  |  |  |  |  |  |
| ENG | 5,263,306 | 16,268 | 35 | MD17_04B | 0.088 | 0.754 | 0.796 | 0.608 | 0.101 | 0.002 | 0.000 | 0.000 |  |  |  |  |  |  |
| CDK10 | 5,269,149 | 5,843 | 35 | MD17_05B | 0.017 | 0.213 | 0.224 | 0.000 | 0.563 | 0.000 | 0.003 | 0.003 | 0.168 |  |  |  |  |  |
| CDK11 | 5,272,121 | 2,972 | 35 | MD17_05A | 0.285 | 0.002 | 0.005 | 0.010 | 0.001 | 0.001 | 0.907 | 0.915 | 0.005 | 0.000 |  |  |  |  |
| SH2D3C | 5,292,163 | 20,042 | 35 | MD17_06A | 0.003 | 0.016 | 0.001 | 0.002 | 0.346 | 0.223 | 0.000 | 0.002 | 0.001 | 0.000 | 0.002 |  |  |  |
| SH2D3C | 5,295,958 | 3,795 | 35 | MD17_06B | 0.003 | 0.016 | 0.001 | 0.002 | 0.346 | 0.223 | 0.000 | 0.002 | 0.001 | 0.000 | 0.002 | 1.000 |  |  |
| TOR2A | 5,298,419 | 2,461 | 35 | MD17_07B | 0.013 | 0.052 | 0.048 | 0.058 | 0.005 | 0.003 | 0.303 | 0.308 | 0.070 | 0.000 | 0.307 | 0.011 | 0.011 |  |
|  | | | | P: | 5.12E-01 | 6.18E-01 | 9.51E-01 | 4.09E-01 | 9.43E-01 | 1.34E-01 | 8.08E-01 | 8.00E-01 | 4.19E-01 | 2.61E-01 | 6.42E-01 | 1.18E-01 | 1.21E-01 | 1.59E-01 |
|  |  |  |  | \|α\|: | 0.4113 | 0.8532 | 0.1093 | 1.7546 | 0.1874 | 8.5070 | 0.1359 | 0.1414 | 1.3163 | 3.6131 | 0.2649 | 6.4249 | 6.3542 | 0.9599 |
|  |  |  |  | cP: | 0.0002 | 0.0001 | 0.0000 | 0.0004 | 0.0000 | 0.0010 | 0.0000 | 0.0000 | 0.0003 | 0.0005 | 0.0001 | 0.0009 | 0.0009 | 0.0011 |
|  |  |  |  | cG: | 0.0017 | 0.0009 | 0.0000 | 0.0029 | 0.0000 | 0.0076 | 0.0003 | 0.0003 | 0.0025 | 0.0036 | 0.0010 | 0.0069 | 0.0068 | 0.0088 |

**Figure S1/supplementary figure 1.** Individually genotyped QTLRs.

The chromosome is presented on the top of each page; x-axis is location in Mb, y-axis is a marker -Log_10_P obtained by JMP Genomics SNP – Trait Association Trend test; each dot is a marker test in a line; QTLRs are presented as bars on the top of the charts.

**Testing QTLRs by individual genotyping**

# QTLR 2 on GGA1

QTLR 2 was found by analysis of the pools of Line WL2 (Table 1), and was tested in the 8 lines by individual genotyping of a total of 4 markers located in the *TRHDE* gene (Table S10).

Indeed, only Line WL2 with 3 informative markers was significant or approached significance (0.10 ≥ *P* > 0.05) by the individual genotyping, along with the Across Line tests. The 2 significant markers had identical *P*-values, and were in complete LD with each other (Table S12). Thus, the region around 36.7 - 36.8 Mb on GGA 1 in the *TRHDE* gene seems to harbour the location of the causative mutation in this QTLR. *TRHDE* (Thyrotropin Releasing Hormone Degrading Enzyme) is a member of the peptidase M1 family, an extracellular peptidase that specifically cleaves and inactivates the neuropeptide thyrotropin-releasing hormone [43]. MD infects the nervous system and causes neurological damage, so it is possible to see a potential functional role for this neuropeptide-degrading enzyme.

The 2 highly significant markers in Line WL2 had exactly the same *P*-value, 7.5E-04 (Table S10). MD01_02A, is upstream of the *TRHDE* gene (Table S8), while MD01_02B is a silent mutation. Thus, both are unlikely to be the causative mutation. The actual causative element(s) could be part of *TRHDE* gene, for example a regulatory mutation in the promoter, or could be independent element(s) in high linkage with *TRHDE*. More study is needed to confirm.

# QTLR 4 on GGA1

QTLR 4 was found by the pools of Line WPR2 (Table 1), and was tested by individual genotyping of 8 markers located in 3 protein coding genes (Table S10). As mentioned above, no QTLR marker was significant by individual genotyping in Line WPR2. Nor was any other test significant in this QTLR (Table 4, Table S10). This QTLR was highly informative, namely the tested markers were well distributed over the entire QTLR, all lines distributed for some of the markers (to a total of 53 association test), and markers in all 3 genes were informative.

Thus, QTLR 4 could be a case of false positive error.

# QTLR 31 on GGA13

QTLR 31 was found by the pools in Line WL2 (Table 1). Only 3 markers were tested within and around the QTLR by individual genotyping, and none of the 18 association tests in this QTLR were significant

(Table 4 and Table S10). Thus, just like QTLR 4, QTLR 13 could be a case of a false positive.

However, more markers require to be tested for a definite conclusion.

# QTLR 34 on GGA14

QTLR 34 was found by the pools in Line WL1 (Table 1), and also found to be significant by individual genotyping (Table S10), confirming QTLR 31 identified in Smith et al. (2020) [14] (Table S6 and Table S7). In addition to Line WL1, Lines WL5 and RIR1 were also significant, as were the Across Lines tests. All 5 tested markers were significant or approached significance in WL1 (Table S10).

A single high LD block was found (Table S12). Interestingly, while Line WL5 was significant for 2 of the 4 markers significant in WL2 (MD14_02A, MD14_03A), no LD was found between them in that line. Similarly, the one significant marker in Line RIR1 (MD14_03A) had no LD with the other markers. These results suggest a single causative element in Lines WL1 and RIR1, while the situation in Line WL5 is not clear.

The tested markers in QTLR 34 were the genes *HN1L* (Hematological and Neurological Expressed 1 Like; also known as *JPT2*), *CRAMP1* (Cramped Chromatin Regulator Homolog 1), *TMEM204* (Transmembrane Protein 204) and *ATP6V0C* (ATPase H+ Transporting V0

Subunit C). In human, *HN1L* is involved in the endolysosomal trafficking of coronavirus

SARS-CoV-2, and is up-regulated in breast and uterine tumours [44]. *CRAMP1* is predicted to enable chromatin binding activity and to be involved in pattern specification process [45]. *TMEM204* is involved in cell adhesion and cellular permeability at adherens junctions [46]. *ATP6V0C* has a role in innate immunity and increased expression levels associated with various cancers [46].

The markers in this QTLR included one silent mutation in *HN1L* (MD14_01A), while the rest were intronic or 5’ flanking (Table S9). Thus, these markers are unlikely to be the causative mutations, although variation in a currently undefined regulatory element is possible.

# QTLR 35 on GGA17

QTLR 35 was found by the pooled genotyping in Line WL1, which was the only line showing significance with the individual genotyping (Table S10). A single Across Lines test was also significant. Only markers MD17_06A and MD17_07B were significant in Line WL1 but all 12 tested markers had *p* ≤ 0.062. In accordance with all markers having very similar *P*-values, a single high LD block was found, encompassing all markers tested in this line (Table S12), suggesting a single causative element.

The markers tested in this QTLR were from 9 genes (Table S9). The 2 significant markers reside in the genes *SH2D3C* and *TOR2A* (Torsin Family 2 Member A), while the one significant marker by the Across Lines test resides in *CDK10*. *CDK10* (Cyclin Dependent Kinase 10) and *CDK11* (Cyclin Dependent Kinase 11B) belong to the CDK subfamily of the Ser/Thr protein kinase family, known to be essential for cell cycle progression [47]. *CDK11b* has been associated in human with childhood endodermal sinus tumour and neuroblastoma [48]. *SH2D3C* encodes an adaptor protein and member of a cytoplasmic protein family involved in cell migration [49]. In human it has been associated with neuroinvasive eastern equine encephalitis virus infection [50]. *TOR2A* is involved in intracellular calcium concentrations and induces cell mitogenesis [51]. Thus, all significant genes in QTLR 35 are locational and functional candidates for affecting MD outcome.
