# Supplementary material for: Mapping quantitative trait loci regions associated with Marek’s disease on chicken autosomes by means of selective DNA pooling
Source: Sci Rep. 2024 Dec 30;14:31896. doi: 10.1038/s41598-024-83356-w (PMC11686186; doi:10.1038/s41598-024-83356-w)
Supplement: Supplementary file 3 — Supplementary Material 3 [file 41598_2024_83356_MOESM3_ESM.docx]

**Figure S1/supplementary figure 1.** Individually genotyped QTLRs.

The chromosome is presented on the top of each page; x-axis is location in Mb, y-axis is a marker -Log_10_P obtained by JMP Genomics SNP – Trait Association Trend test; each dot is a marker test in a line; QTLRs are presented as bars on the top of the charts.
